# Potential link between biotic defense activation and recalcitrance to induction of somatic embryogenesis in shoot primordia from adult trees of white spruce (*Picea glauca*)

**DOI:** 10.1186/1471-2229-13-116

**Published:** 2013-08-12

**Authors:** Robert G Rutledge, Don Stewart, Sébastien Caron, Cathy Overton, Bryan Boyle, John MacKay, Krystyna Klimaszewska

**Affiliations:** 1 Natural Resources Canada, Canadian Forest Service, Laurentian Forestry Centre, 1055 du P.E.P.S., Québec, QC G1V 4C7, Canada; 2 Centre for Forest Research and Institute for Integrative and Systems Biology, Université Laval, Québec, QC, Canada G1V 0A6

**Keywords:** Conifer, Gene expression profiling, Microarray analysis, Absolute qPCR, LRE qPCR, Clonal propagation

## Abstract

**Background:**

Among the many commercial opportunities afforded by somatic embryogenesis (SE), it is the ability to clonally propagate individual plants with rare or elite traits that has some of the most significant implications. This is particularly true for many long-lived species, such as conifers, but whose long generation times pose substantive challenges, including increased recalcitrance for SE as plants age. Identification of a clonal line of somatic embryo-derived trees whose shoot primordia have remained responsive to SE induction for over a decade, provided a unique opportunity to examine the molecular aspects underpinning SE within shoot tissues of adult white spruce trees.

**Results:**

Microarray analysis was used to conduct transcriptome-wide expression profiling of shoot explants taken from this responsive genotype following one week of SE induction, which when compared with that of a nonresponsive genotype, led to the identification of four of the most differentially expressed genes within each genotype. Using absolute qPCR to expand the analysis to three weeks of induction revealed that differential expression of all eight candidate genes was maintained to the end of the induction treatment, albeit to differing degrees. Most striking was that both the magnitude and duration of candidate gene expression within the nonresponsive genotype was indicative of an intense physiological response. Examining their putative identities further revealed that all four encoded for proteins with similarity to angiosperm proteins known to play prominent roles in biotic defense, and that their high-level induction over an extended period is consistent with activation of a biotic defense response. In contrast, the more temperate response within the responsive genotype, including induction of a conifer-specific dehydrin, is more consistent with elicitation of an adaptive stress response.

**Conclusions:**

While additional evidence is required to definitively establish an association between SE responsiveness and a specific physiological response, these results suggest that biotic defense activation may be antagonistic, likely related to the massive transcriptional and metabolic reprogramming that it elicits. A major issue for future work will be to determine how and if suppressing biotic defense activation could be used to promote a physiological state more conducive to SE induction.

## Background

Plant somatic embryogenesis (SE) has become an established biotechnology within the horticulture, agriculture and forest industries, providing the capability for commercial-scale production of clonal seedlings [1-3]. However, the efficiency of inducing embryogenic tissue formation (SE induction) continues to be problematic, particularly in woody species such as conifers. For example, although zygotic embryos from a few species belonging to the *Pinaceae* family are highly responsive, many other conifer species are either completely nonresponsive or produce efficiencies too low to be commercially viable. An even more prominent issue is the recalcitrance of tissues from adult trees, which, if overcome, would allow unlimited propagation of individual trees with elite characteristics [4].

While judicious manipulation of induction media has found success in improving SE induction efficiency from zygotic embryos, particularly for pines [3], successful application to vegetative tissues has to date been marginal, at best [4,5]. In addition, although many physiological and genetic factors impacting SE induction have been documented for angiosperms [6-8], lack of an effective experimental system has impeded efforts to identify even the most fundamental aspects underpinning SE induction within vegetative tissues of conifers.

In an attempt to address this deficiency, experiments initiated over a decade ago targeted somatic embryo-derived white spruce trees with the expectation that they would have a greater propensity for SE induction than trees grown from seed. This led to the identification of a clonal line of white spruce that produced shoot buds that have remained responsive to SE induction even after reaching sexual maturity [9]. Combined with advances in conifer genomics [10-12], this presented an unprecedented opportunity to explore the molecular aspects of SE induction within shoot primordia of adult spruce trees.

Using a recently constructed conifer 32 K oligo-probe microarray [12], transcriptome-wide expression profiling led to the identification of four of the most differentially expressed genes within this and a nonresponsive genotype at day 7 of induction. Expanding the analysis to day 21 using absolute qPCR revealed substantive differences in the expression dynamics of these candidate genes. Most evident was that both the magnitude and duration of candidate gene expression were greater within the nonresponsive genotype, which is indicative of an intense physiological response to the induction treatment that may be antagonistic to SE induction. Examination of their putative identities further revealed that this intense response may be a result of biotic defense elicitation, whereas the moderate response of the responsive genotype is suggestive of an adaptive response.

## Results

### Induction of somatic embryogenesis within primordial shoots

**Figure 1:**
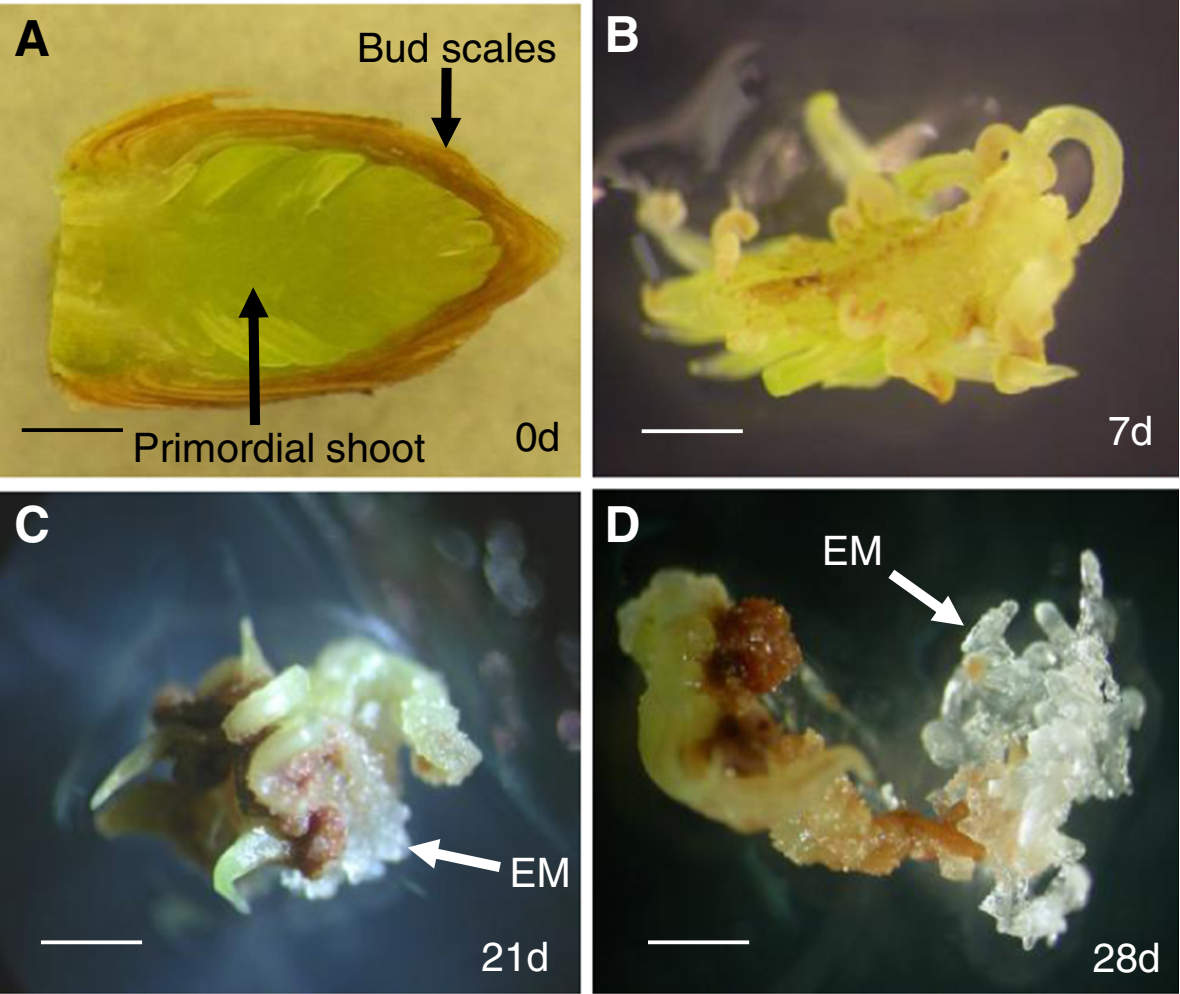
**SE induction within primordial shoots of adult white spruce trees. (A)** Longitudinally sectioned preflush bud representative of those collected for SE induction. **(B)** A shoot primordium explant following one week of induction treatment, the time point at which tissues were collected for microarray analysis. **(C)** Formation of embryonal masses (EM) that occurs within some of the G6 explants after about three weeks of induction. **(D)** Subsequent proliferation generates EM that can then be subcultured and used to generate unlimited numbers of seedlings that are clones of the parental tree from which the buds were collected. d: days of induction treatment, bars = 0.8 mm.

A detailed description of SE induction within primordial shoot explants of the responsive genotype (G6) has previously been described [9]. Briefly, buds were disinfected, primordial shoots excised and cut into sections before being placed onto SE induction medium (Figure 1A). With the expectation that differential gene expression could be associated with the responsiveness (or lack thereof) to SE induction, microarray analysis was conducted with RNA extracted from explants following one week of induction (Figure 1B). Selection of this time point was based on empirical observations suggesting that it was sufficiently early to avoid biases produced by embryonal mass formation, which could confound identification of genes associated with SE induction, rather than those that become active during embryogenesis. During the first two weeks of induction, explants of both G6 and that of a nonresponsive genotype (G12) were characterized by elongation of the needle primordia and formation of small amounts of callus on the cut surfaces and at the bases of elongated needle primordia. During the third week of induction, some of the G6 explants produced nodules on the elongated needle primordia or within the callus, along with minute amounts of embryonal masses (EM) that marked the initiation of SE (Figure 1C). During the fourth week of induction, some of the G6 explants generated rapidly proliferating EM (Figure 1D). After 16 weeks, 22 of 480 (4.6%) G6 shoot explants produced EM, while none of the 480 G12 explants responded.

### Microarray analysis and selection of candidate genes

**Figure 2:**
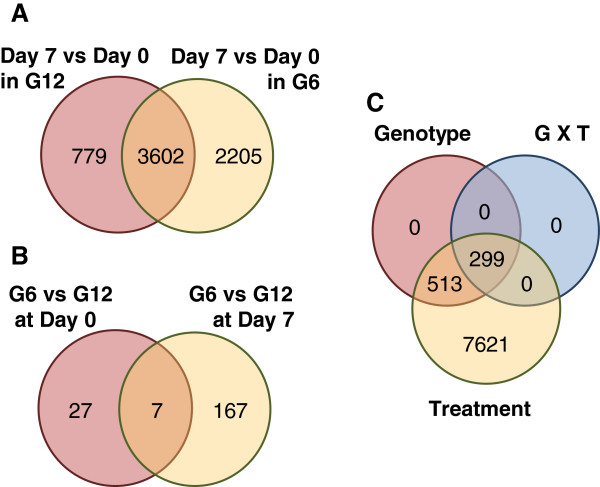
**Differential expression (DE) between genotypes and in response to one week of SE induction treatment. (A)** Intra-genotype DE produced by the seven day SE induction treatment (Student’s T-test; adjusted p-value <0.05). **(B)** Inter-genotype DE targets before and after the induction treatment (Student’s T-test; adjusted p-value <0.05). **(C)** DE in relation to genotype, treatment and interaction effects based on a two-way ANOVA (p-value < 0.05). No filtering was conducted based on the magnitude of fold difference and all statistical analyses were corrected for multiple testing.

The microarray analysis was conducted using explants taken at the point of collection (day 0) and after one week of SE induction (day 7), with five biological replicates analyzed for each genotype per time point (see Methods for additional details). Intra-genotype differences between day 0 and 7 were substantive, with 4381 and 5807 targets being differentially expressed within G12 and G6, respectively (Figure 2A). Although this includes 3602 targets that were shared, the total number of differentially expressed targets (6586) represents a sizeable proportion of the 23,854 distinct white spruce genes represented on the microarray [12]. An inter-genotype comparison reveals many small differences, with 167 targets differing significantly at day 7, as compared to 27 targets at day 0 (Figure 2B). Comparing the magnitude of intra-genotype fold-differences further supported the similarity of response to the induction treatment (Additional file 1). Such moderate differences suggest that the induction treatment generated a largely shared response with regards to the genes involved. However, this does not take into account quantitative differences in expression levels, an aspect that was examined during the qPCR analysis (see below).

To further investigate how genotype and the induction treatment interacted, a two-way ANOVA analysis was performed (Figure 2C). This revealed that 8433 targets were differentially expressed across all combinations, with about 90% responding solely in relation to the SE induction treatment. Furthermore, about 10% differed between the two genotypes, and about 3% showed a significant interaction between genotype and treatment. It is important to note that all targets showing a genotype effect also showed response to the SE induction, in which 37% showed a genotype X treatment interaction.

**Figure 3:**
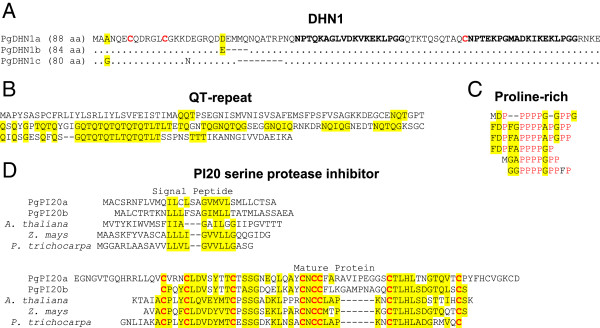
**Amino acid sequence alignments of the five most unusual candidate genes. (A)** DHN1, a conifer-specific dehydrin, was found to be composed of three variants (see Additional file 2 for more information). Conserved substitutions are highlighted in yellow and the two dehydrin domains are bolded. **(B, C)** QT-repeat and proline-rich candidates that appear to be unique to conifers, aligned to highlight their highly repetitive structure. **(D)** PgPI20a/b aligned with representative angiosperm homologs, which encode for highly conserved, small molecular weight serine protease inhibitors belonging to an unassigned subclass of the MEROPS I20 family (MER201390). *Populus trichocarpa* (EEF00358), *Arabidopsis thaliana* (At1G72060), *Zea mays* (EF406275). See Additional file 3 for amino sequence alignments of the remaining three candidates.

To identify candidate genes for qPCR analysis, the microarray data were sorted based on the largest fold differences relative to the other genotype at day 7 of induction (Additional file 1), which showed similar trends in both the number of targets and the magnitude of differential gene expression (Table 1). With the objective of selecting four candidate genes that most greatly differentiated each genotype at day 7, the most differentially expressed targets were examined in detail. This revealed that nine of the top 30 within G6 were found to be genes belonging to a small gene family encoding for three variants of an usual conifer-specific dehydrin called DHN1 (Figure 3A; Additional file 2), which has been identified previously in Norway spruce [13]. Due to their high degree of similarity, these DHN1 genes were considered to represent a single target (Table 2). Of the three remaining G6 candidates, a putative identity was found for only one, showing a high degree of similarity to the apoplastic class III peroxidase, AtPrx52, from Arabidopsis (Additional file 3). The last two candidates both encode for unusual proteins that appear to be conifer-specific, containing repetitive segments rich in threonine-glutamine and proline, respectively (Figure 3B, C).

**Table 1:** Greatest differential expression within G6 and G12 at day 7 of induction based on microarray analysis

**Day 7**	**G6 / G12**	**G12 / G6**
>3-fold difference:	32 targets	30 targets
>2-fold difference:	110 targets	142 targets
Top 10:	4.9-8.2-fold	3.9-9.6-fold

**Table 2:** G6 candidate genes showing the largest fold differences relative to G12 at day 7 of induction

** Acronym**	**Putative ID**	**G6 / G12**	**G6 / G12**	**G6**	**G12**	**UniGene**
		**Day 0**	**Day 7**	**Day 7/0**	**Day 7/0**	
DHN1a	Dehydrin	1.04	**5.29**	16.98	3.65	Pgl.27264
DHN1b						Pgl.27244
DHN1c						Pgl.12105
QT-repeat	Unknown	1.00	**5.45**	12.42	2.28	Psi.6570
PgPrx52	Class III Peroxidase	1.00	**4.34**	7.28	1.68	Pgl.27374
Proline-rich	Unknown	1.00	**3.57**	3.57	1.00	Pgl.22151

Fold differences between day 0 and 7 within each genotype are also listed for each candidate gene, along with their UniGene accession number. The DHN1 dehydrin candidate was found to be encoding for three closely related variants (Figure 3A, Additional file 2).

Putative identities were found for all four of the G12 candidates (Table 3). The top two were found to encode for closely related proteins with high levels of sequence similarity to an unusual class of serine protease inhibitor that is highly conserved throughout the Angiospermae, and predicted to have an amino terminal signal peptide based on SignalP 4.0 analysis [14] (Figure 3D). Another striking feature of these protease inhibitors is the presence of eight conserved cysteine residues that conform to the CRP5550 class of small cysteine-rich peptides, a very large family of excreted peptides that include defensins, along with many other antimicrobial proteins [15]. Like that of the G6 PgPrx52, the third G12 candidate encodes for an apoplastic class III peroxidase, but which is most similar to Arabidopsis AtPrx21 (Additional file 3). The remaining G12 candidate encodes for a cell wall invertase most similar to the Arabidopsis AtcwINV1 (Additional file 3).

**Table 3:** G12 candidate genes showing the largest fold differences relative to G6 at day 7 of induction

**Acronym**	**Putative ID**	**G12 / G6**	**G12 / G6**	**G12**	**G6**	**UniGene**
		**Day 0**	**Day 7**	**Day 7/0**	**Day 7/ 0**	
PgPI20a	Protease inhibitor	1.00	**9.56**	46.19	4.83	Pgl.12024
PgPI20b	Protease inhibitor	1.00	**8.89**	24.96	2.81	Pgl.12581
PgPrx21	Class III Peroxidase	1.00	**7.27**	9.24	1.27	Pgl.6064
PgcwINV1	Cell wall invertase	1.00	**6.94**	17.02	2.45	Pgl.11929

Fold differences between day 0 and 7 within each genotype are also listed for each candidate gene, along with their UniGene accession number.

With respect to changes in expression over time, comparing day 7 with day 0 revealed that all but one of the candidate genes increased significantly within both genotypes, with no example of a reduction in gene expression in the apposing genotype. This indicates that differential expression at day 7 was due to higher levels of activation within the originating genotype (Tables 2 and 3). Also notable is that the expression of all but one of the G12 candidates increased to greater levels within the G12 explants than that of the G6 candidates within the G6 explants, suggesting that a major distinguishing characteristic of the nonresponsive G12 genotype is higher levels of candidate gene activation.

### Concordance of microarray analysis with absolute qPCR

Conducting absolute quantification greatly increased the resolution of the analysis, in addition to allowing the expression of any gene to be directly compared with that of any other gene, within and between multiple samples. This was accomplished using a method developed by our group called LRE qPCR that greatly simplifies absolute quantification, in large part by abrogating the need to construct target-specific standard curves [16-18].

As is described in the Methods section, expression analysis of nine reference genes revealed that within the five biological replicates taken for each time point used in the microarray analysis, the average variance was found to be about ±20% (intra-sample group variance), which is in part indicative of the analytical precision that can be achieved with LRE qPCR [17]. Furthermore, when their average expression level was compared across the four sample groups, six of nine references generated inter-sample group variances below ±20%, reflective of a remarkably low level of biological variability within this experimental system. This in turn circumvented the need to conduct reference gene normalization as is commonly practiced for qPCR-based gene expression analysis, particularly for those employing relative quantification [19-22].

Table 4 summarizes the expression levels of the eight candidate genes, revealing that expression at day 0 was low for all the candidate genes within both genotypes, ranging from 5.6 to 534 transcripts per 10 ng RNA. Although the biological perspective of such a small dataset is necessarily limited, this does suggest that all of the candidate genes were relatively quiescent at the point of bud collection. In addition, consistent with that predicted by the microarray analysis, activation of the G12 candidates within the G12 explants was on average 9-fold higher than that of the G6 candidates within the G6 explants.

**Table 4:** Average transcript quantities of the candidate genes within the four sample groups used in the microarray analysis

**Target**	**G6 Explants**		**G12 Explants**	
	**Day 0**	**Day 7**	**Day 0**	**Day 7**
**G6**				
QT-repeat	204 ±58.8%	11,300 ±10.8%	22.6 ±74.3%	33.3 ±46.4%
DHN1	217 ±63.1%	32,500 ±60.8%	135 ±61.2%	7,890 ±47.5%
PgPrx21	34.7 ±59.2%	8,020 ±28.6%	8.0 ±78.8%	1,000 ±28.9%
Proline-rich	534 ±28.7%	4,120 ±35.0%	442 ±40.8%	1,730 ±13.3%
**G12**				
PgPI20a	16.8 ±117.3%	4,290 ±60.2%	155 ±81.8%	193,700 ±44.7%
PgPI20b	5.6 ±108.3%	8,080 ±79.0%	55.8 ±79.0%	162,500 ±40.2%
PgPrx52	63.0 ±32.4%	583 ±81.4%	179 ±51.7%	27,100 ±27.8%
PgcwINV1	34.3 ±47.8%	18,065 ±60.0%	8.6 ±48.2%	122,100 ±56.6%

Quantities are expressed as the number of transcripts per 10 ng RNA ± CV (coefficient of variation = (standard deviation/average) x 100%). Individual quantifications are provided in Additional file 7.

Table 5 compares the relative differences in candidate gene expression between the two genotypes at day 7, which provides broad confirmation of the microarray analysis. For example, the relative ranking based on the magnitude of fold differences, as predicted by microarray and qPCR quantification, is in general agreement within and between the two groups of candidate genes. One obvious exception is the qPCR-derived ratio for the QT-repeat candidate of 340-fold. However, this is a result of the very low expression levels within the G12 explants at day 7 (Table 4), bringing into doubt the comparability with the microarray analysis. These datasets also reflect the limited biological perspective that can be achieved with analysis of only two time points. A major aspect of this study was thus to exploit the high capacity of LRE qPCR to expand the analysis to day 21 of induction.

**Table 5:** Fold differences in candidate gene expression as determined by microarray and absolute qPCR quantification

**G6/G12**			**G12/G6**		
**Target**	**Microarray**	**qPCR**	**Target**	**Microarray**	**qPCR**
QT-repeat	5.45	340	PgPI20a	9.56	45.1
DHN1	5.29	4.12	PgPI20b	8.89	20.1
PgPrx21	4.34	8.02	PgPrx52	7.27	46.5
Proline-rich	3.57	2.38	PgcwINV1	6.94	6.76

### Profiling the dynamics of candidate gene expression

**Figure 4:**
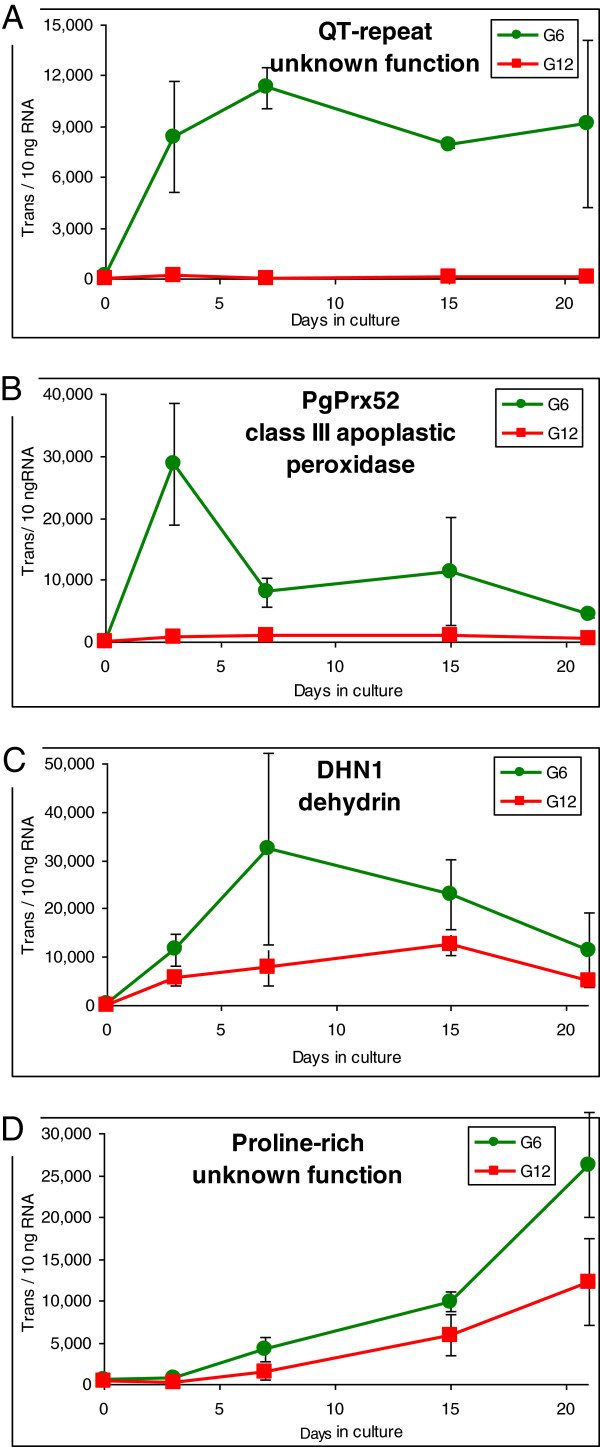
**Expression profiling of the G6 candidate genes during 21 days of SE induction.** Each point represents the average quantity expressed as the number of transcripts per 10 ng RNA with the standard deviation presented as bars. Individual quantifications are provided in Additional file 7.

Expanding the analysis to day 21 by including three additional time points (3, 15 and 21 days) allowed the dynamics of candidate gene expression to be defined in greater detail. For example, within the G6 explants the QT-repeat candidate expression reached near maximal levels by day 3, a level that was maintained up to day 21, whereas within the G12 explants, its expression was nearly absent throughout the entire induction treatment (Figure 4A). Extensive differential expression was also revealed for the apoplastic peroxidase PgPrx52 within the G6 explants, reaching maximal expression by day 3, but falling 3-fold by day 7, to a level that was maintained up to day 21 (Figure 4B).

The dynamics of DHN1 expression was similar in nature to PgPrx52, peaking at day 7 followed by a progressive 3-fold reduction by day 21 within the G6 explants, indicative of an early, transient-like activation (Figure 4C). However, DHN1 expression was not only apparent within the G12 explants, but progressively increased up to day 15, suggesting that activation of this G6 candidate gene is much less genotype-specific. While differential expression of the proline-rich candidate was maintained up to day 21, both genotypes generated similar expression dynamics, again reflective of modest, if any, genotypic specificity (Figure 4D).

**Figure 5:**
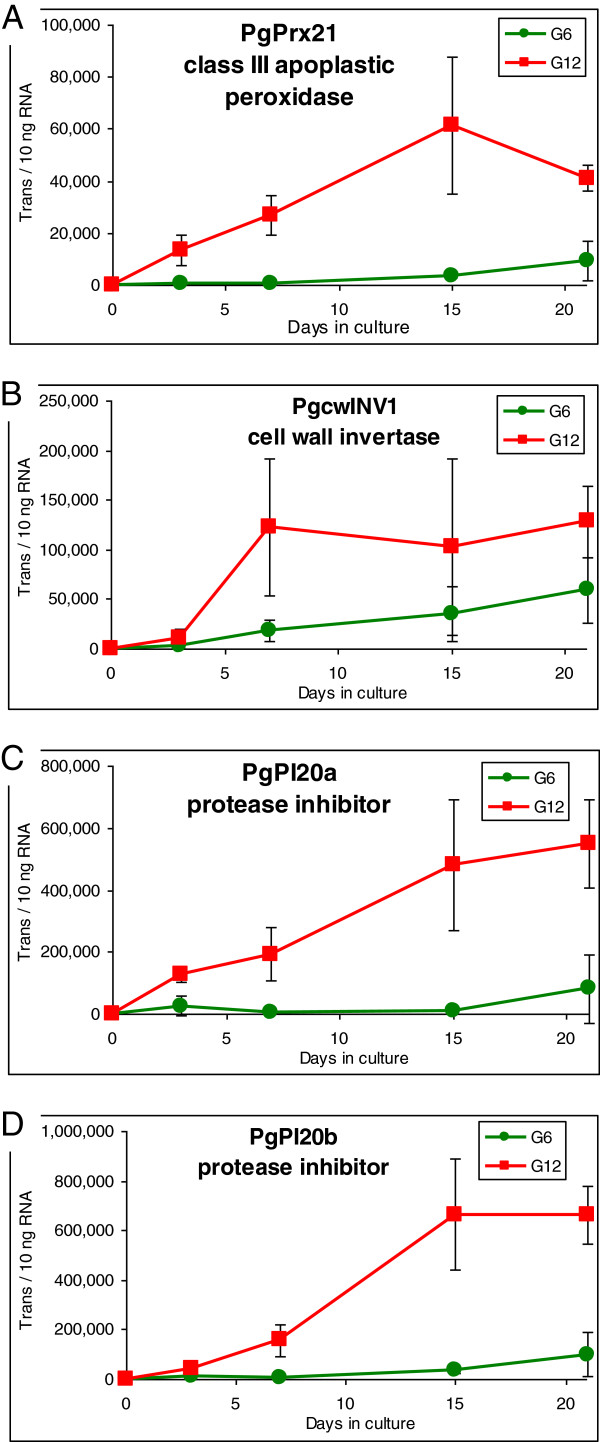
**Expression profiling of the G12 candidate genes during 21 days of SE induction.** Each point represents the average quantity expressed as the number of transcripts per 10 ng RNA with the standard deviation presented as bars. Individual quantifications are provided in Additional file 7.

For G12, all four candidates demonstrated high levels of differential expression (Figure 5). In addition, expression for all but PgcwINV1 progressively increased during the induction treatment, all reaching maximal levels that were on average about 20X greater than the maximum expression of the G6 candidates within the G6 explants (Figure 4). Overall, the expression dynamics within the G12 explants is consistent with an intense and sustained response to the induction treatment.

In summary, qPCR expression profiling confirmed the efficacy of candidate gene selection based on microarray analysis at day 7 of induction, in that all of the candidate genes maintained differential expression within their originating genotype throughout the entire induction treatment, although to varying degrees. In addition, the overall biological variability was sufficiently low to reveal strong trends in gene expression dynamics, and to illustrate the utility of absolute quantification, which, among other attributes, provides the ability to assess the magnitude of expression of individual genes. Also consistent with that predicted by the microarray analysis was the high level of induction of all four G12 candidates within the G12 explants, which was maintained well beyond day 7. This suggests that a major distinguishing characteristic of these nonresponsive explants is an intense physiological response to the SE induction treatment.

## Discussion

Recalcitrance of plant explants to many types of tissue culture manipulation, including SE induction, has long been an impediment to clonal propagation of individual plants with elite characteristics, a capability that has significant commercial implications. These also include rare traits, such as insect or pathogen resistance, for which rapid propagation and dissemination could have important ecological implications. This is particularly relevant to forest trees, whose long generation times pose substantive challenges to traditional propagation approaches such as rooting of cuttings, and to genetic improvement programs based on conventional breeding. These limitations are further exacerbated by the fact that many elite traits become most evident in mature plants, a time at which many woody species, including conifers, become recalcitrant to tissue culture manipulation [4]. The identification of a clonal line of adult white spruce trees that produce shoot primordia responsive to SE induction thus presented a unique opportunity to address the molecular aspects of SE induction, with the expectation that this may be broadly applicable to other plant species.

### Stress-response as a likely determinant for SE induction responsiveness

As is often the case for SE-induction research, a major presumption entering into this study was that genes selectively expressed within the responsive G6 bud explants would become the primary targets for investigation. Indeed, expression profiling revealed an unusual glutamine-threonine repeat protein (Figure 3B) that was specifically expressed throughout the entire SE induction treatment (Figure 4A). Although such a dramatic differential expression implies a potentially important role in induction responsiveness, this protein appears to be conifer-specific with no known function, excluding the likelihood that this protein plays a universal role in somatic embryogenesis. This is also the case for a proline-rich protein (Figure 3C), which showed marginal, albeit persistent, differential expression in the G6 bud explants up to day 21 (Figure 4D); however, this protein also appears to be conifer-specific with no known function. Although the two remaining candidates encode for proteins with putative functions (dehydrin and peroxidase; Figure 3A and Additional file 3, respectively), the greatest differential expression occurred during early stages of the induction treatment (Figure 4B, C), long before embryogenic tissues begin to emerge, which again does not greatly support a role, at least directly, in determining SE induction responsiveness.

In contrast to this temperate response, the intense candidate gene activation within the nonresponsive G12 bud explants was not only found to persist into the late stages of the induction treatment, but also reached very high levels (maxima 60,000-600,000 transcripts per 10 ng RNA) (Figure 5). While the limited use of absolute quantification has to date provided an inadequate context for universally comparing gene expression levels, EF1α does provide some perspective as to the magnitude of candidate gene expression. Utilized as a reference gene, EF1α expression averaged around 700,000 transcripts per 10 ng RNA (see Methods), which is the most highly expressed gene encountered in this study. This is consistent with it being one of the most highly expressed genes in developing conifer shoots, as based on EST clone frequency and microarray analysis (data not shown). It is thus the magnitude of candidate gene expression within the nonresponsive G12 explants that suggests the physiological response of a primordial shoot explant, particularly during the early stages of SE induction, could determine its ability to become responsive to SE induction.

While the subject area of stress physiology is vast, it is apparent that a number of general principles described for angiosperms could provide insights into the physiological response of conifer bud explants, particularly in relation to abiotic and biotic defense responses. For example, a transient oxidative burst of low intensity is predicted to occur immediately after an explant is introduced into culture, which is indicative of early oxidative signaling generated during both abiotic stress (including wounding) and biotic defense responses [23-25]. A major distinction, however, is that elicitation of a biotic defense response is associated with a second prolonged oxidative burst of high intensity, which in turn triggers massive transcriptional and metabolic reprogramming, including high level induction of defense protein expression, slowing of growth, and, in the most extreme cases, induced cell death [23,24,26-29]. This possess the question as to whether the intense response generated by the G12 explants is reflective of a biotic defense response that makes them physiologically recalcitrant to SE induction, in contrast to an adaptive stress response within the G6 explants that generated a physiological state conducive to SE induction.

Although such generalizations provide an attractive model, it should be stressed that angiosperm defense responses have been found to be highly dynamic and complex processes that involve cross-talk between signaling networks regulated by salicylate, jasmonates, and ethylene, in combination with other plant hormones [27-32]. It is therefore difficult to draw specific parallels to conifer bud explants without direct supporting evidence. Nevertheless, examining the putative functions of the proteins encoded by the four G12 candidate genes (an apoplastic class III peroxidase, a cell wall invertase, and two closely related extracellular serine protease inhibitors) provides support for the contention that the SE induction treatment elicited a biotic defense response within these nonresponsive explants.

### Apoplastic class III peroxidases play a prominent role in biotic defense activation

Class III peroxidases have been found to generate apoplastic hydrogen peroxide that acts as a signal for biotic defense elicitation [27,33-36]. Direct demonstration of this was recently reported for maize, in which *U. maydis* (corn smut) leaf infection was found to induce the expression of a single class III peroxidase gene (POX12), and that virulence of this pathogen is dependent on its excretion of a peroxidase inhibitor peptide (Pep 1) [37,38]. Furthermore, induced gene silencing of POX 12 was found to restore virulence even in the absence of Pep 1, providing direct evidence that POX12 activity is essential for mounting a successful biotic defense response against this pathogen [38]. The progressive, high level activation of a class III peroxidase within the G12 explants (Figure 5A) is thus consistent with a role in biotic defense elicitation.

Additional, albeit tentative, support for such a contention is that this G12 candidate is most similar in amino acid sequence to the Arabidopsis class III peroxidase, AtPrx21 (Additional file 3), which belongs to an unusual evolutionary branch of plant peroxidases [39]. Increased expression of AtPrx21 produced by wounding and microbial attack has led to the suggestion that it has a protective role against pathogens [36]. A more direct demonstration of a role in biotic defense comes from the fact that overexpression of AtPrx21 in Arabidopsis produces resistance to *Botrytis cinerea*[40]. Thus, currently available data suggests that persistent, high level expression of an apoplastic class III peroxidase gene plays a central role in biotic defense activation, a role that the G12 PgPrx21 may play in conifers.

Conversely, the transient nature of the G6 class III peroxidase PgPrx52 gene activation (Figure 4B) could be reflective of an adaptive stress response in which the initial oxidative burst dissipates during the first few days of induction treatment, restoring cellular redox homeostasis [41-43]. This is similar to the activation, although slightly later, of the G6 dehydrin DHN1 gene (Figure 4C), which is a conifer-specific dehydrin that has been reported to play a role in bud dormancy and overwintering in Norway spruce [13]. This too supports the contention that the G6 explants elicited an adaptive stress response, in that dehydrins have long been recognized as playing a fundamental role in adapting to environmental stresses [44]. Thus, although speculative, it could be argued that an adaptive stress response may be an important determinant for establishing SE induction responsiveness.

### Induction of a cell wall invertase

Activation of cell wall invertases, which catalyze the hydrolysis of sucrose to glucose and fructose within the apoplast, have also been found to play a prominent role in biotic defense by providing the large quantities of energy required for mounting an intense metabolic response [45-49]. RNAi inhibition of expression has provided direct support for a central role of a cell wall invertase in defense response elicitation in tobacco [50]. Indeed, the prominence of cell wall invertase induction has led to a proposal that it be classified as a pathogenesis-related protein [45], which is a large class of defense proteins that become highly expressed following biotic defense activation [51].

Amino acid sequence comparison to Arabidopsis revealed that the G12 invertase is most similar to AtcwINV1 (also called AtβFruct1) (Additional file 3), which is a member of a small gene family within Arabidopsis [47]. A number of studies have reported induction of AtcwINV1 expression following pathogen infection of Arabidopsis leaves [52-54], in addition to induction following Agrobacterium infection of Arabidopsis cell cultures [55]. Importantly, selective induction of AtcwINV1 following wounding also suggests a general role in adaptive stress responses [56]. This is consistent with the progressive increase in expression of PgcwINV1 observed within the G6 explants. However, this is contrasted by a rapid activation within the G12 explants between day 3 and 7, reaching an average of about twice that found within the G6 explants, a level that is maintained up to day 21 of the induction treatment (Figure 5B). This could be reflective of a more intense metabolic response of the G12 explants during the earliest stages of the induction treatment, a presumption consistent with the high levels of expression observed for the two remaining G12 candidate genes.

### Activation of two I20 serine protease inhibitors

The two most highly expressed G12 candidate genes were found to encode for closely related protease inhibitors belonging to an unassigned subclass of the MEROPS I20 family of serine protease inhibitors [57,58]. This subclass has a number of distinguishing features, including the presence of a transient peptide, an unusually small mature protein (less than 60 amino acids), and the presence of eight highly conserved cysteines (Figure 3D). In fact, these features have led to their classification into the superfamily of small cysteine-rich peptides (CRP), a very large family of secreted peptides composed of several hundred genes within Arabidopsis. Initially founded on structural similarities with defensins, which is an ancient form of antimicrobial peptide [59], a large number of CRPs have been shown to play a role in biotic defense [15]. Another distinctive characteristic of this subclass of I20 protease inhibitors is the occurrence of closely related homologues throughout the Angiospermae, principally as a single gene [60]. In fact, this high level of conservation led Hartl et al. [60] to suggest that in addition to a probable role in biotic defense, they could have an essential role in plant physiology. The presence of two highly conserved homologs within conifers provides support for such a possibility.

A direct link of the angiosperm homolog to biotic defense activation comes from expression analysis of *U. maydis* Δpep1-infected maize leaves, which elicits a massive biotic defense response, as was described earlier. Among the 220 genes found to be induced, the maize I20 homolog (EF406275 in Figure 3D) was the fourth most highly induced, increasing by 166-fold within 24 h post-inoculation (Additional file 1: Table S1 in [37]). Similarly, the Arabidopsis I20 homolog (At1G72060) was the fifth most induced gene following infection by *Trichoderma harzianum*, reaching a 2.79-fold increase within 24 h [61]. Application of biotic defense elicitors to 10-day-old Arabidopsis seedlings was also found to induce this I20 gene, with the bacterial flagellin peptide Flg22 generating 5.08- and 7.45-fold increases after 1 and 3 h, respectively (Additional file 1: Table S1 in [62]). Oxidative stress induction of the maize homolog was also demonstrated in maize leaves (referred to as serpin), reaching 2.91- and 7.56-fold increases 16 h after application of H_2_O_2_ and methyl viologen, respectively [63]. However, a role in abiotic defense is unlikely, as ectopic expression of the Arabidopsis homolog in transgenic seedlings dramatically reduced resistance to oxidative, osmotic and salt stress [64].

The induction kinetics of the two conifer I20 homologs within the G12 explants is thus consistent with biotic defense activation as observed in angiosperms, with both genes reaching close to 200,000 transcripts per 10 ng RNA by day 7, which, in comparison with day 0, roughly represents a 2000-fold increase. Another notable feature was a progressive increase in expression throughout the entire induction treatment, reaching about 600,000 transcripts per 10 ng RNA by day 21 (Figure 5C and D). Thus, while the precise biochemical function of this unusual class of serine protease inhibitors remains to be determined, the high level of amino acid sequence conservation, combined with their expression dynamics, provide support to the supposition that these two conifer protease inhibitors play a role in conifer biotic defense, similar to that observed in angiosperms.

### A paradigm shift towards physiological processes that may antagonize SE induction

While it has long been recognized that the physiological state of an explant can be a major determinant for responsiveness to SE induction, very little is understood about the underlying mechanisms. Furthermore, the vast majority of research efforts have historically focused on defining factors that promote SE induction, for example, through the activation of SE-promoting genes. This study expands this paradigm, suggesting that nonresponsiveness of an explant is not necessarily due to an innate lack of SE promoting activity, but that biotic defense activation could potentially be a dominant antagonist.

A number of physiological aspects of biotic defense elicitation provide general support for such a presumption. For example, activation of biotic defense through exogenous application of various elicitors has been shown to dramatically reduce plant growth, a result of redirecting metabolic energy from growth to defense [65,66]. This includes the action of jasmonates (JAs), the central regulators of biotic defense elicitation associated with wounding, which have been shown to directly mediate a switch from growth to the production of biocidal compounds, cell-wall remodeling, and defense protein expression [28]. Indeed, growth repression by JAs has been directly linked to inhibition of cell-cycle progression [67], in addition to directly antagonizing the growth promoting activity of gibberellic acid [31], thus providing evidence that JAs could be direct antagonists of SE induction, based in part on the assumption that cell division is necessary for embryogenic tissue formation.

Another line of supporting evidence, albeit indirect, comes from proteomic studies that have reported a correlation between expression of biotic defense proteins, primarily pathogenesis-related proteins, and a lack of embryogenic competency of tissues in culture [68-71]. Another notable observation related to the quantitative nature of proteomic analysis, is the magnitude of defense protein expression within these nonembryogenic tissues, often being the most prominent proteins in the analysis. While it is difficult to draw a direct comparison, this is consistent with the intense activation of the G12 candidate genes within the G12 explants, and with the supposition that redirecting metabolic resources towards the production of such large quantities of defense proteins could itself be antagonistic to the formation of embryogenic tissues.

SE induction within leaf explants from the model legume *Medicago truncatula*, an experimental system similar to the bud explants used in this study, has also been used to directly compare responsive and nonresponsive genotypes [72]. Proteomic analysis revealed large physiological differences as reflected by high levels of protein accumulation, some of which were identified as stress proteins, within a nonresponsive line during the first week of the SE induction treatment. Although an association with biotic defense elicitation was not evident from the data presented, this led the authors to suggest that a hyperresponse to the stress produced by the induction treatment could be related to a lack of responsiveness [73]. This is a scenario similar to the intense physiological response of the G12 explants, and reminiscent of the high levels of defense protein accumulation within nonembryogenic tissues, as discussed above.

Finally, although this study provides evidence for a link between biotic defense elicitation and recalcitrance to SE induction, it has also generated hypotheses that could not be directly addressed within the experimental design. First, only one nonresponsive genotype was analyzed, so the question as to whether a similar physiological response occurs in other nonresponsive genotypes remains unanswered. Second, the hypothesis of a direct association between biotic defense elicitation and suppression of SE induction remains to be tested. Thus, it is possible that the differences in physiological response observed within the G6 and G12 explants is a result of a genotypic difference unrelated to SE responsiveness. Third, it is unknown whether SE could be induced in the G12 explants even if biotic defense elicitation were to be mitigated. Additional work is thus required before a definitive understanding of the broad applicability and implications of these findings can be achieved.

## Conclusions

The central conclusion of this study is that the physiological response of conifer bud explants, particularly in relation to elicitation of a defense response, could be an important determinant of SE induction responsiveness. Although definitive demonstration that biotic defense activation is antagonistic to SE induction requires additional evidence, many general characteristics, such as the dramatic metabolic and transcriptional reprogramming associated with its elicitation, support a role contributing to the recalcitrance of explants to SE induction. In addition, it opens new avenues of investigation into the mechanisms regulating the activation and intensity of defense responses within explants placed into culture, along with the prospective of developing methods that could be used to suppress them, with the expectation that this could generate a physiological state more conducive not only to SE induction, but potentially to other types of tissue culture manipulation.

## Methods

### Primordial shoot collection and somatic embryogenesis induction

Shoot buds were collected on May 4 and 6, 2009, from the second and third whorls of branches of 9-year-old *Picea glauca* (white spruce) trees that were generated from somatic embryos as previously described [9]. These consisted of a responsive (893-6: G6) and nonresponsive (893-12: G12) genotype, from which a total of 700 shoot buds were collected from several clonal trees per genotype. SE induction was conducted as previously described [9]. Briefly, the buds were disinfected, primordial shoots were excised and cut longitudinally into two or four equal parts, and the explants were placed onto semi-solid MLV-S medium containing 9.5 μM 2,4-dichlorophenoxyacetic acid and 4.5 μM benzyl adenine. Replicate explant samples were collected after 3, 7, 15 and 21 days of culture, frozen in liquid nitrogen and stored at −80°C. All remaining explants were cultured for 16 weeks, during which formation of embryonal masses was verified under a stereomicroscope and recorded.

### RNA preparation

Two rounds of RNA extraction were conducted, referred to as sample series 1 and 2. For sample series 1, which was used in the microarray analysis, a CTAB-LiCl precipitation protocol [74] was used to extract RNA from five biological replicates of both genotypes, consisting of approximately 80 mg of buds that were either placed into liquid nitrogen immediately after collection in the field (day 0), or after one week of SE induction treatment (day 7). For qPCR analysis, aliquots of these RNA samples were DNase treated before cDNA production, as described in the reverse transcription section.

For sample series 2, three replicate collections were taken at day 3, 15 and 21 of induction, with each replicate consisting of approximately 80 mg of fresh mass. These were placed into a 2-ml Sarstedt conical microtube containing a single 5 mm stainless steel bead, frozen in liquid nitrogen, and stored at −80°C. However, it should be noted that subsequent work has revealed that smaller amounts (less than 50 mg) can significantly improve both the quality and quantity of RNA recovered for some sample types. The tubes were transferred into an adapter set that was prechilled at −80°C for a minimum of two hours and transported in a cooler containing a few inches of liquid nitrogen in order to prevent the samples from thawing. The tissues were disrupted twice for 45 s at 26 Hz using the Qiagen Tissuelyzer II bead mill. The adapter set was returned to the cooler, and each tube was removed one at a time. In each tube 550 μl of lysis buffer (4 M guanidine isothiocyanate, 0.2 M sodium acetate, pH 5.0, 25 mM EDTA, 2.5% (wt/vol) PVP-40) was added [75]. The tubes were vortexed at high speed and incubated for 2 min at 56°C, during which one or two more vortexing steps were conducted. Samples were then centrifuged briefly to remove cell debris and 450 μl were removed for RNA extraction using the Qiagen RNEasy plant mini kit (Cat. # 74904). RNA extractions were performed using a Qiacube DNA/RNA purification robot (Qiagen), which included an on column DNase treatment (Qiagen RNase free DNase, Cat. # 79254). However, variable quantities of genomic DNA were subsequently detected by qPCR, such that a second DNase treatment was necessary (see the reverse transcription section for details). Following this DNase treatment, RNA was quantified using a Nanodrop 1000 Spectrophotometer (Thermo Scientific), and RNA integrity was assessed using the Agilent 2100 Bioanalyzer (Agilent Technologies), which generated RIN values of 8.2-9.8.

### Microarray analysis

Microarray experiment and analysis were conducted as previously described [12], with minor modifications. Briefly, 1 μg of each total RNA sample was amplified using the Amino Allyl MessageAmpII aRNA Amplification Kit (Life Technologies, Carlsbad, CA, USA), fragmented and quantified, and 5 μg of amplified RNA was labeled with AlexaFluor 647 (Life Technologies). Prehybridization of the oligonucleotide arrays, hybridization of the labeled samples to the slides, slide washing and drying were performed on HS400Pro hybridization stations (Tecan Group Ltd., Männedorf, Switzerland). Slide scanning and feature extraction were done on a ScanArray Express scanner (PerkinElmer, Waltham, MA, USA) and QuantArray v3.0 (PerkinElmer), respectively. Each array image was analyzed in two sections (top and bottom half), and both sections were fused into one file in Excel using an in-house macro. All of the arrays (20) were kept for further analysis. The experiment was analyzed as a one-color design with four groups of five arrays (two time points and two genotypes, 5 biological replicates). Quality control and data processing, namely background and buffer subtraction, aQuantile normalization and correction for multiple testing (Benjamini-Hotchberg), were done in R version 2.8.1 [76]. All microarray data have been deposited in NCBI’s Gene Expression Omnibus and are accessible through GEO Series accession number GSE46977. Genes were selected as being differentially expressed on the basis of their adjusted p-values (p-value < 0.05). Analysis of variance and t-tests of selected genes were conducted using Flexarray v1.6 [77].

### Candidate target selection and primer design

Candidate targets were selected based on fold differences in expression between the two genotypes at day 7 of SE induction with no consideration of target gene identity (Tables 2, 3; Additional file 1). A secondary consideration was similar expression within the two genotypes at the point of bud collection (day 0), which led to the rejection of one G12 and six G6 targets. The probe sequence from each candidate target was blasted against the NCBI *Picea* EST libraries and nucleotide collection databases, from which nucleotide alignments were constructed for primer selection (Additional files 4 and 5), which was based primarily on positioning the amplicon close to the stop codon in order to minimize variance due to partial reverse transcription.

The central parameter used for primer design was determining a length sufficient to generate a predicted T_m_ of 70°C using the Integrated DNA Technologies online OligoAnalyzer. This was based on calibrating the program by arbitrarily adjusting the Mg^++^ parameter concentration to 50 mM, such that the predicted T_m_ of CAL1 F1 and CAL1 R1 primers (Additional file 5) reached 70°C, an approach found successful for other oligo design programs. Following calibration, candidate primers were then designed by simply adjusting their length until the predicted T_m_ just exceeded 70°C. Extensive self-complementary primers or those complementary to the apposing primer were rejected, as were any primer pair that generated non-specific products in a no template control amplification or that generated amplification efficiencies <99%. Amplicon size was restricted to 80–200 bp; however, extensive efforts to predict primer performance, such as analyzing the secondary structure of the primers or of the resulting amplicon, were unsuccessful. It was therefore necessary to test multiple primer pair combinations for some targets, rejecting those that generated profile collapse or extensive plateau drifting as indicated by LRE analysis (see [18] for details about these anomalies).

### Reverse transcription

Before conducting reverse transcription, genomic DNA contamination was quantified by amplifying 20 ng samples of raw RNA. This revealed that many samples contained small amounts of gDNA contamination (10–100 genomes) so that all RNA samples were DNase-treated using the Ambion Turbo DNA-free DNase kit (Cat. # AM1907), which was found to reduce gDNA contamination to undetectable levels.

Reverse transcription was conducted in 20 μl reactions containing 50 ng/μl RNA, 25 ng/μl oligo dT primer (Invitrogen, Cat.# 18418–012), 5 U/μl Superscript II (Invitrogen, Cat. # 18064–014) using the manufacturer’s supplied buffer, and incubated at 42°C for 50 min, followed by the addition of 180 μl 10 mM Tris, pH 8.0 to generate a final concentration of 5 ng RNA per μl. Testing revealed that the commonly practiced addition of RNase inhibitor and DTT provided no benefit. Note also that we have found that RNase H treatment produces extensive scattering of replicate profiles for some cDNA targets, which can greatly reduce quantitative accuracy. As previously reported, three replicate reverse transcriptase reactions using an identical RNA sample, generated an average variance of about ±12% for three reference gene targets [18], indicating that this method is highly repeatable, consistent with the small variances in reference gene expression observed in this study (see below).

### LRE qPCR

Detailed descriptions of how LRE qPCR was developed, its performance capabilities, and description of a platform-independent Java desktop program that automates LRE analysis have previously been published [16-18,78]. In brief, absolute quantification is achieved by converting 3-6 of the fluorescence readings within the central region of each amplification profile into target quantity expressed in fluorescence units (F_0_). These are averaged and converted into DNA mass (M_0_) using an optical calibration factor (OCF = fluorescence units per ng dsDNA) generated by amplification of a known quantity of lambda genomic DNA, an approach analogous to that used for quantification of nucleic acids using fluorescent dyes. This is followed by conversion into the number of target molecules (N_0_) based on amplicon size (A_s_) [17]:

PCR amplification was conducted with an Applied Biosystems 7500 Fast qPCR system (normal ramping), QuantiTect enzyme formulation (Qiagen, Cat. # 204145) in a 10 μl reaction volume containing 500 nM of primers, an aliquot of reverse transcriptase reaction equivalent to 5 ng RNA, using 96 well BrightWhite plates (Primerdesign, BW-Fast) sealed with MicroAmp film (Applied Biosystems, Cat # 4311971). The cycling regime consisted of a 15 min activation at 95°C, followed by 50 cycles of 95°C for 10 s, and 65°C for 120 s. Amplicon T_m_ was determined for each amplification reaction by melt curve analysis (65 to 90°C) conducted at the end of each run. Raw fluorescence readings were imported into the LRE Analyzer (Version 0.8.7), an open source Java program available for download [79,80]. The program contains an extensive help dataset that describes how the program functions, along with various guidelines for setting up and testing the performance of qPCR assays.

All primer pairs produced a single prominent amplicon peak following melt curve analysis, and generated amplification efficiencies that ranged from 102 to 107%. The propensity for QuantiTect to generate amplification efficiencies greater than 100%, a systemic bias that generates moderate underestimates of target quantity, was compensated by fixing the amplification efficiency to 100%, as described in the LRE Analyzer help. The LRE Analyzer databases are provided in Additional file 6, along with the amplicon and optical calibration databases used in this study. A summary of the individual quantitative determinations is provided in Additional file 7.

### Reference gene analysis

Expression of nine reference genes was used to determine the levels of biological variability, in addition to serving as internal quality controls for assessing the technical variance associated with sample preparation and LRE qPCR analysis. Two reference genes were taken from microarray analysis of Sitka spruce apical shoots (hypothetical protein (HP) and peroxisomal targeting signal receptor (PTSR)) [81], with the remaining seven being conifer homologs to Arabidopsis reference genes also identified from microarray analysis [82]. Primer sequences along with UniGene accession numbers are provided in Additional file 5.

**Figure 6:**
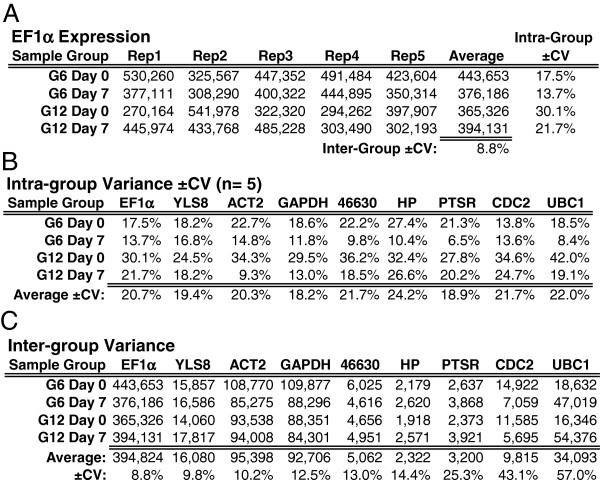
**Reference gene expression analysis within sample series 1: day 0 and 7. (A)** Representative example of intra- and inter-group variance determination based on the coefficient of variation (±CV) based on EF1α expression. **(B)** Intra-group variance of all nine reference genes. **(C)** Inter-group variation based on the average transcript quantities as illustrated in **(A)**. Quantities in **(A)** and **(C)** expressed as transcripts per 10 ng RNA. ±CV = (standard deviation/average) x 100%.

Assessing expression stability was based on coefficient of variation, analogous to the approach used to develop the Genevestigator’s RefGenes tool, in which transcriptome-wide expression stability was assessed using the standard deviation of signal intensities generated by microarray analysis [83]. Figure 6A provides an example of this approach based on EF1α expression within sample series 1 (day 0 and 7). Intra-group variance is a combination of biological variability and technical-derived variance associated with RNA preparation, cDNA production and LRE qPCR analysis, whereas inter-group variance is primarily reflective of biological variance. Expanding the analysis to nine reference genes generated similar intra-group variances (Figure 6B), with inter-group variances differed more greatly (Figure 6C).

**Figure 7:**
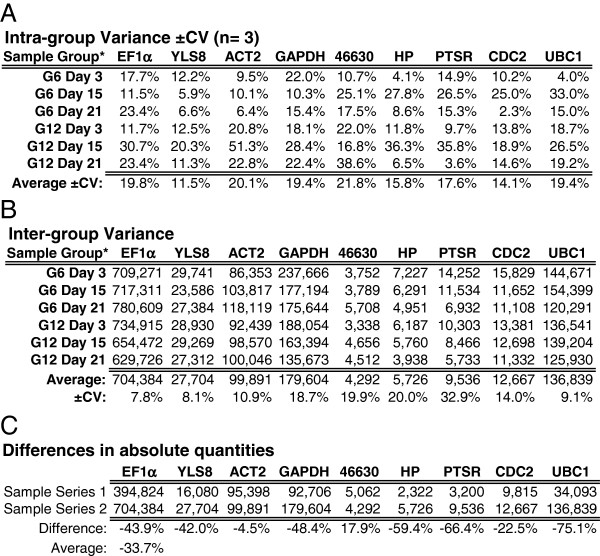
**Reference gene expression analysis within sample series 2: day 3, 5 and 21. (A)** Intra-group variation (±CV). **(B)** Inter-group variation based on the average transcript quantities as illustrated in Figure 6A. **(C)** Differences in the average absolute quantities derived from sample series 1 and 2. All quantities are expressed as transcripts per 10 ng RNA. ±CV = (standard deviation/average) x 100%.

Repeating the analysis with sample series 2 (day 3, 15 and 21) generated similar intra-group variations (Figure 7A). Inter-group variances were also similar, except for CDC2 and UBC1 (Figure 7B), which were much lower than those observed in sample series 1 (Figure 6C). Another notable outcome is that despite the high levels of expression stability, nearly all of the reference genes within the sample series 1 produced average absolute quantities lower than those of sample series 2 (Figure 7C). Although the source of these differences was not investigated, it may be related to the LiCl precipitation step used to prepare the RNA within sample series 1. Regardless, based on the premise that such an anomaly would be modest in relation to the large changes observed in candidate gene expression, and that it would only impact the day 0 and 7 samples, this quantitative bias was deemed insignificant for the purposes of this study. Overall, these large datasets demonstrate a remarkably low level of biological variability across all 10 sample groups, in addition to illustrating the quantitative precision that can be achieved with LRE qPCR.

## Competing interests

The authors have no non-financial, financial or patent related competing interests.

## Authors’ contribution

KK, SC, BB and JM designed the experimental approach, KK and CO collected the buds and conducted the SE induction, SC and BB conducted the microarray analysis, DS and RGR conducted the qPCR gene expression analysis, RGR wrote the manuscript with contributions from KK, SC, BB and JM. All authors read and approved the final manuscript.

## Supplementary Material

Additional file 1 Summary of the microarray analysis. Click here for file

Additional file 2 DHN1 expression ratios, EST and amino acid sequence alignments. Click here for file

Additional file 3 Amino acid sequence alignments of PgPrx52, PgPrx21 and PgcsINV1. Click here for file

Additional file 4 EST alignments and primer locations for the candidate and reference genes. Click here for file

Additional file 5 qPCR primers for reference and candidate gene expression analysis. Click here for file

Additional file 6 LRE Analyzer database files. Click here for file

Additional file 7 Excel summary of the LRE qPCR gene expression analysis. Click here for file

## References

[B1] Preil W Laimer M, Rücker W, Wien W Plant tissue culture, 100 years since Gottlieb Haberlandt Micropropagation of ornamental plants 2003 New York: Springer 115 133

[B2] Nehra NS Becwar MR Rottmann WH Pearson L Chowdhury K Chang S Dayton Wilde H Kodrzycki RJ Zhang C Gause KC Parks DW Hinchee M Vitro Cell Dev Biol-Plant Forest biotechnology: innovative methods, emerging opportunities 2005 41 701 717

[B3] Klimaszewska K Trontin JF Becwar M Devillard C Park YS Lelu-Walter MA Recent progress in somatic embryogenesis of four Pinus spp Tree For Sci Biotechnol 2007 1 11 25

[B4] Bonga JM Klimaszewska K Aderkas P Recalcitrance in clonal propagation, in particular of conifers Plant Cell Tissue and Organ Cult 2010 100 241 254 10.1007/s11240-009-9647-2

[B5] Park SY Klimaszewska K Park JY Mansfield SD Lodgepole pine: the first evidence of seed-based somatic embryogenesis and the expression of embryogenesis marker genes in shoot bud cultures of adult trees Tree Physiol 2010 30 1469 1478 10.1093/treephys/tpq081 20935320

[B6] Verdeil JL Alemanno L Niemenak N Tranbarger TJ Pluripotent versus totipotent plant stem cells: dependence versus autonomy? Trends Plant Sci 2007 12 245 252 10.1016/j.tplants.2007.04.002 17499544

[B7] Zhang H Ogas J An epigenetic perspective on developmental regulation of seed genes Mol Plant 2009 2 610 627 10.1093/mp/ssp027 19825643

[B8] Grafi G Florentin A Ransbotyn V Morgenstern Y The stem cell state in plant development and in response to stress Front Plant Sci 2011 2 1 10 22645540 10.3389/fpls.2011.00053 PMC3355748

[B9] Klimaszewska K Overton C Stewart D Rutledge RG Initiation of somatic embryos and regeneration of plants from primordial shoots of 10-year-old somatic white spruce and expression profiles of 11 genes followed during the tissue culture process Planta 2011 233 635 647 10.1007/s00425-010-1325-4 21136075

[B10] Rigault P Boyle B Lepage P Cooke JEK Bousquet J MacKay JJ A white spruce gene catalog for conifer genome analyses Plant Physiol 2011 157 14 28 10.1104/pp.111.179663 21730200 PMC3165865

[B11] MacKay JJ Dean JFD Plomion C Peterson DG Cánovas FM Pavy N Ingvarsson PK Savolainen O Guevara MA Fluch S Vinceti B Abarca D Díaz-Sala C Cervera MT Towards decoding the conifer giga-genome Plant Mol Biol 2012 80 6 555 569 10.1007/s11103-012-9961-7 22960864

[B12] Raherison ES Rigault P Caron S Poulin PL Boyle B Verta JP Giguère I Bomal C Bohlmann J MacKay JJ Transcriptome profiling in conifers and the PiceaGenExpress database show patterns of diversification within gene families and interspecific conservation in vascular gene expression BMC Genomics 2012 13 434 10.1186/1471-2164-13-434 22931377 PMC3534630

[B13] Yakovlev IA Asante DKA Gunnar C Jouni F Olavi P Dehydrins expression related to timing of bud burst in Norway spruce late flushing family Planta 2008 228 459 472 10.1007/s00425-008-0750-0 18493789

[B14] Petersen TN Brunak S Von Heijne G Nielsen H SignalP 4.0: discriminating signal peptides from transmembrane regions Nat Meth 2011 8 785 786 10.1038/nmeth.1701 21959131

[B15] Silverstein KT Moskal W Wu HC Underwood B Graham M Town CD VandenBosch K Small cysteine-rich peptides resembling antimicrobial peptides have been under-predicted in plants Plant J 2007 51 262 280 10.1111/j.1365-313X.2007.03136.x 17565583

[B16] Rutledge RG Stewart D A kinetic-based sigmoidal model for the polymerase chain reaction and its application to high-capacity absolute quantitative real-time PCR BMC Biotechnol 2008 8 47 10.1186/1472-6750-8-47 18466619 PMC2397388

[B17] Rutledge RG Stewart D Assessing the performance capabilities of LRE-based assays for absolute quantitative real-time PCR PLoS ONE 2010 5 e9731 10.1371/journal.pone.0009731 20305810 PMC2840021

[B18] Rutledge RG A Java program for LRE-based real-time qPCR that enables large-scale absolute quantification PLoS ONE 2011 6 e17636 10.1371/journal.pone.0017636 21407812 PMC3047581

[B19] Vandesompele J Kubista M Pfaffl M Logan J, Edwards K, Saunders NB Real-time PCR: current technology and applications Reference gene validation software for improved normalization 2009 4 Norfolk, UK: Caister Academic Press 47 64

[B20] Schmittgen TD Livak KJ Analyzing real-time PCR data by the comparative CT method Nat Protocols 2008 3 1101 1108 10.1038/nprot.2008.73 18546601

[B21] Huggett JF Dheda K Bustin SA Real-time RT-PCR normalisation; strategies and considerations Genes Immun 2005 6 279 284 10.1038/sj.gene.6364190 15815687

[B22] Gutierrez L Mauriat M Guénin S Pelloux J Lefebvre JF Louvet R Rusterucci C Moritz T Guerineau F Bellini C Van Wuytswinkel O The lack of a systematic validation of reference genes: a serious pitfall undervalued in reverse transcription-polymerase chain reaction (RT-PCR) analysis in plants Plant Biotechnol J 2008 6 609 618 10.1111/j.1467-7652.2008.00346.x 18433420

[B23] Lamb C Dixon RA The oxidative burst in plant disease resistance Annu Rev Plant Physiol Plant Mol Biol 1997 48 251 275 10.1146/annurev.arplant.48.1.251 15012264

[B24] Alvarez M Pennell RI Meijer P-j Ishikawa A Dixon RA Lamb C Reactive oxygen intermediates mediate a systemic signal network in the establishment of plant immunity Cell 1998 92 773 784 10.1016/S0092-8674(00)81405-1 9529253

[B25] Marino D Dunand C Puppo A Pauly N A burst of plant NADPH oxidases Trends Plant Sci 2012 17 9 15 10.1016/j.tplants.2011.10.001 22037416

[B26] Cheong YH Chang HS Gupta R Wang X Zhu T Luan S Transcriptional profiling reveals novel interactions between wounding, pathogen, abiotic stress, and hormonal responses in Arabidopsis Plant Physiol 2002 129 661 677 10.1104/pp.002857 12068110 PMC161692

[B27] Torres MA Jones JDG Dangl JL Reactive oxygen species signaling in response to pathogens Plant Physiol 2006 141 373 378 10.1104/pp.106.079467 16760490 PMC1475467

[B28] Pauwels L Inzé D Goossens A Jasmonate-inducible gene: what does it mean? Trends Plant Sci 2009 14 87 91 10.1016/j.tplants.2008.11.005 19162528

[B29] Chaouch S Queval G Noctor G AtRbohF is a crucial modulator of defence-associated metabolism and a key actor in the interplay between intracellular oxidative stress and pathogenesis responses in Arabidopsis Plant J 2012 69 613 627 10.1111/j.1365-313X.2011.04816.x 21985584

[B30] Robert-Seilaniantz A Grant MR Jones JDG Hormone crosstalk in plant disease and defense: more than just jasmonate-salicylate antagonism Ann Rev Phytopathol 2011 49 317 343 10.1146/annurev-phyto-073009-114447 21663438

[B31] Kazan K Manners JM JAZ repressors and the orchestration of phytohormone crosstalk Trends Plant Sci 2012 17 22 31 10.1016/j.tplants.2011.10.006 22112386

[B32] Thaler JS Humphrey PT Whiteman NK Evolution of jasmonate and salicylate signal crosstalk Trends Plant Sci 2012 17 260 270 10.1016/j.tplants.2012.02.010 22498450

[B33] Apel K Hirt H Reactive oxygen species: metabolism, oxidative stress, and signal transduction Ann Rev Plant Biol 2004 55 373 399 10.1146/annurev.arplant.55.031903.141701 15377225

[B34] Passardi F Cosio C Penel C Dunand C Peroxidases have more functions than a Swiss army knife Plant Cell Rep 2005 24 255 265 10.1007/s00299-005-0972-6 15856234

[B35] Almagro L Gómez Ros LV Belchi-Navarro S Bru R Ros Barceló A Pedreño M Class III peroxidases in plant defence reactions J Exp Bot 2009 60 377 390 10.1093/jxb/ern277 19073963

[B36] Cosio C Dunand C Specific functions of individual class III peroxidase genes J Exp Bot 2009 60 391 408 10.1093/jxb/ern318 19088338

[B37] Doehlemann G Linde KVD Aßmann D Schwammbach D Mohanty A Jackson D Kahmann R Pep1, a secreted effector protein of Ustilago maydis, is required for successful invasion of plant cells PLoS Pathog 2009 5 e1000290 10.1371/journal.ppat.1000290 19197359 PMC2631132

[B38] Hemetsberger C Herrberger C Zechmann B Hillmer M Doehlemann G The Ustilago maydis effector Pep1 suppresses plant immunity by inhibition of host peroxidase activity PLoS Pathog 2012 8 e1002684 10.1371/journal.ppat.1002684 22589719 PMC3349748

[B39] Kjaersgård IV Jespersen HM Rasmussen SK Welinder KG Sequence and RT-PCR expression analysis of two peroxidases from Arabidopsis thaliana belonging to a novel evolutionary branch of plant peroxidases Plant Mol Biol 1997 33 699 708 10.1023/A:1005707813801 9132061

[B40] Chassot C Nawrath C Métraux JP Cuticular defects lead to full immunity to a major plant pathogen Plant J 2007 49 972 980 10.1111/j.1365-313X.2006.03017.x 17257167

[B41] Potters G Horemans N Jansen MAK The cellular redox state in plant stress biology–a charging concept Plant Physiol Biochem 2010 48 292 300 10.1016/j.plaphy.2009.12.007 20137959

[B42] Mittler R Vanderauwera S Suzuki N Miller G Tognetti VB Vandepoele K Gollery M Shulaev V Van Breusegem F ROS signaling: the new wave? Trends Plant Sci 2011 16 300 309 10.1016/j.tplants.2011.03.007 21482172

[B43] Foyer CH Noctor G Ascorbate and glutathione: the heart of the redox hub Plant Physiol 2011 155 2 18 10.1104/pp.110.167569 21205630 PMC3075780

[B44] Hanin M Brini F Ebel C Toda Y Takeda S Masmoudi K Plant dehydrins and stress tolerance: versatile proteins for complex mechanisms Plant Signal Behav 2011 6 1503 1509 10.4161/psb.6.10.17088 21897131 PMC3256378

[B45] Roitsch T Balibrea ME Hofmann M Proels R Sinha AK Extracellular invertase: key metabolic enzyme and PR protein J Exp Bot 2003 54 513 524 10.1093/jxb/erg050 12508062

[B46] Roitsch T González MC Function and regulation of plant invertases: sweet sensations Trends Plant Sci 2004 9 606 613 10.1016/j.tplants.2004.10.009 15564128

[B47] Fotopoulos V Plant invertases: structure, function and regulation J Biol Res 2005 4 127 137

[B48] Berger S Sinha AK Roitsch T Plant physiology meets phytopathology: plant primary metabolism and plant-pathogen interactions J Exp Bot 2007 58 4019 4026 10.1093/jxb/erm298 18182420

[B49] Bolton MD Primary metabolism and plant defense–fuel for the fire Mol Plant-Microbe Interact 2009 22 487 497 10.1094/MPMI-22-5-0487 19348567

[B50] Essmann J Schmitz-Thom I Schön H Sonnewald S Weis E Scharte J RNA interference-mediated repression of cell wall invertase impairs defense in source leaves of tobacco Plant Physiol 2008 147 1288 1299 10.1104/pp.108.121418 18502974 PMC2442523

[B51] Van Loon LC Rep M Pieterse CMJ Significance of inducible defense-related proteins in infected plants Annu Rev Phytopathol 2006 44 135 162 10.1146/annurev.phyto.44.070505.143425 16602946

[B52] Chou HM Bundock N Rolfe SA Scholes JD Infection of Arabidopsis thaliana leaves with Albugo candida (white blister rust) causes a reprogramming of host metabolism Mol Plant Pathol 2000 1 99 113 10.1046/j.1364-3703.2000.00013.x 20572957

[B53] Fotopoulos V Gilbert MJ Pittman JK Marvier AC Buchanan AJ Sauer N Hall JL Williams LE The monosaccharide transporter gene, AtSTP4, and the cell-wall invertase, Atbetafruct1, are induced in Arabidopsis during infection with the fungal biotroph Erysiphe cichoracearum Plant Physiol 2003 132 821 829 10.1104/pp.103.021428 12805612 PMC167022

[B54] Bonfig KB Schreiber U Gabler A Roitsch T Berger S Infection with virulent and avirulent P. syringae strains differentially affects photosynthesis and sink metabolism in Arabidopsis leaves Planta 2006 225 1 12 10.1007/s00425-006-0303-3 16807755

[B55] Ditt RF Kerr KF De Figueiredo P Delrow J Comai L Nester EW The Arabidopsis thaliana transcriptome in response to Agrobacterium tumefaciens Mol Plant-Microbe Interact 2006 19 665 681 10.1094/MPMI-19-0665 16776300

[B56] Quilliam RS Swarbrick PJ Scholes JD Rolfe SA Imaging photosynthesis in wounded leaves of Arabidopsis thaliana J Exp Bot 2006 57 55 69 16339783 10.1093/jxb/erj039

[B57] Rawlings ND Tolle DP Barrett AJ Evolutionary families of peptidase inhibitors Biochem J 2004 378 705 716 10.1042/BJ20031825 14705960 PMC1224039

[B58] Rawlings ND Barrett AJ Bateman A MEROPS: the database of proteolytic enzymes, their substrates and inhibitors Nucleic Acids Res 2012 40 D343 D350 10.1093/nar/gkr987 22086950 PMC3245014

[B59] Silverstein KAT Graham MA Paape TD VandenBosch KA Genome organization of more than 300 defensin-like genes in Arabidopsis Plant Physiol 2005 138 600 610 10.1104/pp.105.060079 15955924 PMC1150381

[B60] Hartl M Giri AP Kaur H Baldwin IT The multiple functions of plant serine protease inhibitors: defense against herbivores and beyond Plant Signal Behav 2011 6 1009 1011 10.4161/psb.6.7.15504 22004998 PMC3257781

[B61] Morán-Diez E Rubio B Domínguez S Hermosa R Monte E Nicolás C Transcriptomic response of Arabidopsis thaliana after 24 h incubation with the biocontrol fungus Trichoderma harzianum J Plant Physiol 2012 169 614 620 10.1016/j.jplph.2011.12.016 22317785

[B62] Denoux C Galletti R Mammarella N Gopalan S Werck D De Lorenzo G Ferrari S Ausubel FM Dewdney J Activation of defense response pathways by OGs and Flg22 elicitors in Arabidopsis seedlings Mol Plant 2008 1 423 445 10.1093/mp/ssn019 19825551 PMC2954645

[B63] Prins A Mukubi JM Pellny TK Verrier PJ Beyene G Lopes MS Emami K Treumann A Lelarge-Trouverie C Noctor G Kunert KJ Kerchev P Foyer CH Acclimation to high CO2 in maize is related to water status and dependent on leaf rank Plant Cell Environ 2011 34 314 331 10.1111/j.1365-3040.2010.02245.x 21054434

[B64] Luhua S Ciftci-Yilmaz S Harper J Cushman J Mittler R Enhanced tolerance to oxidative stress in transgenic Arabidopsis plants expressing proteins of unknown function Plant Physiol 2008 148 280 292 10.1104/pp.108.124875 18614705 PMC2528079

[B65] Van Hulten M Pelser M Van Loon LC Pieterse CMJ Ton J Costs and benefits of priming for defense in Arabidopsis Proc Natl Acad Sci USA 2006 103 5602 5607 10.1073/pnas.0510213103 16565218 PMC1459400

[B66] Canet JV Dobón A Ibáñez F Perales L Tornero P Resistance and biomass in Arabidopsis: a new model for salicylic acid perception Plant Biotechnol J 2010 8 126 141 10.1111/j.1467-7652.2009.00468.x 20040060

[B67] Pauwels L Morreel K De Witte E Lammertyn F Van Montagu M Boerjan W Inzé D Goossens A Mapping methyl jasmonate-mediated transcriptional reprogramming of metabolism and cell cycle progression in cultured Arabidopsis cells Proc Natl Acad Sci U S A 2008 105 1380 1385 10.1073/pnas.0711203105 18216250 PMC2234147

[B68] Marsoni M Bracale M Espen L Prinsi B Negri AS Vannini C Proteomic analysis of somatic embryogenesis in Vitis vinifera Plant Cell Rep 2008 27 347 356 10.1007/s00299-007-0438-0 17874111

[B69] Zhang J Ma H Chen S Ji M Perl A Kovacs L Chen S Stress response proteins’ differential expression in embryogenic and non-embryogenic callus of Vitis vinifera L. cv. Cabernet Sauvignon–A proteomic approach Plant Sci 2009 177 103 113 10.1016/j.plantsci.2009.04.003

[B70] Sharifi G Ebrahimzadeh H Ghareyazie B Gharechahi J Vatankhah E Identification of differentially accumulated proteins associated with embryogenic and non-embryogenic calli in saffron (Crocus sativus L.) Proteome Sci 2012 10 3 10.1186/1477-5956-10-3 22243837 PMC3349542

[B71] Correia S Vinhas R Manadas B Lourenço AS Veríssimo P Canhoto JM Comparative proteomic analysis of auxin-induced embryogenic and nonembryogenic tissues of the solanaceous tree Cyphomandra betacea (Tamarillo) J Proteome Res 2012 11 1666 1675 10.1021/pr200856w 22309186

[B72] Rose RJ Nolan KE Vitro Cell Dev Biol Invited review: Genetic regulation of somatic embryogenesis with particular reference to Arabidopsis thaliana and Medicago truncatula 2006 42 473 481

[B73] Almeida AM Parreira JR Santos R Duque AS Francisco R Tomé DFA Ricardo CP Coelho AV Fevereiro P A proteomics study of the induction of somatic embryogenesis in Medicago truncatula using 2DE and MALDI-TOF/TOF Physiol Plant 2012 146 236 249 10.1111/j.1399-3054.2012.01633.x 22497501

[B74] Pavy N Boyle B Nelson C Paule C Giguère I Caron S Parsons LS Dallaire N Bedon F Bérubé H Cooke J MacKay JJ Identification of conserved core xylem gene sets: conifer cDNA microarray development, transcript profiling and computational analyses New Phytol 2008 180 766 786 10.1111/j.1469-8137.2008.02615.x 18811621

[B75] MacKenzie DJ McLean MA Mukerji S Green M Improved RNA extraction from woody plants for the detection of viral pathogens by reverse transcription-polymerase chain reaction Plant Dis 1997 81 222 226 10.1094/PDIS.1997.81.2.222 30870901

[B76] R Core Team A language and environment for statistical computing http://www.R-project.org

[B77] FlexArray A statistical data analysis software for gene expression microarrays McGill University and Genome Quebec Innovation Centre http://www.gqinnovationcenter.com/services/bioinformatics/flexarray/index.aspx?l=e

[B78] Rutledge RG Stewart D Critical evaluation of methods used to determine amplification efficiency refutes the exponential character of real-time PCR BMC Mol Biol 2008 9 96 10.1186/1471-2199-9-96 18973660 PMC2587475

[B79] LRE qPCR Websitehttp://sites.google.com/site/lreqpcr/

[B80] LRE qPCR Analyzerhttp://code.google.com/p/lreqpcr/

[B81] Friedmann M Ralph SG Aeschliman D Zhuang J Ritland K Ellis BE Bohlmann J Douglas CJ Microarray gene expression profiling of developmental transitions in Sitka spruce (Picea sitchensis) apical shoots J Exp Bot 2007 58 593 614 17220514 10.1093/jxb/erl246

[B82] Czechowski T Stitt M Altmann T Genome-wide identification and testing of superior reference genes for transcript normalization in Arabidopsis Plant Physiol 2005 139 5 17 10.1104/pp.105.063743 16166256 PMC1203353

[B83] Hruz T Wyss M Docquier M Pfaffl MW Masanetz S Borghi L Verbrugghe P Kalaydjieva L Bleuler S Laule O Descombes P Gruissem W Zimmermann P RefGenes: identification of reliable and condition specific reference genes for RT-qPCR data normalization BMC Genomics 2011 12 156 10.1186/1471-2164-12-156 21418615 PMC3072958

