# Supplementary material for: Potential link between biotic defense activation and recalcitrance to induction of somatic embryogenesis in shoot primordia from adult trees of white spruce (Picea glauca)
Source: BMC Plant Biol. 2013 Aug 12;13:116. doi: 10.1186/1471-2229-13-116 (PMC3765131; doi:10.1186/1471-2229-13-116)
Supplement: Additional file 2 — DHN1 expression ratios, EST and amino acid sequence alignments. [file 1471-2229-13-116-S2.doc]

# Microarray analysis of DHN1

Nine of the top 30 differentially expressed genes within the G6 explants were found to target genes encoding for dehydrin, which were subsequently found to be part of a small gene family encoding for three variants of an unusual conifer-specific dehydrin called DHN1.

| **Putative ID** | | **G6 / G12 Day 0** | | **G6 / G12 Day 7** | | **G6 Day 7/0** | | **G12 Day 7/0** | | **Probe** |
| --- | --- | --- | --- | --- | --- | --- | --- | --- | --- | --- |
| Dehydrin | | 1.00 | 8.15 | | 8.75 | | 1.07 | | 15981 | |
| Dehydrin | | 1.01 | 7.27 | | 38.56 | | 5.36 | | 25506 | |
| Dehydrin | | 1.00 | 6.77 | | 8.26 | | 1.22 | | 18408 | |
| Dehydrin | | 1.00 | 5.00 | | 27.63 | | 5.53 | | 27873 | |
| Dehydrin | | 1.00 | 5.25 | | 16.20 | | 3.09 | | 09510 | |
| Dehydrin | | 1.00 | 4.87 | | 9.86 | | 2.02 | | 09769 | |
| Dehydrin | | 1.32 | 3.86 | | 29.30 | | 10.04 | | 27485 | |
| Dehydrin | | 1.00 | 3.36 | | 6.46 | | 1.78 | | 27435 | |
| Dehydrin | | 1.07 | 3.10 | | 7.84 | | 2.71 | | 10902 | |
| Average: | 1.04 | | 5.29 | | 16.98 | | 3.65 | |  | |

# DHN1 EST sequence alignments with the 9 probes targeted to this gene family

All P. glauca ESTs

**09510: GATATATCTATCTGCTTTTGAAATTGAAATTAGTACCATCATGGCTGGAAATCAGGAGTGCCAGGACCGC**

**27873:** **TTGATATATCTATCTGCTTTTGAAATTGAAAGTAGTACCGCCATGGCTGGAAATCAGGAGTGCCAGGACC**

[BT117865](http://www.ncbi.nlm.nih.gov/entrez/query.fcgi?cmd=Retrieve&db=Nucleotide&list_uids=270150979&dopt=GenBank&RID=YS8242YF112&log$=nuclalign&blast_rank=0) 1 ATCTTATCTGCAATTTTACGAAGTTGATTGTCTCAGTTTGTTGATTGATATATCTATCTGCTTTTGAAATTGAAAATAGTACCGCC**ATG**GCTGGAAATCAGGATTGCCAGGACCGCGGC 119

[CO252099](http://www.ncbi.nlm.nih.gov/nucest/CO252099) 688 .........................A.......................T........C.........G............... 605

**27435: TTGATATATCTATCTTCTTTTGACATT-------ATACAATCATGGCTGCAAATCAGGAGTGTCAGGACCGCGGCCT**

**09769:** [**TTGATATATCTATTCGCTTTTGACATT-------ATACTATCATGGCTGCAAATCAGGAGTGTCAGGACCGCGGCCT**](http://www.ncbi.nlm.nih.gov/nucest/EX419599.1)

**15981(SS): TTGATATATCTATTCGCTTTTGACATT-------ATACTATCATGGCTGCAAATCAGGAGTGTCAGGACCGCGGCCT**

[BT115236](http://www.ncbi.nlm.nih.gov/entrez/query.fcgi?cmd=Retrieve&db=Nucleotide&list_uids=270148284&dopt=GenBank&RID=1D1AYKTG112&log$=nuclalign&blast_rank=0) 22 ..........................T.................................T......C...-------A...TAT........C.........G..T............ 133

[BT115255](http://www.ncbi.nlm.nih.gov/entrez/query.fcgi?cmd=Retrieve&db=Nucleotide&list_uids=270148303&dopt=GenBank&RID=1D1AYKTG112&log$=nuclalign&blast_rank=0) 6 ..........................T.................................T......C...-------A...TAT........C.........G..T............ 117

[BT117770](http://www.ncbi.nlm.nih.gov/entrez/query.fcgi?cmd=Retrieve&db=Nucleotide&list_uids=270150884&dopt=GenBank&RID=1D1AYKTG112&log$=nuclalign&blast_rank=0) 1 ..........................T................................T.......C...-------A...AAT........C.........G..T............ 112

[BT115468](http://www.ncbi.nlm.nih.gov/entrez/query.fcgi?cmd=Retrieve&db=Nucleotide&list_uids=270148516&dopt=GenBank&RID=1D1AYKTG112&log$=nuclalign&blast_rank=0) 6 ..........................T................................CT..............T.......AT..................G............... 124

[BT116481](http://www.ncbi.nlm.nih.gov/entrez/query.fcgi?cmd=Retrieve&db=Nucleotide&list_uids=270149595&dopt=GenBank&RID=1D1AYKTG112&log$=nuclalign&blast_rank=0) 6 ..........................T........C.......................................T.......AT..................G............... 124

[EX378761](http://www.ncbi.nlm.nih.gov/entrez/query.fcgi?cmd=Retrieve&db=Nucleotide&list_uids=157577533&dopt=GenBank&RID=1D1AYKTG112&log$=nuclalign&blast_rank=0) 5 ....................T.....A................G..........T..............T.......AT........C.........G..T............ 117

[BT114790](http://www.ncbi.nlm.nih.gov/entrez/query.fcgi?cmd=Retrieve&db=Nucleotide&list_uids=270147838&dopt=GenBank&RID=1D1AYKTG112&log$=nuclalign&blast_rank=0) 6 ..........................T................................CT..............T.......AT..................G............... 124

[BT117952](http://www.ncbi.nlm.nih.gov/entrez/query.fcgi?cmd=Retrieve&db=Nucleotide&list_uids=270151066&dopt=GenBank&RID=1D1AYKTG112&log$=nuclalign&blast_rank=0) 12 ...........................................................................G...........................G............... 130

[BT117559](http://www.ncbi.nlm.nih.gov/entrez/query.fcgi?cmd=Retrieve&db=Nucleotide&list_uids=270150673&dopt=GenBank&RID=1D1AYKTG112&log$=nuclalign&blast_rank=0) 10 .......................................................................................................G............... 128

[BT117865](http://www.ncbi.nlm.nih.gov/entrez/query.fcgi?cmd=Retrieve&db=Nucleotide&list_uids=270150979&dopt=GenBank&RID=YS8242YF112&log$=nuclalign&blast_rank=0) 120 CTCTTCGGCAAGAAGGACGAGGGAAGGCAGGATGATGAAATGATGCAGAATCAGGCTACTCGTCCAAATCAAAATCCAACTCAAAAGGCAGGGCTCGTCGATAAAGTGAAAGAGAAGCTC 239

[CO252099](http://www.ncbi.nlm.nih.gov/nucest/CO252099) 604 ....G..............................................................................G.....G.............................. 485

[BT115236](http://www.ncbi.nlm.nih.gov/entrez/query.fcgi?cmd=Retrieve&db=Nucleotide&list_uids=270148284&dopt=GenBank&RID=1D1AYKTG112&log$=nuclalign&blast_rank=0) 134 ..A.G.............................------------.....................................G.....G.............................. 241

[BT115255](http://www.ncbi.nlm.nih.gov/entrez/query.fcgi?cmd=Retrieve&db=Nucleotide&list_uids=270148303&dopt=GenBank&RID=1D1AYKTG112&log$=nuclalign&blast_rank=0) 118 ..A.G.............................------------.....................................G.G...G.............................. 225

[BT117770](http://www.ncbi.nlm.nih.gov/entrez/query.fcgi?cmd=Retrieve&db=Nucleotide&list_uids=270150884&dopt=GenBank&RID=1D1AYKTG112&log$=nuclalign&blast_rank=0) 113 ..A.G.............................------------.....................................G.....G.............................. 220

[BT115468](http://www.ncbi.nlm.nih.gov/entrez/query.fcgi?cmd=Retrieve&db=Nucleotide&list_uids=270148516&dopt=GenBank&RID=1D1AYKTG112&log$=nuclalign&blast_rank=0) 125 ..................................------------.....................................G.....G.............................. 232

[BT116481](http://www.ncbi.nlm.nih.gov/entrez/query.fcgi?cmd=Retrieve&db=Nucleotide&list_uids=270149595&dopt=GenBank&RID=1D1AYKTG112&log$=nuclalign&blast_rank=0) 125 ..................................------------.....................................G.....G.................A............ 232

[EX378761](http://www.ncbi.nlm.nih.gov/entrez/query.fcgi?cmd=Retrieve&db=Nucleotide&list_uids=157577533&dopt=GenBank&RID=1D1AYKTG112&log$=nuclalign&blast_rank=0) 118 ..A.G.............................------------.....................................G.....G.............................A 225

[BT114790](http://www.ncbi.nlm.nih.gov/entrez/query.fcgi?cmd=Retrieve&db=Nucleotide&list_uids=270147838&dopt=GenBank&RID=1D1AYKTG112&log$=nuclalign&blast_rank=0) 125 ..................................------------.....................................G.....G.............................. 232

[BT117952](http://www.ncbi.nlm.nih.gov/entrez/query.fcgi?cmd=Retrieve&db=Nucleotide&list_uids=270151066&dopt=GenBank&RID=1D1AYKTG112&log$=nuclalign&blast_rank=0) 131 ....G..........A...........................------------------------................G.....G.............................. 226

[BT117559](http://www.ncbi.nlm.nih.gov/entrez/query.fcgi?cmd=Retrieve&db=Nucleotide&list_uids=270150673&dopt=GenBank&RID=1D1AYKTG112&log$=nuclalign&blast_rank=0) 129 ....G..........A...........................------------------------................G.....G.............................. 224

[BT117865](http://www.ncbi.nlm.nih.gov/entrez/query.fcgi?cmd=Retrieve&db=Nucleotide&list_uids=270150979&dopt=GenBank&RID=YS8242YF112&log$=nuclalign&blast_rank=0) 240 CCTGGAGGCCAGACTAAGACTCAGAGTCAGACTGCTCAGTGTAACCCAACTGAGAAGCCCGGAATGGCGGATAAAATCAAAGAGAAGCTTCCCGGAGGCCGTAACAAGGAA**TGA**CCTCTC 359

[CO252099](http://www.ncbi.nlm.nih.gov/nucest/CO252099) 484 .......................A................................................................................................ 365

[BT115236](http://www.ncbi.nlm.nih.gov/entrez/query.fcgi?cmd=Retrieve&db=Nucleotide&list_uids=270148284&dopt=GenBank&RID=1D1AYKTG112&log$=nuclalign&blast_rank=0) 242 ........G............................................................................................................... 361

[BT115255](http://www.ncbi.nlm.nih.gov/entrez/query.fcgi?cmd=Retrieve&db=Nucleotide&list_uids=270148303&dopt=GenBank&RID=1D1AYKTG112&log$=nuclalign&blast_rank=0) 226 ........G............................................................................................................... 345

[BT117770](http://www.ncbi.nlm.nih.gov/entrez/query.fcgi?cmd=Retrieve&db=Nucleotide&list_uids=270150884&dopt=GenBank&RID=1D1AYKTG112&log$=nuclalign&blast_rank=0) 221 ........................................................................................................................ 340

[BT115468](http://www.ncbi.nlm.nih.gov/entrez/query.fcgi?cmd=Retrieve&db=Nucleotide&list_uids=270148516&dopt=GenBank&RID=1D1AYKTG112&log$=nuclalign&blast_rank=0) 233 ........G............................................................................................................... 352

[BT116481](http://www.ncbi.nlm.nih.gov/entrez/query.fcgi?cmd=Retrieve&db=Nucleotide&list_uids=270149595&dopt=GenBank&RID=1D1AYKTG112&log$=nuclalign&blast_rank=0) 233 ........................................................................................................................ 352

[EX378761](http://www.ncbi.nlm.nih.gov/entrez/query.fcgi?cmd=Retrieve&db=Nucleotide&list_uids=157577533&dopt=GenBank&RID=1D1AYKTG112&log$=nuclalign&blast_rank=0) 226 ........G............................................................................................................... 345

[BT114790](http://www.ncbi.nlm.nih.gov/entrez/query.fcgi?cmd=Retrieve&db=Nucleotide&list_uids=270147838&dopt=GenBank&RID=1D1AYKTG112&log$=nuclalign&blast_rank=0) 233 ........G............................................................................................................... 352

[BT117952](http://www.ncbi.nlm.nih.gov/entrez/query.fcgi?cmd=Retrieve&db=Nucleotide&list_uids=270151066&dopt=GenBank&RID=1D1AYKTG112&log$=nuclalign&blast_rank=0) 227 ........G............................................................................................................... 346

[BT117559](http://www.ncbi.nlm.nih.gov/entrez/query.fcgi?cmd=Retrieve&db=Nucleotide&list_uids=270150673&dopt=GenBank&RID=1D1AYKTG112&log$=nuclalign&blast_rank=0) 225 ........................................................................................................................ 344

**18408:** AGAAATGAATGCCATCCGATTGGCAAGCCATGCTCA

[BT117865](http://www.ncbi.nlm.nih.gov/entrez/query.fcgi?cmd=Retrieve&db=Nucleotide&list_uids=270150979&dopt=GenBank&RID=YS8242YF112&log$=nuclalign&blast_rank=0) 360 GCTTTTCCCTGGAGCATCCACCGATAATAGTGATAATCATATGAAAGTAATAGTGTGTGTTAGAACTTA-GAGTAGAGAATA-------AGATATGAATGCCATCCGATCCGCAAGCCATGCTTA 476

[CO252099](http://www.ncbi.nlm.nih.gov/nucest/CO252099) 364 ........-........................................G...................-............-------.................................... 249

[BT115236](http://www.ncbi.nlm.nih.gov/entrez/query.fcgi?cmd=Retrieve&db=Nucleotide&list_uids=270148284&dopt=GenBank&RID=1D1AYKTG112&log$=nuclalign&blast_rank=0) 362 .....................................................................-............-------...A................TG............C. 478

[BT115255](http://www.ncbi.nlm.nih.gov/entrez/query.fcgi?cmd=Retrieve&db=Nucleotide&list_uids=270148303&dopt=GenBank&RID=1D1AYKTG112&log$=nuclalign&blast_rank=0) 346 .................-----------------------.............................-............TAGAATA....................TG.T..........C. 446

[BT117770](http://www.ncbi.nlm.nih.gov/entrez/query.fcgi?cmd=Retrieve&db=Nucleotide&list_uids=270150884&dopt=GenBank&RID=1D1AYKTG112&log$=nuclalign&blast_rank=0) 341 .....................................................................A............-------.................................... 458

[BT115468](http://www.ncbi.nlm.nih.gov/entrez/query.fcgi?cmd=Retrieve&db=Nucleotide&list_uids=270148516&dopt=GenBank&RID=1D1AYKTG112&log$=nuclalign&blast_rank=0) 353 .....................................................................-............-------.................................... 469

[BT116481](http://www.ncbi.nlm.nih.gov/entrez/query.fcgi?cmd=Retrieve&db=Nucleotide&list_uids=270149595&dopt=GenBank&RID=1D1AYKTG112&log$=nuclalign&blast_rank=0) 353 .....................................................................A............-------.................................... 470

[BT114790](http://www.ncbi.nlm.nih.gov/entrez/query.fcgi?cmd=Retrieve&db=Nucleotide&list_uids=270147838&dopt=GenBank&RID=1D1AYKTG112&log$=nuclalign&blast_rank=0) 353 ..........---------..................................................-.G..........-------.................................... 462

**25506**ATGACCTCTCGCTTTTCCCTGGAGCAT-----------------------ATGAAAGTAATAGTGTGTGTTAGAACTTA-GAGTAGAGAATA-------AG

[EX378761](http://www.ncbi.nlm.nih.gov/entrez/query.fcgi?cmd=Retrieve&db=Nucleotide&list_uids=157577533&dopt=GenBank&RID=1D1AYKTG112&log$=nuclalign&blast_rank=0) 346 .................-----------------------.............................-............-------.................................... 439

[BT117952](http://www.ncbi.nlm.nih.gov/entrez/query.fcgi?cmd=Retrieve&db=Nucleotide&list_uids=270151066&dopt=GenBank&RID=1D1AYKTG112&log$=nuclalign&blast_rank=0) 347 .....................................................................-............-------.................................... 463

[BT117559](http://www.ncbi.nlm.nih.gov/entrez/query.fcgi?cmd=Retrieve&db=Nucleotide&list_uids=270150673&dopt=GenBank&RID=1D1AYKTG112&log$=nuclalign&blast_rank=0) 345 .................-----------------------..........................C..-..A..T......TAGAATA.....................G.T..........C. 445

GTATAGTATTT-----------------TTTTGTTCGGAGGAGATTTGTAG

[BT117865](http://www.ncbi.nlm.nih.gov/entrez/query.fcgi?cmd=Retrieve&db=Nucleotide&list_uids=270150979&dopt=GenBank&RID=YS8242YF112&log$=nuclalign&blast_rank=0) 477 GAATAGTA--------------------TTTGGCTGGGAGGAGATTTGTACTTCTGCTGTGATCGTGGGTTTCAGCTTTCGTTTTTCTAGTATGAATAGAAACCAAAGAAGAATATA 573

[BT117559](http://www.ncbi.nlm.nih.gov/entrez/query.fcgi?cmd=Retrieve&db=Nucleotide&list_uids=270150673&dopt=GenBank&RID=1D1AYKTG112&log$=nuclalign&blast_rank=0) 446 .T......--------------------.....T.CT..........--------------------------------------.T.......T........T.T.T..G------

**27485:** AGAATAGTATTTGGATGCTTAGAATAGTATTTGGCTGGGAGGAGATTTGTACTTCTGCTGTGATCGTGGG

[CO252099](http://www.ncbi.nlm.nih.gov/nucest/CO252099) 248 ........TTTGGATGCTTAGAATAGTA..............................................A............................T............. 132

[BT115236](http://www.ncbi.nlm.nih.gov/entrez/query.fcgi?cmd=Retrieve&db=Nucleotide&list_uids=270148284&dopt=GenBank&RID=1D1AYKTG112&log$=nuclalign&blast_rank=0) 479 .T......TTT-----------------...T.T.C..............G........................................G...........T............. 578

[BT115255](http://www.ncbi.nlm.nih.gov/entrez/query.fcgi?cmd=Retrieve&db=Nucleotide&list_uids=270148303&dopt=GenBank&RID=1D1AYKTG112&log$=nuclalign&blast_rank=0) 447 .T......TTT-----------------.....T.C..........................................................T........T.--------.... 538

[BT117770](http://www.ncbi.nlm.nih.gov/entrez/query.fcgi?cmd=Retrieve&db=Nucleotide&list_uids=270150884&dopt=GenBank&RID=1D1AYKTG112&log$=nuclalign&blast_rank=0) 459 ........--------------------..A...............................................................T........T.--------.... 547

[BT115468](http://www.ncbi.nlm.nih.gov/entrez/query.fcgi?cmd=Retrieve&db=Nucleotide&list_uids=270148516&dopt=GenBank&RID=1D1AYKTG112&log$=nuclalign&blast_rank=0) 470 .....A..--------------------...............................................A..................T........T............. 566

[BT116481](http://www.ncbi.nlm.nih.gov/entrez/query.fcgi?cmd=Retrieve&db=Nucleotide&list_uids=270149595&dopt=GenBank&RID=1D1AYKTG112&log$=nuclalign&blast_rank=0) 471 ........--------------------..................................................................T......G.T.--------.... 559

[EX378761](http://www.ncbi.nlm.nih.gov/entrez/query.fcgi?cmd=Retrieve&db=Nucleotide&list_uids=157577533&dopt=GenBank&RID=1D1AYKTG112&log$=nuclalign&blast_rank=0) 440 ........--------------------.............G........................................................A....T..C.......... 536

[BT114790](http://www.ncbi.nlm.nih.gov/entrez/query.fcgi?cmd=Retrieve&db=Nucleotide&list_uids=270147838&dopt=GenBank&RID=1D1AYKTG112&log$=nuclalign&blast_rank=0) 463 .....A..--------------------..................................................................T........T.--------.... 551

[BT117952](http://www.ncbi.nlm.nih.gov/entrez/query.fcgi?cmd=Retrieve&db=Nucleotide&list_uids=270151066&dopt=GenBank&RID=1D1AYKTG112&log$=nuclalign&blast_rank=0) 464 ........--------------------..................................................................T........T.--------.... 552

**10902**: GGACGTCCATGCCTTACAAGCTTGCT------------GTCAAGAGCTAAATGTATGGGTATCTATTAATGTGGAAAAATAC

[BT117865](http://www.ncbi.nlm.nih.gov/entrez/query.fcgi?cmd=Retrieve&db=Nucleotide&list_uids=270150979&dopt=GenBank&RID=YS8242YF112&log$=nuclalign&blast_rank=0) 574 TCATGATGTCCATGCCTTACAAGCTTGCT------------GTCAAGAGCTAAATGTATGGGCATGCATTAATGTGGAAAAATACAGTAATACTTCATATACCTTATTTTCATAATCTT 680

[CO252099](http://www.ncbi.nlm.nih.gov/nucest/CO252099) 131 ...G..C......................TACAAGCTTGCT........T...............C.......T.........................................AAAA 13 polyA

[BT115236](http://www.ncbi.nlm.nih.gov/entrez/query.fcgi?cmd=Retrieve&db=Nucleotide&list_uids=270148284&dopt=GenBank&RID=1D1AYKTG112&log$=nuclalign&blast_rank=0) 579 .........T..............C....------------.............................................................................. 685

[BT115255](http://www.ncbi.nlm.nih.gov/entrez/query.fcgi?cmd=Retrieve&db=Nucleotide&list_uids=270148303&dopt=GenBank&RID=1D1AYKTG112&log$=nuclalign&blast_rank=0) 539 --.G..C......................------------.............................................................................. 643

[BT117770](http://www.ncbi.nlm.nih.gov/entrez/query.fcgi?cmd=Retrieve&db=Nucleotide&list_uids=270150884&dopt=GenBank&RID=1D1AYKTG112&log$=nuclalign&blast_rank=0) 548 --.G..C.....................A------------..G...C.................C...C......................A..T................ 645

# Amino acid sequent alignments for DHN1

PgDHN1a (88 aa)

[CO252099](http://www.ncbi.nlm.nih.gov/entrez/query.fcgi?cmd=Retrieve&db=Nucleotide&list_uids=49132240&dopt=GenBank&RID=1D1AYKTG112&log$=nuclalign&blast_rank=0) MAANQECQDRGLCGKKDEGRQDDEMMQNQATRPNQNPTQKAGLVDKVKEKLPGGQTKTQSQTAQCNPTEKPGMADKIKEKLPGGRNKE Very large 5' UTR

[BT117865](http://www.ncbi.nlm.nih.gov/entrez/query.fcgi?cmd=Retrieve&db=Nucleotide&list_uids=270150979&dopt=GenBank&RID=YS8242YF112&log$=nuclalign&blast_rank=0) MA**G**NQ**D**CQDRGL**F**GKKDEGRQDDEMMQNQATRPNQNPTQKAGLVDKVKEKLPGGQTKTQSQTAQCNPTEKPGMADKIKEKLPGGRNKE

PgDHN1b (84 aa)

[BT115236](http://www.ncbi.nlm.nih.gov/entrez/query.fcgi?cmd=Retrieve&db=Nucleotide&list_uids=270148284&dopt=GenBank&RID=1D1AYKTG112&log$=nuclalign&blast_rank=0) MAANQECQDRGLCGKKDEGRQDENQATRPNQNPTQKAGLVDKVKEKLPGGQTKTQSQTAQCNPTEKPGMADKIKEKLPGGRNKE

[BT115255](http://www.ncbi.nlm.nih.gov/entrez/query.fcgi?cmd=Retrieve&db=Nucleotide&list_uids=270148303&dopt=GenBank&RID=1D1AYKTG112&log$=nuclalign&blast_rank=0) MAANQECQDRGLCGKKDEGRQDENQATRPNQNPTQRAGLVDKVKEKLPGGQTKTQSQTAQCNPTEKPGMADKIKEKLPGGRNKE

[BT117770](http://www.ncbi.nlm.nih.gov/entrez/query.fcgi?cmd=Retrieve&db=Nucleotide&list_uids=270150884&dopt=GenBank&RID=1D1AYKTG112&log$=nuclalign&blast_rank=0) MAANQECQDRGLCGKKDEGRQDENQATRPNQNPTQKAGLVDKVKEKLPGGQTKTQSQTAQCNPTEKPGMADKIKEKLPGGRNKE

[BT115468](http://www.ncbi.nlm.nih.gov/entrez/query.fcgi?cmd=Retrieve&db=Nucleotide&list_uids=270148516&dopt=GenBank&RID=1D1AYKTG112&log$=nuclalign&blast_rank=0) MA**G**NQECQDRGL**F**GKKDEGRQDENQATRPNQNPTQKAGLVDKVKEKLPGGQTKTQSQTAQCNPTEKPGMADKIKEKLPGGRNKE

[BT116481](http://www.ncbi.nlm.nih.gov/entrez/query.fcgi?cmd=Retrieve&db=Nucleotide&list_uids=270149595&dopt=GenBank&RID=1D1AYKTG112&log$=nuclalign&blast_rank=0) MA**G**NQECQDRGL**F**GKKDEGRQDENQATRPNQNPTQKAGLVDKVKEKLPGGQTKTQSQTAQCNPTEKPGMADKIKEKLPGGRNKE

[EX378761](http://www.ncbi.nlm.nih.gov/entrez/query.fcgi?cmd=Retrieve&db=Nucleotide&list_uids=157577533&dopt=GenBank&RID=1D1AYKTG112&log$=nuclalign&blast_rank=0) MYLSVFEIEISTIMAANQECQDRGLCGKKDEGRQDENQATRPNQNPTQKAGLVDKVKEKLPGGQTKTQSQTAQCNPTEKPGMADKIKEKLPGGRNKE 101 aa

[BT114790](http://www.ncbi.nlm.nih.gov/entrez/query.fcgi?cmd=Retrieve&db=Nucleotide&list_uids=270147838&dopt=GenBank&RID=1D1AYKTG112&log$=nuclalign&blast_rank=0) MA**G**NQECQDRGL**F**GKKDEGRQDENQATRPNQNPTQKAGLVDKVKEKLPGGQTKTQSQTAQCNPTEKPGMADKIKEKLPGGRNKE

PgDHN1c (80 aa)

[BT117952](http://www.ncbi.nlm.nih.gov/entrez/query.fcgi?cmd=Retrieve&db=Nucleotide&list_uids=270151066&dopt=GenBank&RID=1D1AYKTG112&log$=nuclalign&blast_rank=0) MAGNQECQDRGLCGKKNEGRQDDEMNQNPTQKAGLVDKVKEKLPGGQTKTQSQTAQCNPTEKPGMADKIKEKLPGGRNKE

[BT117559](http://www.ncbi.nlm.nih.gov/entrez/query.fcgi?cmd=Retrieve&db=Nucleotide&list_uids=270150673&dopt=GenBank&RID=1D1AYKTG112&log$=nuclalign&blast_rank=0) MAGNQECQDRGLCGKKNEGRQDDEMNQNPTQKAGLVDKVKEKLPGGQTKTQSQTAQCNPTEKPGMADKIKEKLPGGRNKE

Two repetitive carboxyl segments containing a dehydrin K-segment are in bold, along with three cysteines not commonly found in dehydrins shown in red

PgDHN1a 1 MAGNQD**C**QDRGLFGKKDEGRQDDEMMQNQATRPNQNPT**QKAGLVDKVKEKLPG**GQTKTQSQTAQ**C**NPT**EKPGMADKIKEKLPG**GRNKE 88

PgDHN1b 1 ..A..E**.**.....**C**.........E----.....................................**.**....................... 84

PgDHN1c 1 .....E**.**.....**C**...N........--------...............................**.**....................... 80

All contain two well-conserved Lys-rich domains (K-segments) with the consensus motif EKKGIMDKIKEKLPG.

Other Conifers

PgDHN1a 1 MAGNQDCQDRGL**F**GKKDEGRQDDEMMQNQATRPNQNPTQKAGLVDKVKEKLPGGQTKTQSQTAQCNPTEKPGMADKIKEKLPGGRNKE 88

[Sitka ABK21645](http://www.ncbi.nlm.nih.gov/entrez/query.fcgi?cmd=Retrieve&db=Protein&list_uids=116780352&dopt=GenPept&RID=1FHFCGN4114&log$=protalign&blast_rank=0) 1 .....E.................................................................................. 88

[Sitka ADE76499](http://www.ncbi.nlm.nih.gov/entrez/query.fcgi?cmd=Retrieve&db=Protein&list_uids=294461884&dopt=GenPept&RID=1FHFCGN4114&log$=protalign&blast_rank=0) 1 .....E......C........................................................................... 88

[Sitka ABK25374](http://www.ncbi.nlm.nih.gov/entrez/query.fcgi?cmd=Retrieve&db=Protein&list_uids=116789764&dopt=GenPept&RID=1FHFCGN4114&log$=protalign&blast_rank=0) 1 ..A..E......C........................................................................... 88

PgDHN1b 1 MAANQE**C**QDRGL**C**GKKDEGRQDENQATRPNQNPTQKAGLVDKVKEKLPGGQTKTQSQTAQ**C**NPTEKPGMADKIKEKLPGGRNKE 84

[Sitka ADE76208](http://www.ncbi.nlm.nih.gov/entrez/query.fcgi?cmd=Retrieve&db=Protein&list_uids=294461292&dopt=GenPept&RID=1FJA36K6114&log$=protalign&blast_rank=0) 1 .................................................................................... 84

[Norway AY961924](http://www.ncbi.nlm.nih.gov/nuccore/AY961924) 1 .....D..................................................................V........... 84

PgDHN1c 1 MAGNQECQDRGLCGKK**N**EGRQDDEMNQNPTQKAGLVDKVKEKLPGGQTKTQSQTAQCNPTEKPGMADKIKEKLPGGRNKE 80

[Sitka CO215948](http://www.ncbi.nlm.nih.gov/nucest/49038263) 565 ................D............................................................... 326
