# Supplementary material for: Potential link between biotic defense activation and recalcitrance to induction of somatic embryogenesis in shoot primordia from adult trees of white spruce (Picea glauca)
Source: BMC Plant Biol. 2013 Aug 12;13:116. doi: 10.1186/1471-2229-13-116 (PMC3765131; doi:10.1186/1471-2229-13-116)
Supplement: Additional file 3 — Amino acid sequence alignments of PgPrx52, PgPrx21 and PgcsINV1. [file 1471-2229-13-116-S3.doc]

**Figure S1 Candidate gene amino acid sequences aligned to the most similar Arabidopsis homolog.** **(A)** Carboxyl terminal region of the class III apoplastic peroxidase AtPrx52 (At5G05340). **(B)** Carboxyl terminal region of the class III apoplastic peroxidase AtPrx21 (also called ATP2a/b; At2G37130). **(C)** Cell wall invertase AtcwINV1, (also called AtβFruct1; At3G13790). Conserved substitutions are highlighted in yellow.
