# Supplementary material for: Potential link between biotic defense activation and recalcitrance to induction of somatic embryogenesis in shoot primordia from adult trees of white spruce (Picea glauca)
Source: BMC Plant Biol. 2013 Aug 12;13:116. doi: 10.1186/1471-2229-13-116 (PMC3765131; doi:10.1186/1471-2229-13-116)
Supplement: Additional file 4 — EST alignments and primer locations for the candidate and reference genes. [file 1471-2229-13-116-S4.doc]

# EST alignments that include the microarray probes (red) and qPCR primers (arrows) for the G6UP candidate genes

## P. glauca DNH1 (see Additional file 2 for probe locations)

[BT117865](http://www.ncbi.nlm.nih.gov/entrez/query.fcgi?cmd=Retrieve&db=Nucleotide&list_uids=270150979&dopt=GenBank&RID=YS8242YF112&log$=nuclalign&blast_rank=0) 1 ATCTTATCTGCAATTTTACGAAGTTGATTGTCTCAGTTTGTTGATTGATATATCTATCTGCTTTTGAAATTGAAAATAGTACCGCC**ATG**GCTGGAAATCAGGATTGCCAGGACCGCGGC 119

[CO252099](http://www.ncbi.nlm.nih.gov/nucest/CO252099) 688 .........................A.......................T........C.........G............... 605

[BT115236](http://www.ncbi.nlm.nih.gov/entrez/query.fcgi?cmd=Retrieve&db=Nucleotide&list_uids=270148284&dopt=GenBank&RID=1D1AYKTG112&log$=nuclalign&blast_rank=0) 22 ..........................T.................................T......C...-------A...TAT........C.........G..T............ 133

[BT115255](http://www.ncbi.nlm.nih.gov/entrez/query.fcgi?cmd=Retrieve&db=Nucleotide&list_uids=270148303&dopt=GenBank&RID=1D1AYKTG112&log$=nuclalign&blast_rank=0) 6 ..........................T.................................T......C...-------A...TAT........C.........G..T............ 117

[BT117770](http://www.ncbi.nlm.nih.gov/entrez/query.fcgi?cmd=Retrieve&db=Nucleotide&list_uids=270150884&dopt=GenBank&RID=1D1AYKTG112&log$=nuclalign&blast_rank=0) 1 ..........................T................................T.......C...-------A...AAT........C.........G..T............ 112

[BT115468](http://www.ncbi.nlm.nih.gov/entrez/query.fcgi?cmd=Retrieve&db=Nucleotide&list_uids=270148516&dopt=GenBank&RID=1D1AYKTG112&log$=nuclalign&blast_rank=0) 6 ..........................T................................CT..............T.......AT..................G............... 124

[BT116481](http://www.ncbi.nlm.nih.gov/entrez/query.fcgi?cmd=Retrieve&db=Nucleotide&list_uids=270149595&dopt=GenBank&RID=1D1AYKTG112&log$=nuclalign&blast_rank=0) 6 ..........................T........C.......................................T.......AT..................G............... 124

[EX378761](http://www.ncbi.nlm.nih.gov/entrez/query.fcgi?cmd=Retrieve&db=Nucleotide&list_uids=157577533&dopt=GenBank&RID=1D1AYKTG112&log$=nuclalign&blast_rank=0) 5 ....................T.....A................G..........T..............T.......AT........C.........G..T............ 117

[BT114790](http://www.ncbi.nlm.nih.gov/entrez/query.fcgi?cmd=Retrieve&db=Nucleotide&list_uids=270147838&dopt=GenBank&RID=1D1AYKTG112&log$=nuclalign&blast_rank=0) 6 ..........................T................................CT..............T.......AT..................G............... 124

[BT117952](http://www.ncbi.nlm.nih.gov/entrez/query.fcgi?cmd=Retrieve&db=Nucleotide&list_uids=270151066&dopt=GenBank&RID=1D1AYKTG112&log$=nuclalign&blast_rank=0) 12 ...........................................................................G...........................G............... 130

[BT117559](http://www.ncbi.nlm.nih.gov/entrez/query.fcgi?cmd=Retrieve&db=Nucleotide&list_uids=270150673&dopt=GenBank&RID=1D1AYKTG112&log$=nuclalign&blast_rank=0) 10 .......................................................................................................G............... 128

**DHN1 uF2**======================>

**DHN1 aF1**============================>

[BT117865](http://www.ncbi.nlm.nih.gov/entrez/query.fcgi?cmd=Retrieve&db=Nucleotide&list_uids=270150979&dopt=GenBank&RID=YS8242YF112&log$=nuclalign&blast_rank=0) 120 CTCTTCGGCA**AGAAGGACGAGGGAAGGCAGGAT**GATGAAATGATGCAGAATCAGGCTACTCGTCCAAATCAAAATCCAACTCAAAAGGCAGGGCTCGTCGATAAAGTGAAAGAGAAGCTC 239

[CO252099](http://www.ncbi.nlm.nih.gov/nucest/CO252099) 604 ....G..............................................................................G.....G.............................. 485

**DHN1 bF1**===================------------=====>

[BT115236](http://www.ncbi.nlm.nih.gov/entrez/query.fcgi?cmd=Retrieve&db=Nucleotide&list_uids=270148284&dopt=GenBank&RID=1D1AYKTG112&log$=nuclalign&blast_rank=0) 134 ..A.G.............................------------.....................................G.....G.............................. 241

[BT115255](http://www.ncbi.nlm.nih.gov/entrez/query.fcgi?cmd=Retrieve&db=Nucleotide&list_uids=270148303&dopt=GenBank&RID=1D1AYKTG112&log$=nuclalign&blast_rank=0) 118 ..A.G.............................------------.....................................G.G...G.............................. 225

[BT117770](http://www.ncbi.nlm.nih.gov/entrez/query.fcgi?cmd=Retrieve&db=Nucleotide&list_uids=270150884&dopt=GenBank&RID=1D1AYKTG112&log$=nuclalign&blast_rank=0) 113 ..A.G.............................------------.....................................G.....G.............................. 220

[BT115468](http://www.ncbi.nlm.nih.gov/entrez/query.fcgi?cmd=Retrieve&db=Nucleotide&list_uids=270148516&dopt=GenBank&RID=1D1AYKTG112&log$=nuclalign&blast_rank=0) 125 ..................................------------.....................................G.....G.............................. 232

[BT116481](http://www.ncbi.nlm.nih.gov/entrez/query.fcgi?cmd=Retrieve&db=Nucleotide&list_uids=270149595&dopt=GenBank&RID=1D1AYKTG112&log$=nuclalign&blast_rank=0) 125 ..................................------------.....................................G.....G.................A............ 232

[EX378761](http://www.ncbi.nlm.nih.gov/entrez/query.fcgi?cmd=Retrieve&db=Nucleotide&list_uids=157577533&dopt=GenBank&RID=1D1AYKTG112&log$=nuclalign&blast_rank=0) 118 ..A.G.............................------------.....................................G.....G.............................A 225

[BT114790](http://www.ncbi.nlm.nih.gov/entrez/query.fcgi?cmd=Retrieve&db=Nucleotide&list_uids=270147838&dopt=GenBank&RID=1D1AYKTG112&log$=nuclalign&blast_rank=0) 125 ..................................------------.....................................G.....G.............................. 232

**DHN1 cF1**=======================------------------------====>

[BT117952](http://www.ncbi.nlm.nih.gov/entrez/query.fcgi?cmd=Retrieve&db=Nucleotide&list_uids=270151066&dopt=GenBank&RID=1D1AYKTG112&log$=nuclalign&blast_rank=0) 131 ....G..........A...........................------------------------................G.....G.............................. 226

[BT117559](http://www.ncbi.nlm.nih.gov/entrez/query.fcgi?cmd=Retrieve&db=Nucleotide&list_uids=270150673&dopt=GenBank&RID=1D1AYKTG112&log$=nuclalign&blast_rank=0) 129 ....G..........A...........................------------------------................G.....G.............................. 224

**uF1======================...**

<=========================**DHN1 uR3 <**=======================**DHN1 uR2 <=====================...DHN1 uR4**

[BT117865](http://www.ncbi.nlm.nih.gov/entrez/query.fcgi?cmd=Retrieve&db=Nucleotide&list_uids=270150979&dopt=GenBank&RID=YS8242YF112&log$=nuclalign&blast_rank=0) 240 CCTGGAGGCCAGACTAAGACTCAGAGTCAGACTGCTCAGTGTAACCCAACTGAGAAGCCCGGAATGGCGGATAAAATCAAAGAGAAGCTTCCCGGAGGCCGTAACAAGGAA**TGA**CCTCTC 359

[CO252099](http://www.ncbi.nlm.nih.gov/nucest/CO252099) 484 .......................A................................................................................................ 365

[BT115236](http://www.ncbi.nlm.nih.gov/entrez/query.fcgi?cmd=Retrieve&db=Nucleotide&list_uids=270148284&dopt=GenBank&RID=1D1AYKTG112&log$=nuclalign&blast_rank=0) 242 ........G............................................................................................................... 361

[BT115255](http://www.ncbi.nlm.nih.gov/entrez/query.fcgi?cmd=Retrieve&db=Nucleotide&list_uids=270148303&dopt=GenBank&RID=1D1AYKTG112&log$=nuclalign&blast_rank=0) 226 ........G............................................................................................................... 345

[BT117770](http://www.ncbi.nlm.nih.gov/entrez/query.fcgi?cmd=Retrieve&db=Nucleotide&list_uids=270150884&dopt=GenBank&RID=1D1AYKTG112&log$=nuclalign&blast_rank=0) 221 ........................................................................................................................ 340

[BT115468](http://www.ncbi.nlm.nih.gov/entrez/query.fcgi?cmd=Retrieve&db=Nucleotide&list_uids=270148516&dopt=GenBank&RID=1D1AYKTG112&log$=nuclalign&blast_rank=0) 233 ........G............................................................................................................... 352

[BT116481](http://www.ncbi.nlm.nih.gov/entrez/query.fcgi?cmd=Retrieve&db=Nucleotide&list_uids=270149595&dopt=GenBank&RID=1D1AYKTG112&log$=nuclalign&blast_rank=0) 233 ........................................................................................................................ 352

[EX378761](http://www.ncbi.nlm.nih.gov/entrez/query.fcgi?cmd=Retrieve&db=Nucleotide&list_uids=157577533&dopt=GenBank&RID=1D1AYKTG112&log$=nuclalign&blast_rank=0) 226 ........G............................................................................................................... 345

[BT114790](http://www.ncbi.nlm.nih.gov/entrez/query.fcgi?cmd=Retrieve&db=Nucleotide&list_uids=270147838&dopt=GenBank&RID=1D1AYKTG112&log$=nuclalign&blast_rank=0) 233 ........G............................................................................................................... 352

[BT117952](http://www.ncbi.nlm.nih.gov/entrez/query.fcgi?cmd=Retrieve&db=Nucleotide&list_uids=270151066&dopt=GenBank&RID=1D1AYKTG112&log$=nuclalign&blast_rank=0) 227 ........G............................................................................................................... 346

[BT117559](http://www.ncbi.nlm.nih.gov/entrez/query.fcgi?cmd=Retrieve&db=Nucleotide&list_uids=270150673&dopt=GenBank&RID=1D1AYKTG112&log$=nuclalign&blast_rank=0) 225 ........................................................................................................................ 344

**uF1...===>**

**...====DHN1 uR4**

[BT117865](http://www.ncbi.nlm.nih.gov/entrez/query.fcgi?cmd=Retrieve&db=Nucleotide&list_uids=270150979&dopt=GenBank&RID=YS8242YF112&log$=nuclalign&blast_rank=0) 360 GCTTTTCCCTGGAGCATCCACCGATAATAGTGATAATCATATGAAAGTAATAGTGTGTGTTAGAACTTA-GAGTAGAGAATA-------AGATATGAATGCCATCCGATCCGCAAGCCATGCTTA 476

[CO252099](http://www.ncbi.nlm.nih.gov/nucest/CO252099) 364 ........-........................................G...................-............-------.................................... 249

[BT115236](http://www.ncbi.nlm.nih.gov/entrez/query.fcgi?cmd=Retrieve&db=Nucleotide&list_uids=270148284&dopt=GenBank&RID=1D1AYKTG112&log$=nuclalign&blast_rank=0) 362 .....................................................................-............-------...A................TG............C. 478

[BT115255](http://www.ncbi.nlm.nih.gov/entrez/query.fcgi?cmd=Retrieve&db=Nucleotide&list_uids=270148303&dopt=GenBank&RID=1D1AYKTG112&log$=nuclalign&blast_rank=0) 346 .................-----------------------.............................-............TAGAATA....................TG.T..........C. 446

[BT117770](http://www.ncbi.nlm.nih.gov/entrez/query.fcgi?cmd=Retrieve&db=Nucleotide&list_uids=270150884&dopt=GenBank&RID=1D1AYKTG112&log$=nuclalign&blast_rank=0) 341 .....................................................................A............-------.................................... 458

[BT115468](http://www.ncbi.nlm.nih.gov/entrez/query.fcgi?cmd=Retrieve&db=Nucleotide&list_uids=270148516&dopt=GenBank&RID=1D1AYKTG112&log$=nuclalign&blast_rank=0) 353 .....................................................................-............-------.................................... 469

[BT116481](http://www.ncbi.nlm.nih.gov/entrez/query.fcgi?cmd=Retrieve&db=Nucleotide&list_uids=270149595&dopt=GenBank&RID=1D1AYKTG112&log$=nuclalign&blast_rank=0) 353 .....................................................................A............-------.................................... 470

[BT114790](http://www.ncbi.nlm.nih.gov/entrez/query.fcgi?cmd=Retrieve&db=Nucleotide&list_uids=270147838&dopt=GenBank&RID=1D1AYKTG112&log$=nuclalign&blast_rank=0) 353 ..........---------..................................................-.G..........-------.................................... 462

[EX378761](http://www.ncbi.nlm.nih.gov/entrez/query.fcgi?cmd=Retrieve&db=Nucleotide&list_uids=157577533&dopt=GenBank&RID=1D1AYKTG112&log$=nuclalign&blast_rank=0) 346 .................-----------------------.............................-............-------.................................... 439

[BT117952](http://www.ncbi.nlm.nih.gov/entrez/query.fcgi?cmd=Retrieve&db=Nucleotide&list_uids=270151066&dopt=GenBank&RID=1D1AYKTG112&log$=nuclalign&blast_rank=0) 347 .....................................................................-............-------.................................... 463

[BT117559](http://www.ncbi.nlm.nih.gov/entrez/query.fcgi?cmd=Retrieve&db=Nucleotide&list_uids=270150673&dopt=GenBank&RID=1D1AYKTG112&log$=nuclalign&blast_rank=0) 345 .................-----------------------..........................C..-..A..T......TAGAATA.....................G.T..........C. 445

[BT117865](http://www.ncbi.nlm.nih.gov/entrez/query.fcgi?cmd=Retrieve&db=Nucleotide&list_uids=270150979&dopt=GenBank&RID=YS8242YF112&log$=nuclalign&blast_rank=0) 477 GAATAGTA--------------------TTTGGCTGGGAGGAGATTTGTACTTCTGCTGTGATCGTGGGTTTCAGCTTTCGTTTTTCTAGTATGAATAGAAACCAAAGAAGAATATA 573

[CO252099](http://www.ncbi.nlm.nih.gov/nucest/CO252099) 248 ........TTTGGATGCTTAGAATAGTA.............................................A............................T............. 132

[BT115236](http://www.ncbi.nlm.nih.gov/entrez/query.fcgi?cmd=Retrieve&db=Nucleotide&list_uids=270148284&dopt=GenBank&RID=1D1AYKTG112&log$=nuclalign&blast_rank=0) 479 .T......TTT-----------------...T.T.C..............G........................................G...........T............. 578

[BT115255](http://www.ncbi.nlm.nih.gov/entrez/query.fcgi?cmd=Retrieve&db=Nucleotide&list_uids=270148303&dopt=GenBank&RID=1D1AYKTG112&log$=nuclalign&blast_rank=0) 447 .T......TTT-----------------.....T.C..........................................................T........T.--------.... 538

[BT117770](http://www.ncbi.nlm.nih.gov/entrez/query.fcgi?cmd=Retrieve&db=Nucleotide&list_uids=270150884&dopt=GenBank&RID=1D1AYKTG112&log$=nuclalign&blast_rank=0) 459 ........--------------------..A...............................................................T........T.--------.... 547

[BT115468](http://www.ncbi.nlm.nih.gov/entrez/query.fcgi?cmd=Retrieve&db=Nucleotide&list_uids=270148516&dopt=GenBank&RID=1D1AYKTG112&log$=nuclalign&blast_rank=0) 470 .....A..--------------------...............................................A..................T........T............. 566

[BT116481](http://www.ncbi.nlm.nih.gov/entrez/query.fcgi?cmd=Retrieve&db=Nucleotide&list_uids=270149595&dopt=GenBank&RID=1D1AYKTG112&log$=nuclalign&blast_rank=0) 471 ........--------------------..................................................................T......G.T.--------.... 559

[EX378761](http://www.ncbi.nlm.nih.gov/entrez/query.fcgi?cmd=Retrieve&db=Nucleotide&list_uids=157577533&dopt=GenBank&RID=1D1AYKTG112&log$=nuclalign&blast_rank=0) 440 ........--------------------.............G........................................................A....T..C.......... 536

[BT114790](http://www.ncbi.nlm.nih.gov/entrez/query.fcgi?cmd=Retrieve&db=Nucleotide&list_uids=270147838&dopt=GenBank&RID=1D1AYKTG112&log$=nuclalign&blast_rank=0) 463 .....A..--------------------..................................................................T........T.--------.... 551

[BT117952](http://www.ncbi.nlm.nih.gov/entrez/query.fcgi?cmd=Retrieve&db=Nucleotide&list_uids=270151066&dopt=GenBank&RID=1D1AYKTG112&log$=nuclalign&blast_rank=0) 464 ........--------------------..................................................................T........T.--------.... 552

[BT117559](http://www.ncbi.nlm.nih.gov/entrez/query.fcgi?cmd=Retrieve&db=Nucleotide&list_uids=270150673&dopt=GenBank&RID=1D1AYKTG112&log$=nuclalign&blast_rank=0) 446 .T......--------------------.....T.CT..........--------------------------------------.T.......T........T.T.T..G------

[BT117865](http://www.ncbi.nlm.nih.gov/entrez/query.fcgi?cmd=Retrieve&db=Nucleotide&list_uids=270150979&dopt=GenBank&RID=YS8242YF112&log$=nuclalign&blast_rank=0) 574 TCATGATGTCCATGCCTTACAAGCTTGCT------------GTCAAGAGCTAAATGTATGGGCATGCATTAATGTGGAAAAATACAGTAATACTTCATATACCTTATTTTCATAATCTT 680

[CO252099](http://www.ncbi.nlm.nih.gov/nucest/CO252099) 131 ...G..C......................TACAAGCTTGCT........T...............C.......T.........................................AAAA 13 polyA

[BT115236](http://www.ncbi.nlm.nih.gov/entrez/query.fcgi?cmd=Retrieve&db=Nucleotide&list_uids=270148284&dopt=GenBank&RID=1D1AYKTG112&log$=nuclalign&blast_rank=0) 579 .........T..............C....------------.............................................................................. 685

[BT115255](http://www.ncbi.nlm.nih.gov/entrez/query.fcgi?cmd=Retrieve&db=Nucleotide&list_uids=270148303&dopt=GenBank&RID=1D1AYKTG112&log$=nuclalign&blast_rank=0) 539 --.G..C......................------------.............................................................................. 643

[BT117770](http://www.ncbi.nlm.nih.gov/entrez/query.fcgi?cmd=Retrieve&db=Nucleotide&list_uids=270150884&dopt=GenBank&RID=1D1AYKTG112&log$=nuclalign&blast_rank=0) 548 --.G..C.....................A------------..G...C.................C...C......................A..T................ 645

[BT115468](http://www.ncbi.nlm.nih.gov/entrez/query.fcgi?cmd=Retrieve&db=Nucleotide&list_uids=270148516&dopt=GenBank&RID=1D1AYKTG112&log$=nuclalign&blast_rank=0) 567 .A.G..C.....................A------------..G...C.................C...C......................A....................G..... 673

[BT116481](http://www.ncbi.nlm.nih.gov/entrez/query.fcgi?cmd=Retrieve&db=Nucleotide&list_uids=270149595&dopt=GenBank&RID=1D1AYKTG112&log$=nuclalign&blast_rank=0) 560 --.G..C......................------------..............................................C......................T........ 664

[EX378761](http://www.ncbi.nlm.nih.gov/entrez/query.fcgi?cmd=Retrieve&db=Nucleotide&list_uids=157577533&dopt=GenBank&RID=1D1AYKTG112&log$=nuclalign&blast_rank=0) 537 ...................... 558

[BT114790](http://www.ncbi.nlm.nih.gov/entrez/query.fcgi?cmd=Retrieve&db=Nucleotide&list_uids=270147838&dopt=GenBank&RID=1D1AYKTG112&log$=nuclalign&blast_rank=0) 552 --.G..C......................------------........................CT.........................A....................G..... 656

[BT117952](http://www.ncbi.nlm.nih.gov/entrez/query.fcgi?cmd=Retrieve&db=Nucleotide&list_uids=270151066&dopt=GenBank&RID=1D1AYKTG112&log$=nuclalign&blast_rank=0) 553 --.G..C.....................A------------..G...C.................C...C................ 624

[BT117559](http://www.ncbi.nlm.nih.gov/entrez/query.fcgi?cmd=Retrieve&db=Nucleotide&list_uids=270150673&dopt=GenBank&RID=1D1AYKTG112&log$=nuclalign&blast_rank=0) 498 .A.G..C......................------------.....................T..CT.........................A...................... 600

[BT117865](http://www.ncbi.nlm.nih.gov/entrez/query.fcgi?cmd=Retrieve&db=Nucleotide&list_uids=270150979&dopt=GenBank&RID=YS8242YF112&log$=nuclalign&blast_rank=0) 681 AAAAGAATAAATTTGT-TCATGGCTGAGTnaaaaaa 715

[CO252099](http://www.ncbi.nlm.nih.gov/nucest/CO252099)

[BT115236](http://www.ncbi.nlm.nih.gov/entrez/query.fcgi?cmd=Retrieve&db=Nucleotide&list_uids=270148284&dopt=GenBank&RID=1D1AYKTG112&log$=nuclalign&blast_rank=0) 686 ......G......... 701

[BT115255](http://www.ncbi.nlm.nih.gov/entrez/query.fcgi?cmd=Retrieve&db=Nucleotide&list_uids=270148303&dopt=GenBank&RID=1D1AYKTG112&log$=nuclalign&blast_rank=0) 644 ......G.........-.....TT.....-...... 677

[BT117770](http://www.ncbi.nlm.nih.gov/entrez/query.fcgi?cmd=Retrieve&db=Nucleotide&list_uids=270150884&dopt=GenBank&RID=1D1AYKTG112&log$=nuclalign&blast_rank=0)

[BT115468](http://www.ncbi.nlm.nih.gov/entrez/query.fcgi?cmd=Retrieve&db=Nucleotide&list_uids=270148516&dopt=GenBank&RID=1D1AYKTG112&log$=nuclalign&blast_rank=0) 674 ......G.......-.C........... 700

[BT116481](http://www.ncbi.nlm.nih.gov/entrez/query.fcgi?cmd=Retrieve&db=Nucleotide&list_uids=270149595&dopt=GenBank&RID=1D1AYKTG112&log$=nuclalign&blast_rank=0) 665 ......G.........-... 683

[EX378761](http://www.ncbi.nlm.nih.gov/entrez/query.fcgi?cmd=Retrieve&db=Nucleotide&list_uids=157577533&dopt=GenBank&RID=1D1AYKTG112&log$=nuclalign&blast_rank=0)

[BT114790](http://www.ncbi.nlm.nih.gov/entrez/query.fcgi?cmd=Retrieve&db=Nucleotide&list_uids=270147838&dopt=GenBank&RID=1D1AYKTG112&log$=nuclalign&blast_rank=0) 657 .......A... 667

## QT-repeat (7925)

CO228028 1 ACGAAGGATGCGAGAATCAGACTGGCCCGACTCAGTCTCAGTATGGCCCGACTCAGACTCAGTATGGCATCGGTCAGACCCAGACCCAGACCCAGACCCAGACTCAGACTCTGACTCAGG 120

BT100806 245 ACGAAGGATGCGATAATCAGACTGGCCCGACTCAGTCTCAGTATGGCCCGACTCAGACTCAGTATGGCATCGGTCAGACCCAGACCCAGACCCAGACCCAGACTCAGACTCTGACTCAGG 364

BT101037 13 ACGAAGGATGCGAGAATCAGACTGGCTCGACTCAGTGTCAGTATGGCCCGACTCAGACCCAGTATGGCATCGGTCAGACCCAGACCCAGACCCAGACCCAGACTCAGACTCTGACTCAGG 132

EF083127 256 ACGAAGGATGCGAGAATCAGACTGGCCCGACTCAGTCTCAGTATGGCCCGACTCAGACTCAGTATGGCATCGGTCAGACCCAGACCCAGACTCAGACCCAGACCCAGACTCTGACTC--- 372

BT070924 251 ACGAAGGATGCGAGAATCAGACTGGCCCGACTCAGTCTCAGTATGGCCCGACTCAGACTCAGTATGGCATCGGTCAGACCCAGACCCAGACTCAGACCCAGACCCAGACTCTGACTC--- 367

CO228028 121 GAAGCGAGGGCACGAATTcgactccggctccgactccgactccggctccggctccgaccccggctctggctcgggctccggctccggctccgactccgactccgactccggc------ 234

BT100806 365 GAAGCGAGGGCACGAATTCGACTCCGGCTCCGGCTCCGGCTCCGACTCCGGCTCTGGCTCGGGCTCGGGCTCCGGCTCCGTCTCCGGCTCCGACTCCGACTCCGGCTCCGGCTCCGGC 484

BT101037 133 GAAGCGAGAGCACGAATTCGACTCCGGCTCCGACTCCGGCTCCGGCTCCGGCTCCGACTCCGGCTCTGGCTCGGGCTCGGGCTCCGGCTCCGGCTCCGACTCCGACTCCGGC------ 246

EF083127 Sitka

BT070924 Sitka

**7925**: TCCGGCTCCGGGAAATTCTGGATGCCAGATTCAGAGTGGCGAGAGTCAATTTCAGAGTGGCATGGGTCAA

CO228028 235 tccggctccggGAAATTCTGGATGCCAGATTCAGAGTGGCGAGAGTCAATTTCAGAGTGGCATGGGTCAAACTCAGACTCAGACTCTGACTCAGACTCAAACTCTGACTTCGAGTCCGAATA 354

BT100806 485 TCCGGCTCCGGGAAATTCTGGATGCCAGATTCAGAGTGGCGAGAGTCAATTTCAGAGTGGCATGGGTCAAACTCAGACTCAGACTCTGACTCAGACTCAAACTCTGACTTCGAGTCCGAATA 604

BT101037 247 TCCGGCTCCGGGAAATTCTGGATGCCAGATTCAGAGTGGCGAGAGTCAATTTCAGAGTGGCATGGGTCAAACTCAGACTCAGACTCTGACCCAGACTCAAACTCTGACTTCGAGTCCGAATA 366

EF083127 493 --AGACTCAGGGAAAGTCTGGATGCCAGATTCAGAGTGGCGAGAGTCAATTTCAGAGTGGCATGGGTCAAACTCAGACTCAGACTCTGACTCAGACTCAAACTCTGACTTCGAGTCCGAATT 609

BT070924 488 --AGACTCAGGGAAAGTCTGGATGCCAGATTCAGAGTGGCGAGAGTCAATTTCAGAGTGGCATGGGTCAAACTCAGACTCAGACTCTGACTCAGACTCAAACTCTGACTTCGAGTCCGAATT 604

F1===========================> <====================...R2

F2=========================>

CO228028 355 CGACTACGAAAATTAAAGCAAACAATGGCATAGTCGTTGATGCAGAAATAATGGCATAATCATTGAAGAGTAATGTTTGT-----CTTAGAATATGTTTAGTATAATATTTGGTTCGGAG 469

BT100806 605 CGACTACGAAAATTAAAGCAAACAATGGCATAGTCGTTGATGCAGAAATAATGGCATAATCATTGAAGAGTAATGTTTGT-----CTTAGAATATGTTTAGTATAATATTTGGTTCGGAG 719

BT101037 367 CGACTACGAAAATTAAAGCAAACAATGGCATAGTCGTTGATGCAGAAATAATGGCATAATCATTGAAGAGTAATGTTTGT-----CTTAGAATATGTTTAGTATAATATTTGGTTCGGAG 481

EF083127 610 CGACTACGACAATTAAAGCAAACAATGGCATAGTCGTTGATGCAGAGATAAAGGCATAATCATTGAAGAGTAATGTTTGTTAGAACTTAGAATATGTTTAGTATAATATTTGGTTCGGAG 729

BT070924 605 CGACTACGACAATTAAAGCAAACAATGGCATAGTCGTTGATGCAGAGATAAAGGCATAATCATTGAAGAGTAATGTTTGTTAGAACTTAGAATATGTTTAGTATAATATTTGGTTCGGAG 724

...========R2 <=========================7925R1

CO228028 470 GAGATTTGTACTTTTGTTGTGACCGTGGATTATAGCTTTCGTTTTTCTAGTATGAACAGAAACTAAAGATGACTTACATGTAGTCTATATTGCTGGAAAGCTATTTTATTATTAAATAAC 589

BT100806 720 GAGATTTGTACTTTTGTTGTGACCGTGGATTATAGCTTTCGTTTTTCTAGTATGAACAGAAACTAAAGATGACTTACATGTAGTCTATATTGCTGGAAAGTTATTTTATTATTTAATAAC 839

BT101037 482 GAGATTTGTACTTTTGTTGTGACC 505

EF083127 730 GAGATTTGTACTTTTGTTGTGACCATGGATTATAGCTTTCGTTTTTCTAGTATGAATAGAAACTAAAGATGACTTACATGTAGTCTATATTGCTGGAAAGTTATTTTATTATTAAATAAC 849

BT070924 725 GAGATTTGTACTTTTGTTGTGACCATGGATTATAGCTTTCGTTTTTCTAGTATGAATAGAAACTAAAGATGACTTACATGTAGTCTATATTGCTGGAAAGTTATTTTATTATTAAATAAC 844

CO228028 590 AATTTTCACaaaaaaaaaaaaaa 612

BT100806 840 AATTTTCACAAAACTATAAAAAA 862

EF083127 850 AATTTTCACAAAA 862

BT070924 845 AATTTTCACAAAA 857

## PgPrx52 (708)

[BT101740](http://www.ncbi.nlm.nih.gov/nuccore/270134788) 1 CGAGGTGCGTGAATTTCAGAGCCCATATTTACAACGAAACCAACATAGACAGTACGTACTCCACTTCATTGCAGTCAAAGTGTCCGAGTACCGCAGGCTCCGGAGACAGCAACCTGTCGC 120

[CO254451](http://www.ncbi.nlm.nih.gov/entrez/query.fcgi?cmd=Retrieve&db=Nucleotide&list_uids=49137570&dopt=GenBank&RID=Z83S00BC112&log$=nuclalign&blast_rank=0) 561 ............................................................................................A........................... 442

[DR590294](http://www.ncbi.nlm.nih.gov/entrez/query.fcgi?cmd=Retrieve&db=Nucleotide&list_uids=70655037&dopt=GenBank&RID=Z83S00BC112&log$=nuclalign&blast_rank=0) 415 .................C..............................................T.... 347

[BT101740](http://www.ncbi.nlm.nih.gov/nuccore/270134788) 121 CACTGGATTATGTGACTCCCACTGCCTTCGACAAAAACTATTACAGCAATCTGAAAAGCAAAAAGGGACTTCTCCACTCCGATCAGGAACTCTTCAATGGAGGCTCCAC**TGA**TTCACAGG 240

[CO254451](http://www.ncbi.nlm.nih.gov/entrez/query.fcgi?cmd=Retrieve&db=Nucleotide&list_uids=49137570&dopt=GenBank&RID=Z83S00BC112&log$=nuclalign&blast_rank=0) 441 ...................................................................T.................................................... 322

[DR590294](http://www.ncbi.nlm.nih.gov/entrez/query.fcgi?cmd=Retrieve&db=Nucleotide&list_uids=70655037&dopt=GenBank&RID=Z83S00BC112&log$=nuclalign&blast_rank=0) 346 ........................................................................................................................ 227

**708F1**========================>

[BT101740](http://www.ncbi.nlm.nih.gov/nuccore/270134788) 241 TGACTACGTACGCCTCAAACCAGAATACCTTCTTCTCAGACTTTGCCGCAGCCATGGTTAAGATGGGAAATATCAAACCTCTTACAGGAACCAGCGGACAGATCCGAAATAATTGCAGGA 360

[CO254451](http://www.ncbi.nlm.nih.gov/entrez/query.fcgi?cmd=Retrieve&db=Nucleotide&list_uids=49137570&dopt=GenBank&RID=Z83S00BC112&log$=nuclalign&blast_rank=0) 321 ........................................................................................................................ 202

[CO252529](http://www.ncbi.nlm.nih.gov/entrez/query.fcgi?cmd=Retrieve&db=Nucleotide&list_uids=49133219&dopt=GenBank&RID=Z83S00BC112&log$=nuclalign&blast_rank=0) 305 302 ............................................................... 243

[DR590294](http://www.ncbi.nlm.nih.gov/entrez/query.fcgi?cmd=Retrieve&db=Nucleotide&list_uids=70655037&dopt=GenBank&RID=Z83S00BC112&log$=nuclalign&blast_rank=0) 226 ................C..............T.................C..T........................................................G.......... 107

<=========================**708R1**

**708**: ATTGGGGAAACGAACCGCTGTATCTCGAATTATGGAGGCAGTAATGCAGCTTGACATGAATAAAATGCAT

[BT101740](http://www.ncbi.nlm.nih.gov/nuccore/270134788) 361 AGCCAAATTAACTGCGAAATTGGGGAAACGAACCGCTGTATCTCGAATTATGGAGGCAGTAATGCAGCTTGACATGAATAAAATGCATGCATACCTTTACAATAACTAAATCATTTTGAT 480

[CO254451](http://www.ncbi.nlm.nih.gov/entrez/query.fcgi?cmd=Retrieve&db=Nucleotide&list_uids=49137570&dopt=GenBank&RID=Z83S00BC112&log$=nuclalign&blast_rank=0) 201 ..............................................C......................................................................... 82

[CO252529](http://www.ncbi.nlm.nih.gov/entrez/query.fcgi?cmd=Retrieve&db=Nucleotide&list_uids=49133219&dopt=GenBank&RID=Z83S00BC112&log$=nuclalign&blast_rank=0) 242 ..............................................C......................................................................... 123

[DR590294](http://www.ncbi.nlm.nih.gov/entrez/query.fcgi?cmd=Retrieve&db=Nucleotide&list_uids=70655037&dopt=GenBank&RID=Z83S00BC112&log$=nuclalign&blast_rank=0) 106 .......C.......C................T..T...................A..--------------------------------------------------------------

[BT101740](http://www.ncbi.nlm.nih.gov/nuccore/270134788) 481 GTTTTGAAGTGTTTCAGATTGGGGTGGAGTTGAAAATATAGATCGAGTGTATAATTTATTTTCTATGAGTTTGCCTTTCGG-AAAAACTCATTTTAAATGGAAATGATGATTATTGTTAAAAA 602

[CO254451](http://www.ncbi.nlm.nih.gov/entrez/query.fcgi?cmd=Retrieve&db=Nucleotide&list_uids=49137570&dopt=GenBank&RID=Z83S00BC112&log$=nuclalign&blast_rank=0) 81 .......................................................G....... 21

[CO252529](http://www.ncbi.nlm.nih.gov/entrez/query.fcgi?cmd=Retrieve&db=Nucleotide&list_uids=49133219&dopt=GenBank&RID=Z83S00BC112&log$=nuclalign&blast_rank=0) 122 ....................................................................A.........T..G.................................-..C. 4

[DR590294](http://www.ncbi.nlm.nih.gov/entrez/query.fcgi?cmd=Retrieve&db=Nucleotide&list_uids=70655037&dopt=GenBank&RID=Z83S00BC112&log$=nuclalign&blast_rank=0) -----------------------------------------------------------------------------------------------------AAATGATGATTATTGTTATTCTC

## Proline-rich (14715)

**14715**: GTGTATGCAAACTCTCAGTCAAACAAACGTCCATAGGCATAACTATTATCATGGATCCGCCACCGCCTCC

[BT117672](http://www.ncbi.nlm.nih.gov/nuccore/270150786) 11 ttttagtgtatgcAAACTCTCAGTCAAACAAACGTCCATAGGCATAACTATTATC**ATG**GATCCGCCACCGCCTCCGGGAGGACCTCCTGGCTTTGATCCCTTCGGTCCTCCTCCGCCTGC 143

[DR569142](http://www.ncbi.nlm.nih.gov/entrez/query.fcgi?cmd=Retrieve&db=Nucleotide&list_uids=70633859&dopt=GenBank&RID=Z86X1B66112&log$=nuclalign&blast_rank=0) 1 GGTAAACTCTCAGTCAAACAAACGTTCATAGGCATAACTATTATC**ATG**GATCCGCCACCGCCTCCGGGAGGACCTCCTGGCTTTGATCCCTTCGGTCCTCCTCCGCCTGC 123

[DR562030](http://www.ncbi.nlm.nih.gov/entrez/query.fcgi?cmd=Retrieve&db=Nucleotide&list_uids=70626237&dopt=GenBank&RID=Z86X1B66112&log$=nuclalign&blast_rank=0) 463 TGGAAACTCTCAGTCAAACAAACGTTCATAGGCATAACTATTATC**ATG**GATCCGCCACCGCCTCCGGGAGGACCTCCTGGCTTTGATCCCTTCGGTCCTCCTCCGCCTGC 341

14aa (42 bases)  **F2=======================>**

[BT117672](http://www.ncbi.nlm.nih.gov/nuccore/270150786) 144 ------------------------------------------TTTGATCCATTCGCTCCTCCTCCGCCTGCTCCAGGGCCTCCGTTTGATCCATTCGCTCCT 203

[DR569142](http://www.ncbi.nlm.nih.gov/entrez/query.fcgi?cmd=Retrieve&db=Nucleotide&list_uids=70633859&dopt=GenBank&RID=Z86X1B66112&log$=nuclalign&blast_rank=0) 124 TTTGATCCATTCGCTCCTCCTCCGTCTGCACCAGGGCCTCCGTTTGATCCATTCGCTCCTCCTCCGCCTGCTCCAGGGCCTCCGTTTGATCCATTCGCTCCT 225

[DR562030](http://www.ncbi.nlm.nih.gov/entrez/query.fcgi?cmd=Retrieve&db=Nucleotide&list_uids=70626237&dopt=GenBank&RID=Z86X1B66112&log$=nuclalign&blast_rank=0) 340 TTTGATCCATTCGCTCCTCCTCCGTCTGCACCAGGGCCTCCGTTTGATCCATTCGCTCCTCCTCCGCCTGCTCCAGGGCCTCCGTTTGATCCATTCGCTCCT 239

**14715F1**=======================> <========================**14715R1**

[BT117672](http://www.ncbi.nlm.nih.gov/nuccore/270150786) GCCAGGGCCTCCACCTCCACCAGGTCCTATGGGTGCCCCACCGCCGCCTGGTCCTCCAGGCGGGCCTCCTCCTCCTGGCCCTCCATTTCCT**TGA**AGGAAGAGTTTATGTATACACAGGTCTCAGCTAGACTAG 323

[DR569142](http://www.ncbi.nlm.nih.gov/entrez/query.fcgi?cmd=Retrieve&db=Nucleotide&list_uids=70633859&dopt=GenBank&RID=Z86X1B66112&log$=nuclalign&blast_rank=0) GCCAGGGCCTCCACCTCCACCAGGTCCTATGGGTGCCCCACCGCCGCCTGGTCCTCCAGGCGGGCCTCCTCCTCCTGGCCCTCCATTTCCT**TGA**AGGAAGAGTTTATGTATACACAGGTCTCAACTAGACTAG 345

[DR562030](http://www.ncbi.nlm.nih.gov/entrez/query.fcgi?cmd=Retrieve&db=Nucleotide&list_uids=70626237&dopt=GenBank&RID=Z86X1B66112&log$=nuclalign&blast_rank=0) GCCAGGGCCTCCACCTCCACCAGGTCCTATGGGTGCCCCACCGCCGCCTGGTCCTCCAGGCGGGCCTCCTCCTCCTGGCCCTCCATTTCCT**TGA**AGGAAGAGTTTATGTATACACAGGTCTCAACTAGACTAG 119

[CO235728](http://www.ncbi.nlm.nih.gov/entrez/query.fcgi?cmd=Retrieve&db=Nucleotide&list_uids=49058043&dopt=GenBank&RID=Z86X1B66112&log$=nuclalign&blast_rank=0) 299 CCTCCAGGCGGGCCTCCTCCTCCTGGCCCTCCATTTCCT**TGA**AGGAAGAGTTTATGTATACACAGGTCTCAGCTAGACTAG 219

[CO240338](http://www.ncbi.nlm.nih.gov/entrez/query.fcgi?cmd=Retrieve&db=Nucleotide&list_uids=49062655&dopt=GenBank&RID=Z86X1B66112&log$=nuclalign&blast_rank=0) 251 CTTGAAGGAAGAGTTTATGTATACACAGGTCTCAGCTAGACTAG 208

[BT117672](http://www.ncbi.nlm.nih.gov/nuccore/270150786) 324 AACTCCCTATTCTCTATGGTATATCTTATCTTATCTTTTGCAGTAGCCTAAGATATCTACTTAAGTGGTATACTGTGACTTTCTCTGCTACTTCTATATTGAAATCATGAGTTCTTTAAT 443

[DR569142](http://www.ncbi.nlm.nih.gov/entrez/query.fcgi?cmd=Retrieve&db=Nucleotide&list_uids=70633859&dopt=GenBank&RID=Z86X1B66112&log$=nuclalign&blast_rank=0) 346 AACTCCCTATTCTCTATGGTATATCTTATCTTATCTTTTGCAGTAGCCTAAGATATCTACTTAAGTGGTATACTGTGACTTTCTCTGCTACTTCTATATTGAAATCATGAGTTCTTTA 463

[DR562030](http://www.ncbi.nlm.nih.gov/entrez/query.fcgi?cmd=Retrieve&db=Nucleotide&list_uids=70626237&dopt=GenBank&RID=Z86X1B66112&log$=nuclalign&blast_rank=0) 118 AACTCCCTATTCTCTATGGTATATCTTATCTTATCTTTTGCAGTAGCCTAAGATATCTACTTAAGTGGTATACTGTGACTTTCTCTGCTACTTCTATATTGAAATCATGAGTTCTTTA 1

[CO235728](http://www.ncbi.nlm.nih.gov/entrez/query.fcgi?cmd=Retrieve&db=Nucleotide&list_uids=49058043&dopt=GenBank&RID=Z86X1B66112&log$=nuclalign&blast_rank=0) 218 AACTCCCTATTCTCTATGGTATATCTTATCTTATCTTTTGCAGTAGCCTAAGATATCTACTTAAGTGGTATACTGTGACTTTCTCTGCTACTTCTATATTGAAATCATGAGTTCTTTAAT 99

[CO240338](http://www.ncbi.nlm.nih.gov/entrez/query.fcgi?cmd=Retrieve&db=Nucleotide&list_uids=49062655&dopt=GenBank&RID=Z86X1B66112&log$=nuclalign&blast_rank=0) 207 AACTCCCTATTCTCTATGGTATATCTTATCTTATCTTTTGCAGTAGCCTAAGATATCTACTTAAGTGGTATACTGTGACTTTCTCTGCTACTTCTGTATTGAAATCATGAGTTCTTTAAT 88

[BT117672](http://www.ncbi.nlm.nih.gov/nuccore/270150786) 444 GTTACTATATCGAAACGTGTATCTTA-------GCATTTCAATGGAATTAATATCTATCAGTAAAGATGAAATTTTAGAGATAAGAAAAAA 527

[CO235728](http://www.ncbi.nlm.nih.gov/entrez/query.fcgi?cmd=Retrieve&db=Nucleotide&list_uids=49058043&dopt=GenBank&RID=Z86X1B66112&log$=nuclalign&blast_rank=0) 98 GTTACTATATCGTAACCTGTATCTTAAATGTTTGCATTTTAATGGAATTAATATCTATTGGTAAAGATGGAATTTTAGAGAAAAAAAAAAA 8

[CO240338](http://www.ncbi.nlm.nih.gov/entrez/query.fcgi?cmd=Retrieve&db=Nucleotide&list_uids=49062655&dopt=GenBank&RID=Z86X1B66112&log$=nuclalign&blast_rank=0) 87 GTTACTATATCGTAACGTGTATCTTAAATGCTTGCATTTTAATGGAATTAATATCTATTGGTAAAG 22

# EST sequence alignments that include the microarray probes (red) and qPCR primers (arrows) for the G12UP candidate genes

## PgPI20a (25680)

[EX308802](http://www.ncbi.nlm.nih.gov/nucest/EX308802.1) 1 CCGATCTCTGATATCAAACGACA**ATG**GCGTGCTCACGAAATTTTCTTGTTATGCAAATCCTGTGCCTTTCAGCAGGAGTTATGGTTCTATCAATGCTGCTCTGCACTAGTGCAGAAGGAA 61

[DR581877](http://www.ncbi.nlm.nih.gov/entrez/query.fcgi?cmd=Retrieve&db=Nucleotide&list_uids=70646620&dopt=GenBank&RID=Z3C827JV11R&log$=nuclalign&blast_rank=0) 1 CA**ATG**GCGTGCTCACGAAATTTTCTTGTTATGCAAATCCTGTGCCTTTCAGCAGGAGTTATGCTTCTATCAATGCTGCTCTGCACTAGTGCAGAAGGAA 40

[DR585477](http://www.ncbi.nlm.nih.gov/entrez/query.fcgi?cmd=Retrieve&db=Nucleotide&list_uids=70650220&dopt=GenBank&RID=Z3C827JV11R&log$=nuclalign&blast_rank=0) 569 CCGATCTCTGATATCAAACAACA**ATG**GCGTGCTCACGAAATTTTCTTGTTATGCAAATTCTGTGCCTTTCAGCAGGAGTTATGCTTCTATCAATGCTGCTCTGCACTAGTGCAGAAGGAA 450

**25680F1**======================>

[EX308802](http://www.ncbi.nlm.nih.gov/nucest/EX308802.1) 121 ATGGGGTGACTGGTCAGCATCGCAGGCTGCTACAAGTGTGTGTTCGCAATTGCTTGGATGTGTCATACACGACTTGCACGTCGTCGGGAAACGAACAACTCCAGGCCTACTGCAACTGCT 240

[DR581877](http://www.ncbi.nlm.nih.gov/entrez/query.fcgi?cmd=Retrieve&db=Nucleotide&list_uids=70646620&dopt=GenBank&RID=Z3C827JV11R&log$=nuclalign&blast_rank=0) 100 ATGGGGTGACTGGTCAGCATCGCAGGCTGCTACAAGTGTGTGTTCGCAATTGCTTGGATGTGTCATACACGACTTGCACGTCGTCGGGAAACGAACAACTCCAGGCCTACTGCAACTGCT 219

[DR585477](http://www.ncbi.nlm.nih.gov/entrez/query.fcgi?cmd=Retrieve&db=Nucleotide&list_uids=70650220&dopt=GenBank&RID=Z3C827JV11R&log$=nuclalign&blast_rank=0) 449 ATGGGGTGACTGGTCAGCATCGCAGGCTGCTACAAGTGTGTGTTCGCAATTGCTTGGATGTGTCATACACGACTTGCACGTCGTCGGGAAACGAACAACTCCAGGCCTACTGCAACTGCT 330

<==========================**25680R1**

[EX308802](http://www.ncbi.nlm.nih.gov/nucest/EX308802.1) 241 GTTTTGCGCGAGCAGTCATACCGGAAGGGGGCAGCTGCACTCTGCATCTTACCAATGGCACCCAAGTTACTTGTCCTTACTTTCACTGTGTGGGCAAGTGTGAT**TAA**ATAACAGGGAGAT 360

[DR581877](http://www.ncbi.nlm.nih.gov/entrez/query.fcgi?cmd=Retrieve&db=Nucleotide&list_uids=70646620&dopt=GenBank&RID=Z3C827JV11R&log$=nuclalign&blast_rank=0) 220 GTTTTGCGCGAGCAGTCATACCGGAAGGGGGCAGCTGCACTCTGCATCTTACCAATGGCACCCAAGTTACTTGTCCTTACTTTCACTGTGTGGGCAAGTGTGAT**TAA**ATAACAGGGAGAT 339

[DR585477](http://www.ncbi.nlm.nih.gov/entrez/query.fcgi?cmd=Retrieve&db=Nucleotide&list_uids=70650220&dopt=GenBank&RID=Z3C827JV11R&log$=nuclalign&blast_rank=0) 329 GTTTTGCGCGAGCAGTCATACCGGAAGGGGGCAGCTGCACTCTGCATCTTACCAATGGCACCCAAGTTACTTGTCCTTACTTTCACTGTGTGGGCAAGTGTGAT**TAA**ATAACAGGGAGAT 210

**25680:** TAACGCTTCTGGAACTCTACAGATGTGTCTGTTTCGGTATCTGAGTATGAAGAATAAGAGGC

[EX308802](http://www.ncbi.nlm.nih.gov/nucest/EX308802.1) 361 TGAAAGCAAGAATAAAAACAGTCAGATATGTCTGT----------------TAACGCTTCTGGAACTCTACAGATGTGTCTGTTTCGGTATCTGAGTATGAAGAATAAGAGGC 457

[DR581877](http://www.ncbi.nlm.nih.gov/entrez/query.fcgi?cmd=Retrieve&db=Nucleotide&list_uids=70646620&dopt=GenBank&RID=Z3C827JV11R&log$=nuclalign&blast_rank=0) 340 TGAAAGCAAGAATAAAAACAGTCAGATATGTCTGTTAATGAATAATACCCATAACGCTTCTGGAACTCTACAGATGTGTATGTTTCGGTATCTGAGTATGAAGAATAAGAGGC 452

[DR585477](http://www.ncbi.nlm.nih.gov/entrez/query.fcgi?cmd=Retrieve&db=Nucleotide&list_uids=70650220&dopt=GenBank&RID=Z3C827JV11R&log$=nuclalign&blast_rank=0) 209 TGGAA *CTCTA* ----------CAGATATGTCTGTTAATGAATAATACCCATAACGCTTCTGGAACTCTACAGATGTGTATGTTTCGGTTTCTAAGTATGAAGAATAAGAGGC 109

CATACGCC

[EX308802](http://www.ncbi.nlm.nih.gov/nucest/EX308802.1) 458 CATACGCCAATACTTTTCTCTGGATATGTTTGTTTTCTAATTCTGGAATGAATAATA-----------------TTGGAATACTTTTACTACTAAAAAA

[DR581877](http://www.ncbi.nlm.nih.gov/entrez/query.fcgi?cmd=Retrieve&db=Nucleotide&list_uids=70646620&dopt=GenBank&RID=Z3C827JV11R&log$=nuclalign&blast_rank=0) 453 CATACGCCAATACTTTTCTCTGCATATGTATGTTTTCTAATTCTGGACTGAATAAgag *gccatacgcc* --------aatacttttcc 531

[DR585477](http://www.ncbi.nlm.nih.gov/entrez/query.fcgi?cmd=Retrieve&db=Nucleotide&list_uids=70650220&dopt=GenBank&RID=Z3C827JV11R&log$=nuclalign&blast_rank=0) 108 CATACGCCAATAGTTTTCTCTGGATATGTATGTTTTCTAATTCTGGAATGAATAATAGCATTCCTCTATTTTTATTGGAATAATTTTACGAAAAAAAAAAAAAAAAAA

## PgPI20b (22825)

[EX422557](http://www.ncbi.nlm.nih.gov/nucest/EX422557.1) 2 TCAAACGTAAGATCAGTGAAGTGGGTACAAGTTGCTCGCGCATTAACCGAACAACAAAAAGAAATCATC**ATG**GCCCTCTGCACACGAACAAAAAATCTTCTTCTTTTTTCAGCAGGTATT 121

[EX309996](http://www.ncbi.nlm.nih.gov/entrez/query.fcgi?cmd=Retrieve&db=Nucleotide&list_uids=157508765&dopt=GenBank&RID=Z5J6J4VB111&log$=nuclalign&blast_rank=0) 3 GAATAACAAAAAGAAATCATCATGGCCCTCTGCACACGAACACAAATTCTTCTTCTTCTTTCAACAGGTATT 74

[DR586576](http://www.ncbi.nlm.nih.gov/entrez/query.fcgi?cmd=Retrieve&db=Nucleotide&list_uids=70651319&dopt=GenBank&RID=Z5J6J4VB111&log$=nuclalign&blast_rank=0) 578 GGTACAAGTTGCGCGCGCATTAGCCGAATAACAAAAAGAAATCATCATGGCCCTCTGCACACGAACACAAATTCTTCTTCTTCTTTCAACAGGTATT 482

[DR586739](http://www.ncbi.nlm.nih.gov/entrez/query.fcgi?cmd=Retrieve&db=Nucleotide&list_uids=70651482&dopt=GenBank&RID=Z5J6J4VB111&log$=nuclalign&blast_rank=0) 577 GGTACAAGTTGCGCGCGCATTAGCCGAATAACAAAAAGAAATCATCATGGCCCTCTGCACACGACCACAAATTCTTTTTATTCTTTCAACAGGTATT 481

[DR585988](http://www.ncbi.nlm.nih.gov/entrez/query.fcgi?cmd=Retrieve&db=Nucleotide&list_uids=70650731&dopt=GenBank&RID=Z5J6J4VB111&log$=nuclalign&blast_rank=0) 665 TCAAATGTAAGATCAGTGAAGTGGGTACAAGTTGCTCGCGCATTACCCGAACAACAAAATGAAATCATCATGGCCCTCTGCACACGAACAAAAATTCTTCTTATTCTTTCAGCAGGTATT 546

[EX422557](http://www.ncbi.nlm.nih.gov/nucest/EX422557.1) 122 ATGCTTCTCACAGCAACAATGTTGGCATCCAGTGCAGAAGCGTGTCCGCAATACTGCCTGGACGTGTCATACACGACTTGCACGGCCTCGGGTGACCAAGAATTAAAAGCATA-CTGCAA 240

[EX309996](http://www.ncbi.nlm.nih.gov/entrez/query.fcgi?cmd=Retrieve&db=Nucleotide&list_uids=157508765&dopt=GenBank&RID=Z5J6J4VB111&log$=nuclalign&blast_rank=0) 75 ATGCTTCTCACAGCAACAATGTTGGCATCCAGTGCAGAAGCGTGTCCGCAATACTGCCTGGACGTGTCATACACGACTTGCACGGCCTCGGGTGACCAAGAATTAAAAGCATA-CTGCAA 193

[DR586576](http://www.ncbi.nlm.nih.gov/entrez/query.fcgi?cmd=Retrieve&db=Nucleotide&list_uids=70651319&dopt=GenBank&RID=Z5J6J4VB111&log$=nuclalign&blast_rank=0) 481 ATGCTTCTCACAGCAACAATGTTGGCATCCAGTGCAGAAGCGTGTCCGCAATTCTGCCTGGACGTGTCATACACGACTTGCACGGCCTCGGGTGACCAAGAATTAAAAGCATA-CTGCAA 363

[DR586739](http://www.ncbi.nlm.nih.gov/entrez/query.fcgi?cmd=Retrieve&db=Nucleotide&list_uids=70651482&dopt=GenBank&RID=Z5J6J4VB111&log$=nuclalign&blast_rank=0) 480 ATGCTTCTCACAGCAACAATGTTGGCATCCAGTGCAGAAGCGTGTCCGCAATTCTCCCTGGACGTGTCATACACGACTTGCACGGCCTCGGGTGACCAAGAATTAAAAGCATA-CTGCAA 362

[DR585988](http://www.ncbi.nlm.nih.gov/entrez/query.fcgi?cmd=Retrieve&db=Nucleotide&list_uids=70650731&dopt=GenBank&RID=Z5J6J4VB111&log$=nuclalign&blast_rank=0) 395 AGCGTGTCCGCAATGCTGCCTGGACGTGTCATACAAGACTTGCAAGGCGTAGGGTGACCAAGAATTAAAAGCATAGGTGCAA 314

[DR585988](http://www.ncbi.nlm.nih.gov/entrez/query.fcgi?cmd=Retrieve&db=Nucleotide&list_uids=70650731&dopt=GenBank&RID=Z5J6J4VB111&log$=nuclalign&blast_rank=0) 545 ATGATTGTCACAGCAACAATGTTGGCATCCAGTGCAGA*AG-GTG* 503

**22825F1**====================> **22825**: GCTCTCCTGTTCCTGATCCAATTACCCAGAGATTCAGAATTGGAAA

[EX422557](http://www.ncbi.nlm.nih.gov/nucest/EX422557.1) 241 CTGCTGTTTTCTCAAAGGAGCGATGCCCAACGCCG-GCCAGTAC-ACTC-TGCATCTCAGCGATGGCACCCAGCTCTCCTGTTCC**TGA**TCCAATTACCCAGAGATTCAGAATTGGAAA 355

<=======================**22825R1**

[EX309996](http://www.ncbi.nlm.nih.gov/entrez/query.fcgi?cmd=Retrieve&db=Nucleotide&list_uids=157508765&dopt=GenBank&RID=Z5J6J4VB111&log$=nuclalign&blast_rank=0) 194 CTGCTGCTTTCTCAAAGGAGCGATGCCCAACGCCG-GCCAGTGC-ACTC-TGCATCTCAGCGATGGCACCCAGCTCTCCTGTTCCTGATCCAATTACCCAGAGATTCAGAATTGGAAA 308

[DR586576](http://www.ncbi.nlm.nih.gov/entrez/query.fcgi?cmd=Retrieve&db=Nucleotide&list_uids=70651319&dopt=GenBank&RID=Z5J6J4VB111&log$=nuclalign&blast_rank=0) 362 CTCCTGCTTTCTCAAAGGAGCGATGCCCAACGCCG-GCCAGTGC-ACTG-TGCATCTCAGCGATGGCACCCAGCTCTCCTGTTCCTGATCCAATTACCCAGAGATTCAGAATTGGAAA 248

[DR586739](http://www.ncbi.nlm.nih.gov/entrez/query.fcgi?cmd=Retrieve&db=Nucleotide&list_uids=70651482&dopt=GenBank&RID=Z5J6J4VB111&log$=nuclalign&blast_rank=0) 361 CTCATGCTTTCTCAAAGGAGCGATGCCCAACGCCG-GCCAGTGC-ACTC-TGCATTTCAGCGATGGCACCCAGCTCTCCTGTTCCTGATCCAATTCCCCAGAGATTCAGAATTGGAAA 247

[DR585988](http://www.ncbi.nlm.nih.gov/entrez/query.fcgi?cmd=Retrieve&db=Nucleotide&list_uids=70650731&dopt=GenBank&RID=Z5J6J4VB111&log$=nuclalign&blast_rank=0) 313 CTCATGCTTTTTCAAAGGAGCGATGCCCAACGCCGCGCCAGTGCGACTCGTGCATTTCAGCGATGGCACCCAGCTGTCCTGTTCCTGATCCAATTACCCAGAGATTCAGAATTGGAAA 196

GCTGGTAGTTCAAAATTGTCTTTC

[EX422557](http://www.ncbi.nlm.nih.gov/nucest/EX422557.1) 356 GCTGGTAGTTCAAAATTGTCTTTCATCTACTTCCTGCATCATATTATATTTTACAGTATGTACCTCAACGTGGATGTGCGTCCACAAAGTCTATGCAACCACTACCCAGTGGATGC-TA 473

[EX309996](http://www.ncbi.nlm.nih.gov/entrez/query.fcgi?cmd=Retrieve&db=Nucleotide&list_uids=157508765&dopt=GenBank&RID=Z5J6J4VB111&log$=nuclalign&blast_rank=0) 309 GCTGGTAGTTCAAAATTGTCTTTCATCTACTTCCTGCATCATATTATATTTTACAGTATGTACCTCAACGTGGATGTGCGTCCACAAAGTCTATGCAACCACTACCCAGTGGATGC-TA 426

[DR586576](http://www.ncbi.nlm.nih.gov/entrez/query.fcgi?cmd=Retrieve&db=Nucleotide&list_uids=70651319&dopt=GenBank&RID=Z5J6J4VB111&log$=nuclalign&blast_rank=0) 247 GCTGGTAGTTCAAAATTGTCTTTCATCTACTTCCTGCATCATATTATATTTTACAGCATGTTCCTCAACGTGGATGTGCGTCCACAAAGTCTATGCAACCTCTACCCAGTGGATGCATA 129

[DR586739](http://www.ncbi.nlm.nih.gov/entrez/query.fcgi?cmd=Retrieve&db=Nucleotide&list_uids=70651482&dopt=GenBank&RID=Z5J6J4VB111&log$=nuclalign&blast_rank=0) 246 GCTGGTAGTTCAAAATTGTCTTTCATCTACTTCATGCACCATATTATATTTTACAGCATGTTCCTCAACGTGGATGTGCGTCCACAAAGTCTATGCAACCTCTACCCAGTGGATGC-TA 129

[DR585988](http://www.ncbi.nlm.nih.gov/entrez/query.fcgi?cmd=Retrieve&db=Nucleotide&list_uids=70650731&dopt=GenBank&RID=Z5J6J4VB111&log$=nuclalign&blast_rank=0) 195 GCTGCTAGTTCAAAATTGTCTTTCATTTACTTCATGCATCATATTATATTTTTCAGTCTGTACCTCAAAGAGGACGTGTGTCCGCAAAGTTTAGGCAACCATTACCCAGTGGATAG-TA 78

[EX422557](http://www.ncbi.nlm.nih.gov/nucest/EX422557.1) 474 TTAAATAAATGAGGGGAGTCCAAACATTCCCACGATCTGGACTCTATTAGATAC-TGTGTTATTCATGTATCCTGTGATTTAAATTTAAAGATTTACATAT 573

[EX309996](http://www.ncbi.nlm.nih.gov/entrez/query.fcgi?cmd=Retrieve&db=Nucleotide&list_uids=157508765&dopt=GenBank&RID=Z5J6J4VB111&log$=nuclalign&blast_rank=0) 427 TTAAATAAATGAGGGGAGTCCAAACATTCCCACGATCTGGACTCTATTAGATAC-TGTGTTATTCATGTATCCTGTGATTTAAATTTAAAGATTTACATAT 526

[DR586576](http://www.ncbi.nlm.nih.gov/entrez/query.fcgi?cmd=Retrieve&db=Nucleotide&list_uids=70651319&dopt=GenBank&RID=Z5J6J4VB111&log$=nuclalign&blast_rank=0) 128 TTAAATAAATGAGGGGAGTCCAAACATTCCCACGATCTGGCCTCTATTAGATCC-TGTGTTATTCATGTATCCTGTGATTTAAATTTAAAGATTCACATAT 29

[DR586739](http://www.ncbi.nlm.nih.gov/entrez/query.fcgi?cmd=Retrieve&db=Nucleotide&list_uids=70651482&dopt=GenBank&RID=Z5J6J4VB111&log$=nuclalign&blast_rank=0) 128 TCAAATAAATGAGGGGAGTCCAACCATTCCCACGATCTGGCCTCTATTAGATCC-TGTGTTATTCATGTATCCTGTGATTTAAATTTAAAGATTCACATAT 29

[DR585988](http://www.ncbi.nlm.nih.gov/entrez/query.fcgi?cmd=Retrieve&db=Nucleotide&list_uids=70650731&dopt=GenBank&RID=Z5J6J4VB111&log$=nuclalign&blast_rank=0) 77 TTAAATAAATGAGGGGAGTACAAACATTGCCACGATCTGGACTCTATTAGATACATGTGTTATTCATGTATCCTGTG

## PgPrx21 (16777)

[DV984746](http://www.ncbi.nlm.nih.gov/nucest/211868939) 1 GCATACCCCAGTCGTGCGATGCCTCCATTCTGTTAGAC-AATAGTGGAGATGTGCAGTCGGAGAAACAATCGGATCGGAACTTCGGA**ATG**CGAAACTTCAAGTATATGGACACCATTAAG 119

[DV986066](http://www.ncbi.nlm.nih.gov/entrez/query.fcgi?cmd=Retrieve&db=Nucleotide&list_uids=211856052&dopt=GenBank&RID=Z5R2ZHW1113&log$=nuclalign&blast_rank=0) 884 .....GT...............................A................................................................................. 765

[EX307930](http://www.ncbi.nlm.nih.gov/entrez/query.fcgi?cmd=Retrieve&db=Nucleotide&list_uids=157506699&dopt=GenBank&RID=Z5R2ZHW1113&log$=nuclalign&blast_rank=0) 188 .....GT...............................-................................................................................. 306

[EX307114](http://www.ncbi.nlm.nih.gov/entrez/query.fcgi?cmd=Retrieve&db=Nucleotide&list_uids=157505883&dopt=GenBank&RID=Z5R2ZHW1113&log$=nuclalign&blast_rank=0) 205 .....GT...............................-................................................................................. 323

[EX307261](http://www.ncbi.nlm.nih.gov/entrez/query.fcgi?cmd=Retrieve&db=Nucleotide&list_uids=157506030&dopt=GenBank&RID=Z5R2ZHW1113&log$=nuclalign&blast_rank=0) 200 .....GT...............................-................................................................................. 318

[DV984746](http://www.ncbi.nlm.nih.gov/nucest/211868939) 60 GAGAAACAATCGGATCGGAACTTCGGAATGCGAAACTTCAAGTATATGGACACCATTAAGAAGGCCGTCGAGGCGGAATGTCCTGGAGTGGTGTCGTGTGCTGACATTATTTCTCTAGCC 179

[DV986066](http://www.ncbi.nlm.nih.gov/entrez/query.fcgi?cmd=Retrieve&db=Nucleotide&list_uids=211856052&dopt=GenBank&RID=Z5R2ZHW1113&log$=nuclalign&blast_rank=0) 824 ........................................................................................................................ 705

[EX307930](http://www.ncbi.nlm.nih.gov/entrez/query.fcgi?cmd=Retrieve&db=Nucleotide&list_uids=157506699&dopt=GenBank&RID=Z5R2ZHW1113&log$=nuclalign&blast_rank=0) 247 ........................................................................................................................ 366

[EX307114](http://www.ncbi.nlm.nih.gov/entrez/query.fcgi?cmd=Retrieve&db=Nucleotide&list_uids=157505883&dopt=GenBank&RID=Z5R2ZHW1113&log$=nuclalign&blast_rank=0) 264 ........................................................................................................................ 383

[EX307261](http://www.ncbi.nlm.nih.gov/entrez/query.fcgi?cmd=Retrieve&db=Nucleotide&list_uids=157506030&dopt=GenBank&RID=Z5R2ZHW1113&log$=nuclalign&blast_rank=0) 259 ........................................................................................................................ 378

[DV984746](http://www.ncbi.nlm.nih.gov/nucest/211868939) 180 GCAAGAGAGGGAGCTGCAATGCTAGGAGGGCCACGCATCGCTGTGAAAACAGGGAGGCTAGACAGCAGAAAAAGCAGTGCAGCAGTGGTGGACAGATACACTCCACTGCATAACGACAGC 299

[DV986066](http://www.ncbi.nlm.nih.gov/entrez/query.fcgi?cmd=Retrieve&db=Nucleotide&list_uids=211856052&dopt=GenBank&RID=Z5R2ZHW1113&log$=nuclalign&blast_rank=0) 704 ........................................................................................................................ 585

[EX307930](http://www.ncbi.nlm.nih.gov/entrez/query.fcgi?cmd=Retrieve&db=Nucleotide&list_uids=157506699&dopt=GenBank&RID=Z5R2ZHW1113&log$=nuclalign&blast_rank=0) 367 .............................AT......................................................................................... 486

[EX307114](http://www.ncbi.nlm.nih.gov/entrez/query.fcgi?cmd=Retrieve&db=Nucleotide&list_uids=157505883&dopt=GenBank&RID=Z5R2ZHW1113&log$=nuclalign&blast_rank=0) 384 ........................................................................................................................ 503

[EX307261](http://www.ncbi.nlm.nih.gov/entrez/query.fcgi?cmd=Retrieve&db=Nucleotide&list_uids=157506030&dopt=GenBank&RID=Z5R2ZHW1113&log$=nuclalign&blast_rank=0) 379 .............................AT......................................................................................... 498

[DV984746](http://www.ncbi.nlm.nih.gov/nucest/211868939) 300 ATCTCAACTCTTCTCTCCGCCTTTGCCTCTGCAGGCATCGATGCGGAAGGAGCCGTAGCCCTTCTAGGAGCACATAGTGTGGGCAGAACCCACTGTGTAAATCTGGTTGAGAGGCTTTAC 419

[DV986066](http://www.ncbi.nlm.nih.gov/entrez/query.fcgi?cmd=Retrieve&db=Nucleotide&list_uids=211856052&dopt=GenBank&RID=Z5R2ZHW1113&log$=nuclalign&blast_rank=0) 584 ........................................................................................................................ 465

[EX307930](http://www.ncbi.nlm.nih.gov/entrez/query.fcgi?cmd=Retrieve&db=Nucleotide&list_uids=157506699&dopt=GenBank&RID=Z5R2ZHW1113&log$=nuclalign&blast_rank=0) 487 ........................................................G......T........................................................ 606

[EX307114](http://www.ncbi.nlm.nih.gov/entrez/query.fcgi?cmd=Retrieve&db=Nucleotide&list_uids=157505883&dopt=GenBank&RID=Z5R2ZHW1113&log$=nuclalign&blast_rank=0) 504 ........................................................................................................................ 623

[EX307261](http://www.ncbi.nlm.nih.gov/entrez/query.fcgi?cmd=Retrieve&db=Nucleotide&list_uids=157506030&dopt=GenBank&RID=Z5R2ZHW1113&log$=nuclalign&blast_rank=0) 499 ........................................................G......T........................................................ 618

**16777**: AATGACGTCCTCTACGCACGCAACGATCGCGTAACGCCCATG

[DV984746](http://www.ncbi.nlm.nih.gov/nucest/211868939) 420 CCCGTGGTTGATCCGAGTCTTGATCCCGACTACGCTGTATACCTAAAAGGCCGATGCCCGACCCCCAATCCGGACCCCAATGACGTCCTCTACGCACGCAACGATCGCGTAACGCCCATG 539

[DV986066](http://www.ncbi.nlm.nih.gov/entrez/query.fcgi?cmd=Retrieve&db=Nucleotide&list_uids=211856052&dopt=GenBank&RID=Z5R2ZHW1113&log$=nuclalign&blast_rank=0) 464 ........................................................................................................................ 345

[EX307930](http://www.ncbi.nlm.nih.gov/entrez/query.fcgi?cmd=Retrieve&db=Nucleotide&list_uids=157506699&dopt=GenBank&RID=Z5R2ZHW1113&log$=nuclalign&blast_rank=0) 607 ........................................................................................................................ 726

[EX307114](http://www.ncbi.nlm.nih.gov/entrez/query.fcgi?cmd=Retrieve&db=Nucleotide&list_uids=157505883&dopt=GenBank&RID=Z5R2ZHW1113&log$=nuclalign&blast_rank=0) 624 ........................................................................................................................ 743

[EX307261](http://www.ncbi.nlm.nih.gov/entrez/query.fcgi?cmd=Retrieve&db=Nucleotide&list_uids=157506030&dopt=GenBank&RID=Z5R2ZHW1113&log$=nuclalign&blast_rank=0) 619 ........................................................................................................................ 738

**F2=======================>**

AAATTGGACAACAATTATTTCAGAAACT **16777F1**=======================>

[DV984746](http://www.ncbi.nlm.nih.gov/nucest/211868939) 540 AAATTGGACAACAATTATTTCAGAAACTTGATGACGCACAAGGGACTGCTTCTCGTGGACCAGCGCCTGCTCTCGGATCCCAGAACTGGGCCTTACGTCACCAAAATGGCGCAAGACAAC 659

[DV986066](http://www.ncbi.nlm.nih.gov/entrez/query.fcgi?cmd=Retrieve&db=Nucleotide&list_uids=211856052&dopt=GenBank&RID=Z5R2ZHW1113&log$=nuclalign&blast_rank=0) 344 ........................................................................................................................ 225

[EX307930](http://www.ncbi.nlm.nih.gov/entrez/query.fcgi?cmd=Retrieve&db=Nucleotide&list_uids=157506699&dopt=GenBank&RID=Z5R2ZHW1113&log$=nuclalign&blast_rank=0) 727 .........................................................................................A.............................. 846

[EX307114](http://www.ncbi.nlm.nih.gov/entrez/query.fcgi?cmd=Retrieve&db=Nucleotide&list_uids=157505883&dopt=GenBank&RID=Z5R2ZHW1113&log$=nuclalign&blast_rank=0) 744 ............................................................................... 822

[EX307526](http://www.ncbi.nlm.nih.gov/entrez/query.fcgi?cmd=Retrieve&db=Nucleotide&list_uids=157506295&dopt=GenBank&RID=Z5R2ZHW1113&log$=nuclalign&blast_rank=0) 290 .........................................................................................A.............................. 409

[EX307261](http://www.ncbi.nlm.nih.gov/entrez/query.fcgi?cmd=Retrieve&db=Nucleotide&list_uids=157506030&dopt=GenBank&RID=Z5R2ZHW1113&log$=nuclalign&blast_rank=0) 739 ......................................................................... 811

[EX309702](http://www.ncbi.nlm.nih.gov/entrez/query.fcgi?cmd=Retrieve&db=Nucleotide&list_uids=157508471&dopt=GenBank&RID=Z5R2ZHW1113&log$=nuclalign&blast_rank=0) 248 .........................................................................................A.............................. 367

**<============================R2**

<======================**16777R1**

[DV984746](http://www.ncbi.nlm.nih.gov/nucest/211868939) 660 AACTACTTCTTCGCTCAATTCTCAAGA**GCTCTCACTATTCTGTCGGAGAACAATCC**TCTCACTGGCAGCGACGGAGAAATCAGAAAAGATTGCCGTTTTGTGAATCAACCC**TGA**AATCTG 779

[DV986066](http://www.ncbi.nlm.nih.gov/entrez/query.fcgi?cmd=Retrieve&db=Nucleotide&list_uids=211856052&dopt=GenBank&RID=Z5R2ZHW1113&log$=nuclalign&blast_rank=0) 224 ........................................................................................................................ 105

[EX307930](http://www.ncbi.nlm.nih.gov/entrez/query.fcgi?cmd=Retrieve&db=Nucleotide&list_uids=157506699&dopt=GenBank&RID=Z5R2ZHW1113&log$=nuclalign&blast_rank=0) 847 .................... 866

[EX307526](http://www.ncbi.nlm.nih.gov/entrez/query.fcgi?cmd=Retrieve&db=Nucleotide&list_uids=157506295&dopt=GenBank&RID=Z5R2ZHW1113&log$=nuclalign&blast_rank=0) 410 ...................................................C.................................................................... 529

[EX309702](http://www.ncbi.nlm.nih.gov/entrez/query.fcgi?cmd=Retrieve&db=Nucleotide&list_uids=157508471&dopt=GenBank&RID=Z5R2ZHW1113&log$=nuclalign&blast_rank=0) 368 ...................................................C.................................................................... 487

[DV984746](http://www.ncbi.nlm.nih.gov/nucest/211868939) 780 TATTCTCGCCACTACTTCAGCGAAAGCATGACTCTAGTCATTAATGGACGCAGAGTTATCGATTTCTGTACTCTGCCC-AGATTC 863

[DV986066](http://www.ncbi.nlm.nih.gov/entrez/query.fcgi?cmd=Retrieve&db=Nucleotide&list_uids=211856052&dopt=GenBank&RID=Z5R2ZHW1113&log$=nuclalign&blast_rank=0) 104 ..............................................................................G...... 20

[EX307526](http://www.ncbi.nlm.nih.gov/entrez/query.fcgi?cmd=Retrieve&db=Nucleotide&list_uids=157506295&dopt=GenBank&RID=Z5R2ZHW1113&log$=nuclalign&blast_rank=0) 530 .............................................................T................-......

[EX309702](http://www.ncbi.nlm.nih.gov/entrez/query.fcgi?cmd=Retrieve&db=Nucleotide&list_uids=157508471&dopt=GenBank&RID=Z5R2ZHW1113&log$=nuclalign&blast_rank=0) 488 .............................................................T................-......

## PgcwINV1 (22616)

Query 1211 AAGGAAAGACAACGTTGTTTTGGAAAAGGAATCAGTAATGAATGTGGAGGGATTCAACTCAGGAGCGGCGCAGGTGGACGTGGAGGTAGAGTTTGAGTTGGGAGGCGATTATGAAA 1326

[DR581369](http://www.ncbi.nlm.nih.gov/entrez/query.fcgi?cmd=Retrieve&db=Nucleotide&list_uids=70646112&dopt=GenBank&RID=Z5XX404Y111&log$=nuclalign&blast_rank=0) 812 AAGGAAAGACAACGTTGTTTTGGAAAAGGAATCGGTAATGAAGGTGGAGGGATTCAACTCAGGAGCGGCGCAGGTGGACGTGGAGGTAGAGTTTGAGTTGGGAGGCGATTATGAAA 697

[GO366420](http://www.ncbi.nlm.nih.gov/entrez/query.fcgi?cmd=Retrieve&db=Nucleotide&list_uids=225915046&dopt=GenBank&RID=Z5XX404Y111&log$=nuclalign&blast_rank=0) 784 AAAAAAAGACAACGTTGTTTTGGAAAGGAAATCGGTAATGAAGGTGGAGGGATTCAAGTCAGGAGCGGCGCAGGTGGACGTGGAGGTAGAGTTTGAGTTGGGAGGCCATCATGAAA 669

[DR588607](http://www.ncbi.nlm.nih.gov/entrez/query.fcgi?cmd=Retrieve&db=Nucleotide&list_uids=70653350&dopt=GenBank&RID=Z5XX404Y111&log$=nuclalign&blast_rank=0) 1 GGACGTGGAGGTAGAGTTTGAGTTGGGAGGCGATTATGAAA 41

[BT116904](http://www.ncbi.nlm.nih.gov/sites/entrez?cmd=Retrieve&db=nucleotide&dopt=GenBank&RID=Z5VYKGVU011&log$=nuclalign&blast_rank=1&list_uids=270150018) 1327 CAGTGGAGGAGTTGGAGGGAATGACAGCTCAAAGCTTGTGCAGCCGGAACAAGACGAGGGGATTCGGTTTGATGGTATTGGCGTCTGATGATCTGACAGAGAG 1429

[DR581369](http://www.ncbi.nlm.nih.gov/entrez/query.fcgi?cmd=Retrieve&db=Nucleotide&list_uids=70646112&dopt=GenBank&RID=Z5XX404Y111&log$=nuclalign&blast_rank=0) 696 CAGTGGAGGAGTTGGAGGGAATGACAGCTCAAAGCTTGTGCAGTCGGAACAAGACGAGGGGATTCGGTTTGATGGTATTGGCGTTTGATGATCTGACAGAAAG 594

[DR588607](http://www.ncbi.nlm.nih.gov/entrez/query.fcgi?cmd=Retrieve&db=Nucleotide&list_uids=70653350&dopt=GenBank&RID=Z5XX404Y111&log$=nuclalign&blast_rank=0) 42 CAGTGGAGGAGTTGGAGGGAATGACAGCTCAAAGCTTGTGCAGTCGGAACAAGATGAGGGGATTCGGTTTGATGGTATTGGCGTCTGATGATCTGACAGAAAG 144

[GO366420](http://www.ncbi.nlm.nih.gov/entrez/query.fcgi?cmd=Retrieve&db=Nucleotide&list_uids=225915046&dopt=GenBank&RID=Z5XX404Y111&log$=nuclalign&blast_rank=0) 668 AAGTGGAGGAGTTGGAGGGAATGACAGCTCAAAGCTTGTGCAGTCGGAGCAAGACTAGGGGATTCGGGTTGATGGTGTTGGCATCTGATGATCTGAAAGAAAG 566

**22616F2**===================---===>  **22616F1**==========================>

[BT116904](http://www.ncbi.nlm.nih.gov/sites/entrez?cmd=Retrieve&db=nucleotide&dopt=GenBank&RID=Z5VYKGVU011&log$=nuclalign&blast_rank=1&list_uids=270150018) 1430 GAGCACGGTCTTCTCCAACATTTTCAAG**GGCGGAGTTAATGGCAATG---ACAC**AAAGGTGCCCTTGTGCGTTGATCAGAGCAGGTCCACACTGGAACTGGATGTGGATAAGACC 1541

[DR581369](http://www.ncbi.nlm.nih.gov/entrez/query.fcgi?cmd=Retrieve&db=Nucleotide&list_uids=70646112&dopt=GenBank&RID=Z5XX404Y111&log$=nuclalign&blast_rank=0) 593 GAGCACGGTCTTCTTCAAGATTTTCAAGGGCGGAGTTAATGACAATG---ACACAAAGGTAGCTTTGTGCGTGGATCAGAGCAGGTCCACACTGGAACTGGATGTGGATAAGACC 482

[DR588607](http://www.ncbi.nlm.nih.gov/entrez/query.fcgi?cmd=Retrieve&db=Nucleotide&list_uids=70653350&dopt=GenBank&RID=Z5XX404Y111&log$=nuclalign&blast_rank=0) 145 GAGCACGGTCTTCTTCAACATTTTCAAGGGCGGAGTTAATGACAATG---ACACAAAGGTAGCTTTGTGCGTGGATCAGAGCAGGTCCACACTGGAACTGGATGTGGATAAGACC 256

[GO366420](http://www.ncbi.nlm.nih.gov/entrez/query.fcgi?cmd=Retrieve&db=Nucleotide&list_uids=225915046&dopt=GenBank&RID=Z5XX404Y111&log$=nuclalign&blast_rank=0) 565 GAGTGCGGTTTTCTTCAAGGTTTTCAAGGGCAGAGTTAATGGCAATGTCGAGAGAAAGGTGGCCTTGTGCGTGGATCAGAGCAGGTCCACACTGCAATCGGATGTGGATAAGACG 451

<===========================**22616R1**

[BT116904](http://www.ncbi.nlm.nih.gov/sites/entrez?cmd=Retrieve&db=nucleotide&dopt=GenBank&RID=Z5VYKGVU011&log$=nuclalign&blast_rank=1&list_uids=270150018) 1542 GGCTATGGAGGATTTGTGAGCGTGAAACCCCACCAACGCACGCTGTCGCTTAGAGTATTGGTTGATCACTCGATCGTGGAGAGTTATGCAGAGGGAGGGAGAACATGCATTACGTCGAG 1660

[DR581369](http://www.ncbi.nlm.nih.gov/entrez/query.fcgi?cmd=Retrieve&db=Nucleotide&list_uids=70646112&dopt=GenBank&RID=Z5XX404Y111&log$=nuclalign&blast_rank=0) 481 GGCTATGGAGGATTTGTGAGCGTGAAACCCCACCAACGCACGCTGTCGCTTAGAGTATTGGTTGATCACTCGATCGTGGAGAGTTTTGCAGAGGGAGGGAGAACATGCATTACGTCGAG 363

[DR588607](http://www.ncbi.nlm.nih.gov/entrez/query.fcgi?cmd=Retrieve&db=Nucleotide&list_uids=70653350&dopt=GenBank&RID=Z5XX404Y111&log$=nuclalign&blast_rank=0) 257 GGCTATGGAGGATTTGTGAGCGTGAAACCCCACCAACGCACGCTGTCGCTTAGAGTATTGGTTGATCACTCGATCGTGGAAAGTTTTGCAGAGGGAGGGAGAACATGCATTACGTCGAG 375

[GO366420](http://www.ncbi.nlm.nih.gov/entrez/query.fcgi?cmd=Retrieve&db=Nucleotide&list_uids=225915046&dopt=GenBank&RID=Z5XX404Y111&log$=nuclalign&blast_rank=0) 450 GGCTATGGAGAATTTGTGCGGGTGAAATCGAACAAACGTTCGCTGTCGCTCAGAGTATTGGTTGATCACTCGATCGTGGAGAGTTTTGCAGAGGGAGGAAGAACATGCATTACATGGAG 332

[BT116904](http://www.ncbi.nlm.nih.gov/sites/entrez?cmd=Retrieve&db=nucleotide&dopt=GenBank&RID=Z5VYKGVU011&log$=nuclalign&blast_rank=1&list_uids=270150018) 1661 GAGTTATCCGAGCGTGGCAGTTAATGAGAATGCTCATCTCTTCGTCTTTAATTATAAGAATTCGCCCCTCGTCCTTCGTCGACTCTCCGCCTGGCAGATGAACAGCGCCATCCAAGTAT 1779

[DR581369](http://www.ncbi.nlm.nih.gov/entrez/query.fcgi?cmd=Retrieve&db=Nucleotide&list_uids=70646112&dopt=GenBank&RID=Z5XX404Y111&log$=nuclalign&blast_rank=0) 362 GAGTTATCCGAGCGTGGCAGTTAATGAGAATGCTCATCTCTTCGTCTTTAATTATAAGAATTCGCCCCTCGTCCTTCGTCGACTCTCCGCCTGGCAGATGAACAGCGCCATCCAAGTAT 244

[DR588607](http://www.ncbi.nlm.nih.gov/entrez/query.fcgi?cmd=Retrieve&db=Nucleotide&list_uids=70653350&dopt=GenBank&RID=Z5XX404Y111&log$=nuclalign&blast_rank=0) 376 GAGTTATCCGAGCGTGGCAGTTAATGAGAATGCTCATCTCTTCGTCTCTAATTATAAGAAATCGCCCCTCGTCCTTCGTCGACTCTCCGCCTGGCAGATGAACAGCGCCATCCAAGTAT 494

[GO366420](http://www.ncbi.nlm.nih.gov/entrez/query.fcgi?cmd=Retrieve&db=Nucleotide&list_uids=225915046&dopt=GenBank&RID=Z5XX404Y111&log$=nuclalign&blast_rank=0) 331 GAGTTACCCGAGCGTGGCAGTTAATGAGAATGCTCATCTCTTTGTCTTTAATTATAACAATTCGCCCCTCGTCCTTCGTCGACTCTCCGCCTGGCACATGAACACCGCCAAGCAAGTAT 213

**22616**: TGTGGATGTCTCTCCATCGAGACTGAGCTCTTCCATATTG-TC-----TT----ACATTTTGCAATAATCACAAAACCTT

[BT116904](http://www.ncbi.nlm.nih.gov/sites/entrez?cmd=Retrieve&db=nucleotide&dopt=GenBank&RID=Z5VYKGVU011&log$=nuclalign&blast_rank=1&list_uids=270150018) 1780 ACGAATCCAAG**TGA**ATTATGATGTGGATGTCTCTCCATCGAGACTGAGCTCTTCCATATTG-TC-----TT----ACATTTTGCAATAATCACAAAACCTTTTCATTGTCAAAAAA 1885

[DR581369](http://www.ncbi.nlm.nih.gov/entrez/query.fcgi?cmd=Retrieve&db=Nucleotide&list_uids=70646112&dopt=GenBank&RID=Z5XX404Y111&log$=nuclalign&blast_rank=0) 243 ACGAATCCAAGTGAATTATGATGTGGATGTCTCTACATCGAGACTGAGCTCTTCCATATTG-TC-----TT----ACATTTTGCAATAATCACAAAACCTTTTCATTGT 145

[DR588607](http://www.ncbi.nlm.nih.gov/entrez/query.fcgi?cmd=Retrieve&db=Nucleotide&list_uids=70653350&dopt=GenBank&RID=Z5XX404Y111&log$=nuclalign&blast_rank=0) 495 ACGAATCCAAGTGATTTATGATGTGGATGTCTCTCCATCGAGACTGAACTCTTCCATGTTGATCACAAATTTCTTACATTTTGTAATAATCATAAAACCTTTTCATTG-CAAAAAA 609

# EST sequence alignments for the reference genes

## EF1α

~890bp from the polyA **EF1aF1**==========================>

[Sitka EF677538](http://www.ncbi.nlm.nih.gov/nuccore/148908490) 1084 AAGCTGCC-AACTTTACCGCTCAAGTTATTATTATGAATCATCCAGGACAGATTGGAAATGGTTATGCCCCTGTGTTGGATTGCCACACTTGCCACATTGCCGTCAAGTTTTCT-GAGAT 1201

[Sitka EF084956](http://www.ncbi.nlm.nih.gov/entrez/query.fcgi?cmd=Retrieve&db=Nucleotide&list_uids=116786851&dopt=GenBank&RID=ZSASR36A112&log$=nuclalign&blast_rank=0) 1072 ...T....-.........................................................................................................-..... 1189

[Sitka EF085539](http://www.ncbi.nlm.nih.gov/entrez/query.fcgi?cmd=Retrieve&db=Nucleotide&list_uids=116788344&dopt=GenBank&RID=ZSASR36A112&log$=nuclalign&blast_rank=0) 1081 ........-.........................................................................................................-..... 1198

[White spruce BT102965](http://www.ncbi.nlm.nih.gov/entrez/query.fcgi?cmd=Retrieve&db=Nucleotide&list_uids=270136013&dopt=GenBank&RID=ZSASR36A112&log$=nuclalign&blast_rank=0) 1064 ........-.........................................................................................................-..... 1181

[P abies AJ132534](http://www.ncbi.nlm.nih.gov/entrez/query.fcgi?cmd=Retrieve&db=Nucleotide&list_uids=12580860&dopt=GenBank&RID=ZSASR36A112&log$=nuclalign&blast_rank=0) 962 ........-.......................A.................................................................................-..... 1079

[P menziesii AY832581](http://www.ncbi.nlm.nih.gov/entrez/query.fcgi?cmd=Retrieve&db=Nucleotide&list_uids=56481438&dopt=GenBank&RID=ZSASR36A112&log$=nuclalign&blast_rank=0) 373 ........-..............G..................................................A..........................G............-..... 490

[P sylvestris EG418150](http://www.ncbi.nlm.nih.gov/entrez/query.fcgi?cmd=Retrieve&db=Nucleotide&list_uids=116375558&dopt=GenBank&RID=ZSASR36A112&log$=nuclalign&blast_rank=0) 782 ........-...........A..G.......................................................................................G..-..... 665

[P pinaster CT581497](http://www.ncbi.nlm.nih.gov/entrez/query.fcgi?cmd=Retrieve&db=Nucleotide&list_uids=90039770&dopt=GenBank&RID=ZSASR36A112&log$=nuclalign&blast_rank=0) 688 ................G..-..... 665

[P banksiana GW749263](http://www.ncbi.nlm.nih.gov/entrez/query.fcgi?cmd=Retrieve&db=Nucleotide&list_uids=292528791&dopt=GenBank&RID=ZSASR36A112&log$=nuclalign&blast_rank=0) 5 ...............................................T.........G.C-..... 69

[Radiata FE522359](http://www.ncbi.nlm.nih.gov/entrez/query.fcgi?cmd=Retrieve&db=Nucleotide&list_uids=222825747&dopt=GenBank&RID=ZSASR36A112&log$=nuclalign&blast_rank=0) 778 ........C........T..A..G.............................................................................T.........G.CC..... 659

[P taeda DR118268](http://www.ncbi.nlm.nih.gov/entrez/query.fcgi?cmd=Retrieve&db=Nucleotide&list_uids=67706578&dopt=GenBank&RID=ZSASR36A112&log$=nuclalign&blast_rank=0) 1 ....T.........G..-..... 22

**EF1a F2========================>**

<=======================**EF1aR1 <==========...EF1a R2**

[Sitka EF677538](http://www.ncbi.nlm.nih.gov/nuccore/148908490) 1202 **CATGACCAAGGTTGACAGGCGTTCT**GGCAAGGAACTGGAGAAGGAACCCAAGTTTCTTAAGAATGGAGATGCCGGGTTTGTTAAGATGATACCAACCAAACCCATGGTT**GTGGAAACCTT** 1321

[Sitka EF084956](http://www.ncbi.nlm.nih.gov/entrez/query.fcgi?cmd=Retrieve&db=Nucleotide&list_uids=116786851&dopt=GenBank&RID=ZSASR36A112&log$=nuclalign&blast_rank=0) 1190 ........................................................................................................................ 1309

[Sitka EF085539](http://www.ncbi.nlm.nih.gov/entrez/query.fcgi?cmd=Retrieve&db=Nucleotide&list_uids=116788344&dopt=GenBank&RID=ZSASR36A112&log$=nuclalign&blast_rank=0) 1199 ........................................................................................................................ 1318

[White spruce BT102965](http://www.ncbi.nlm.nih.gov/entrez/query.fcgi?cmd=Retrieve&db=Nucleotide&list_uids=270136013&dopt=GenBank&RID=ZSASR36A112&log$=nuclalign&blast_rank=0) 1182 ........................................................................................................................ 1301

[P abies AJ132534](http://www.ncbi.nlm.nih.gov/entrez/query.fcgi?cmd=Retrieve&db=Nucleotide&list_uids=12580860&dopt=GenBank&RID=ZSASR36A112&log$=nuclalign&blast_rank=0) 1080 ........................................................................................................................ 1199

[P menziesii AY832581](http://www.ncbi.nlm.nih.gov/entrez/query.fcgi?cmd=Retrieve&db=Nucleotide&list_uids=56481438&dopt=GenBank&RID=ZSASR36A112&log$=nuclalign&blast_rank=0) 491 .................................G....................C........C........T........C........T...........A................. 610

[P sylvestris EG418150](http://www.ncbi.nlm.nih.gov/entrez/query.fcgi?cmd=Retrieve&db=Nucleotide&list_uids=116375558&dopt=GenBank&RID=ZSASR36A112&log$=nuclalign&blast_rank=0) 664 ........................C........GT...................C.................T..A..............T........G...........C........ 545

[P pinaster CT581497](http://www.ncbi.nlm.nih.gov/entrez/query.fcgi?cmd=Retrieve&db=Nucleotide&list_uids=90039770&dopt=GenBank&RID=ZSASR36A112&log$=nuclalign&blast_rank=0) 664 .................................G...........G..........................T..A..............T....................C.....T.. 545

[P banksiana GW749263](http://www.ncbi.nlm.nih.gov/entrez/query.fcgi?cmd=Retrieve&db=Nucleotide&list_uids=292528791&dopt=GenBank&RID=ZSASR36A112&log$=nuclalign&blast_rank=0) 70 TCA...T.................C........G...........G........C.................T..A..............T....................C........ 189

[Radiata FE522359](http://www.ncbi.nlm.nih.gov/entrez/query.fcgi?cmd=Retrieve&db=Nucleotide&list_uids=222825747&dopt=GenBank&RID=ZSASR36A112&log$=nuclalign&blast_rank=0) 658 TCA...T.................C........G...........G........C.................T..A..............T....................C........ 539

[P taeda DR118268](http://www.ncbi.nlm.nih.gov/entrez/query.fcgi?cmd=Retrieve&db=Nucleotide&list_uids=67706578&dopt=GenBank&RID=ZSASR36A112&log$=nuclalign&blast_rank=0) 23 TCA.....................C........G...........G........C.................T..A..............T............................. 142

**...==========EF1a R2**

**EF1aF4==========================> <**============================**EF1aR4 EF1aF5**=====...

[Sitka EF677538](http://www.ncbi.nlm.nih.gov/nuccore/148908490) 1322 **CGCTGAGTATCCT**CCATTGGGTCGTTTTGCTGTGAGGGACATGCGTCAGACGGTTGCTGTAGGAGTCATCAAAGCTGTTGAAAAGAAGGACCCTACTGGTGCAAAGGTCACCAAGGCAGC 1441

[Sitka EF084956](http://www.ncbi.nlm.nih.gov/entrez/query.fcgi?cmd=Retrieve&db=Nucleotide&list_uids=116786851&dopt=GenBank&RID=ZSASR36A112&log$=nuclalign&blast_rank=0) 1310 .............................................................................................C.......................... 1429

[Sitka EF085539](http://www.ncbi.nlm.nih.gov/entrez/query.fcgi?cmd=Retrieve&db=Nucleotide&list_uids=116788344&dopt=GenBank&RID=ZSASR36A112&log$=nuclalign&blast_rank=0) 1319 ..................................G..................................................................................... 1438

[White spruce BT102965](http://www.ncbi.nlm.nih.gov/entrez/query.fcgi?cmd=Retrieve&db=Nucleotide&list_uids=270136013&dopt=GenBank&RID=ZSASR36A112&log$=nuclalign&blast_rank=0) 1302 ........................................................................................................................ 1421

[P abies AJ132534](http://www.ncbi.nlm.nih.gov/entrez/query.fcgi?cmd=Retrieve&db=Nucleotide&list_uids=12580860&dopt=GenBank&RID=ZSASR36A112&log$=nuclalign&blast_rank=0) 1200 ........................................................................................................................ 1319

[P menziesii AY832581](http://www.ncbi.nlm.nih.gov/entrez/query.fcgi?cmd=Retrieve&db=Nucleotide&list_uids=56481438&dopt=GenBank&RID=ZSASR36A112&log$=nuclalign&blast_rank=0) 611 T..C..........................A.............................T........T.................................................. 730

[P sylvestris EG418150](http://www.ncbi.nlm.nih.gov/entrez/query.fcgi?cmd=Retrieve&db=Nucleotide&list_uids=116375558&dopt=GenBank&RID=ZSASR36A112&log$=nuclalign&blast_rank=0) 544 T.............................A..C........................................................A....G............T........... 425

[P pinaster CT581497](http://www.ncbi.nlm.nih.gov/entrez/query.fcgi?cmd=Retrieve&db=Nucleotide&list_uids=90039770&dopt=GenBank&RID=ZSASR36A112&log$=nuclalign&blast_rank=0) 544 T..A..........................A..C........................................................A....G............T........... 425

[P banksiana GW749263](http://www.ncbi.nlm.nih.gov/entrez/query.fcgi?cmd=Retrieve&db=Nucleotide&list_uids=292528791&dopt=GenBank&RID=ZSASR36A112&log$=nuclalign&blast_rank=0) 190 T.............................A..C.................A.........................................A..............T........... 309

[Radiata FE522359](http://www.ncbi.nlm.nih.gov/entrez/query.fcgi?cmd=Retrieve&db=Nucleotide&list_uids=222825747&dopt=GenBank&RID=ZSASR36A112&log$=nuclalign&blast_rank=0) 538 T.............................A..C.................A.........................................A..............T........... 419

[P taeda DR118268](http://www.ncbi.nlm.nih.gov/entrez/query.fcgi?cmd=Retrieve&db=Nucleotide&list_uids=67706578&dopt=GenBank&RID=ZSASR36A112&log$=nuclalign&blast_rank=0) 143 T.............................A..C.................A......................................A..A.G............T........... 262

**EF1aF5...**====================> Stp ~370bp from the polyA

[Sitka EF677538](http://www.ncbi.nlm.nih.gov/nuccore/148908490) 1442 TGCCAAAAAGAAGTGAATGTTCAAGCAAAG**TGA**TCGGGAATGGAGAAATGCTTGCTGCATTTTGTGTGCTTGC------ATGCTTCTCTATTTTCCTGATGGTATGTTAGTAGAGTTAGA 1555

[Sitka EF084956](http://www.ncbi.nlm.nih.gov/entrez/query.fcgi?cmd=Retrieve&db=Nucleotide&list_uids=116786851&dopt=GenBank&RID=ZSASR36A112&log$=nuclalign&blast_rank=0) 1430 .........................................................................------......................................... 1543

[Sitka EF085539](http://www.ncbi.nlm.nih.gov/entrez/query.fcgi?cmd=Retrieve&db=Nucleotide&list_uids=116788344&dopt=GenBank&RID=ZSASR36A112&log$=nuclalign&blast_rank=0) 1439 .........................................................................------......................................... 1552

[White spruce BT102965](http://www.ncbi.nlm.nih.gov/entrez/query.fcgi?cmd=Retrieve&db=Nucleotide&list_uids=270136013&dopt=GenBank&RID=ZSASR36A112&log$=nuclalign&blast_rank=0) 1422 .........................................................................------......................................... 1535

[P abies AJ132534](http://www.ncbi.nlm.nih.gov/entrez/query.fcgi?cmd=Retrieve&db=Nucleotide&list_uids=12580860&dopt=GenBank&RID=ZSASR36A112&log$=nuclalign&blast_rank=0) 1320 .........................................................................------......................................... 1433

[P menziesii AY832581](http://www.ncbi.nlm.nih.gov/entrez/query.fcgi?cmd=Retrieve&db=Nucleotide&list_uids=56481438&dopt=GenBank&RID=ZSASR36A112&log$=nuclalign&blast_rank=0) 731 ...T..G...........A..G..TAC.......T..........G.G.TTG.A.C.....AC.C........------..CT....................................T 844

[P sylvestris EG418150](http://www.ncbi.nlm.nih.gov/entrez/query.fcgi?cmd=Retrieve&db=Nucleotide&list_uids=116375558&dopt=GenBank&RID=ZSASR36A112&log$=nuclalign&blast_rank=0) 424 A.....................C...........T..........G.T...G..........C........C.ATGCTT.......A.........C.G.A..................T 305

[P pinaster CT581497](http://www.ncbi.nlm.nih.gov/entrez/query.fcgi?cmd=Retrieve&db=Nucleotide&list_uids=90039770&dopt=GenBank&RID=ZSASR36A112&log$=nuclalign&blast_rank=0) 424 A.....................C...........T..........G.T...G..........C........C.ATGCTT.......A.........C...A..................T 305

[P banksiana GW749263](http://www.ncbi.nlm.nih.gov/entrez/query.fcgi?cmd=Retrieve&db=Nucleotide&list_uids=292528791&dopt=GenBank&RID=ZSASR36A112&log$=nuclalign&blast_rank=0) 310 A....................TC...........T..........G.T...G..........C........C.ATGCTT.......A.........C...AT.................T 429

[Radiata FE522359](http://www.ncbi.nlm.nih.gov/entrez/query.fcgi?cmd=Retrieve&db=Nucleotide&list_uids=222825747&dopt=GenBank&RID=ZSASR36A112&log$=nuclalign&blast_rank=0) 418 A....................TC...........T..........G.T...G..........C........C.ATGCTT.......A.........C...AT.................T 299

[P taeda DR118268](http://www.ncbi.nlm.nih.gov/entrez/query.fcgi?cmd=Retrieve&db=Nucleotide&list_uids=67706578&dopt=GenBank&RID=ZSASR36A112&log$=nuclalign&blast_rank=0) 263 A....................TC...........T....G.....G.T...G..........C........C.ATGCTT.......A.........A...AT.................T 382

**EF1aF3============================>**

<======================**EF1aR5** ~220bp from the polyA **<==========...EF1a R3**

[Sitka EF677538](http://www.ncbi.nlm.nih.gov/nuccore/148908490) 1556 TTGTCAAATACAGAATTGGGTGCT**TGATCGGCGGTGGCAGAGTTTACATTATT**TTCTAGTAAATACTCTTTAGTGGT----TTAGGGCAAA-TTGA-TTGAGATTTCGTAGTTGTGGGGA 1669

[Sitka EF084956](http://www.ncbi.nlm.nih.gov/entrez/query.fcgi?cmd=Retrieve&db=Nucleotide&list_uids=116786851&dopt=GenBank&RID=ZSASR36A112&log$=nuclalign&blast_rank=0) 1544 .............................................................................----..........-....-....................... 1657

[Sitka EF085539](http://www.ncbi.nlm.nih.gov/entrez/query.fcgi?cmd=Retrieve&db=Nucleotide&list_uids=116788344&dopt=GenBank&RID=ZSASR36A112&log$=nuclalign&blast_rank=0) 1553 .............................................................................----..........-....-....................... 1666

[White spruce BT102965](http://www.ncbi.nlm.nih.gov/entrez/query.fcgi?cmd=Retrieve&db=Nucleotide&list_uids=270136013&dopt=GenBank&RID=ZSASR36A112&log$=nuclalign&blast_rank=0) 1536 .............................................................................----..........-....-....................... 1649

[P abies AJ132534](http://www.ncbi.nlm.nih.gov/entrez/query.fcgi?cmd=Retrieve&db=Nucleotide&list_uids=12580860&dopt=GenBank&RID=ZSASR36A112&log$=nuclalign&blast_rank=0) 1434 ..A..........................................................................----..........-....-....................... 1547

[P menziesii AY832581](http://www.ncbi.nlm.nih.gov/entrez/query.fcgi?cmd=Retrieve&db=Nucleotide&list_uids=56481438&dopt=GenBank&RID=ZSASR36A112&log$=nuclalign&blast_rank=0) 845 ..A......G..............................................C.....G...........A.C----.........G-....-.............T..TC..... 958

[P sylvestris EG418150](http://www.ncbi.nlm.nih.gov/entrez/query.fcgi?cmd=Retrieve&db=Nucleotide&list_uids=116375558&dopt=GenBank&RID=ZSASR36A112&log$=nuclalign&blast_rank=0) 304 ...C.....G...........T.................T.AG........................T....A....TACA......T...G....T..A..............C..... 185

[P pinaster CT581497](http://www.ncbi.nlm.nih.gov/entrez/query.fcgi?cmd=Retrieve&db=Nucleotide&list_uids=90039770&dopt=GenBank&RID=ZSASR36A112&log$=nuclalign&blast_rank=0) 304 ..TC.....G...........T.................T..G........................T....A....TACA.....CT...G....T..A..............C..... 185

[P banksiana GW749263](http://www.ncbi.nlm.nih.gov/entrez/query.fcgi?cmd=Retrieve&db=Nucleotide&list_uids=292528791&dopt=GenBank&RID=ZSASR36A112&log$=nuclalign&blast_rank=0) 430 ...C.....G...........T.................T.AG.......G................T....A....TACA......T...G....T..A..............C..... 549

[Radiata FE522359](http://www.ncbi.nlm.nih.gov/entrez/query.fcgi?cmd=Retrieve&db=Nucleotide&list_uids=222825747&dopt=GenBank&RID=ZSASR36A112&log$=nuclalign&blast_rank=0) 298 ...C.....G...........T.................T.AG.......G................T....A....TACA......T...G....T..A..............C..... 179

[P taeda DR118268](http://www.ncbi.nlm.nih.gov/entrez/query.fcgi?cmd=Retrieve&db=Nucleotide&list_uids=67706578&dopt=GenBank&RID=ZSASR36A112&log$=nuclalign&blast_rank=0) 383 ...C.....G...........T.................T.AG.......G................T....A....TACA......T...G....T..A..............C..... 502

**...=================EF1a R3**

[Sitka EF677538](http://www.ncbi.nlm.nih.gov/nuccore/148908490) 1670 AATCATCAGTTTGTCTGCACTGCTAGGAAATCTATGCAGTTG----GAGT------GAGGAAGGGTATTAATAATTTCTGTTACT------CTGTTTTAT-TTTTAAAGCTTGTATTGCC 1772

[Sitka EF084956](http://www.ncbi.nlm.nih.gov/entrez/query.fcgi?cmd=Retrieve&db=Nucleotide&list_uids=116786851&dopt=GenBank&RID=ZSASR36A112&log$=nuclalign&blast_rank=0) 1658 ..........................................----....------.............................------.........-................... 1760

[Sitka EF085539](http://www.ncbi.nlm.nih.gov/entrez/query.fcgi?cmd=Retrieve&db=Nucleotide&list_uids=116788344&dopt=GenBank&RID=ZSASR36A112&log$=nuclalign&blast_rank=0) 1667 .........-................................----....------.............................------.........-................... 1768

[White spruce BT102965](http://www.ncbi.nlm.nih.gov/entrez/query.fcgi?cmd=Retrieve&db=Nucleotide&list_uids=270136013&dopt=GenBank&RID=ZSASR36A112&log$=nuclalign&blast_rank=0) 1650 ..........................................----....------.............................------........--..............-.... 1750

[P abies AJ132534](http://www.ncbi.nlm.nih.gov/entrez/query.fcgi?cmd=Retrieve&db=Nucleotide&list_uids=12580860&dopt=GenBank&RID=ZSASR36A112&log$=nuclalign&blast_rank=0) 1548 ...........................-..............----....------.............................------.........-................... 1649

[P menziesii AY832581](http://www.ncbi.nlm.nih.gov/entrez/query.fcgi?cmd=Retrieve&db=Nucleotide&list_uids=56481438&dopt=GenBank&RID=ZSASR36A112&log$=nuclalign&blast_rank=0) 959 ...................T.....------A..........----....GTGAGG........A....................------.......T.A.......T.A......... 1062

[P sylvestris EG418150](http://www.ncbi.nlm.nih.gov/entrez/query.fcgi?cmd=Retrieve&db=Nucleotide&list_uids=116375558&dopt=GenBank&RID=ZSASR36A112&log$=nuclalign&blast_rank=0) 184 .........................................T----...A------.....T...C.............A...--------...G....---.....T............ 86

[P pinaster CT581497](http://www.ncbi.nlm.nih.gov/entrez/query.fcgi?cmd=Retrieve&db=Nucleotide&list_uids=90039770&dopt=GenBank&RID=ZSASR36A112&log$=nuclalign&blast_rank=0) 184 ......G................A..................GATT...G------.....T...C.............A.....GGTTTA...G.....-......T.T.......... 72

[P banksiana GW749263](http://www.ncbi.nlm.nih.gov/entrez/query.fcgi?cmd=Retrieve&db=Nucleotide&list_uids=292528791&dopt=GenBank&RID=ZSASR36A112&log$=nuclalign&blast_rank=0) 550 ..........................................GATT...A------.....T...C.............A...--------...G....---.....T.T.......C.. 652

[Radiata FE522359](http://www.ncbi.nlm.nih.gov/entrez/query.fcgi?cmd=Retrieve&db=Nucleotide&list_uids=222825747&dopt=GenBank&RID=ZSASR36A112&log$=nuclalign&blast_rank=0) 178 ..........................................GATT...A------.....T...C.............A...--------...G....---.....T.T.......C.. 76

[P taeda DR118268](http://www.ncbi.nlm.nih.gov/entrez/query.fcgi?cmd=Retrieve&db=Nucleotide&list_uids=67706578&dopt=GenBank&RID=ZSASR36A112&log$=nuclalign&blast_rank=0) 503 ..........................................GATT...A------.....T...C.............A...--------...G....---.....T.T.......C.. 605

## YLS8

[Sitka EF083403](http://www.ncbi.nlm.nih.gov/nucleotide/116782986) 121 CATTCGCTTCGGCCATGACTGGGATGAAACATGTATGCAGATGGACGAAGTGCTATCATCCGTTGCAGAAACCATCAAGAATTTCGCAGTGATATACCTGGTGGACATAACAGAGGTGCC 240

[Sitka EF083219](http://www.ncbi.nlm.nih.gov/entrez/query.fcgi?cmd=Retrieve&db=Nucleotide&list_uids=116782602&dopt=GenBank&RID=0AKKBAG8114&log$=nuclalign&blast_rank=0) 380 ........................C.................................................................T............................. 499

[White EX351702](http://www.ncbi.nlm.nih.gov/entrez/query.fcgi?cmd=Retrieve&db=Nucleotide&list_uids=157550471&dopt=GenBank&RID=0AKKBAG8114&log$=nuclalign&blast_rank=0) 152 ........................C......................................C.....................................................A.. 271

[Taeda CF670220](http://www.ncbi.nlm.nih.gov/entrez/query.fcgi?cmd=Retrieve&db=Nucleotide&list_uids=37567613&dopt=GenBank&RID=0AKKBAG8114&log$=nuclalign&blast_rank=0) 174 ..................T.....C.............................G.....T...........A......................................G........ 293

[Abies AM171073](http://www.ncbi.nlm.nih.gov/entrez/query.fcgi?cmd=Retrieve&db=Nucleotide&list_uids=90030556&dopt=GenBank&RID=0AKKBAG8114&log$=nuclalign&blast_rank=0) 104 ........................C......................................G........................................................ 223

[Abies GT884497](http://www.ncbi.nlm.nih.gov/entrez/query.fcgi?cmd=Retrieve&db=Nucleotide&list_uids=280978711&dopt=GenBank&RID=0AKKBAG8114&log$=nuclalign&blast_rank=0) 163 ........................C............................................................................................... 282

[Banksiana GW761594](http://www.ncbi.nlm.nih.gov/entrez/query.fcgi?cmd=Retrieve&db=Nucleotide&list_uids=292545837&dopt=GenBank&RID=0AKKBAG8114&log$=nuclalign&blast_rank=0) 125 ..................T.....C.............................G.....T...........A......................................G........ 244

[Banksiana GW747245](http://www.ncbi.nlm.nih.gov/entrez/query.fcgi?cmd=Retrieve&db=Nucleotide&list_uids=292531123&dopt=GenBank&RID=0AKKBAG8114&log$=nuclalign&blast_rank=0) 125 ........................C.............................G.....T...........T......................................G........ 244

[Concorta GT267053](http://www.ncbi.nlm.nih.gov/entrez/query.fcgi?cmd=Retrieve&db=Nucleotide&list_uids=262157666&dopt=GenBank&RID=0AKKBAG8114&log$=nuclalign&blast_rank=0) 119 ........................C.............................G.....T...........T......................................G........ 238

[Pinaster CR393038](http://www.ncbi.nlm.nih.gov/entrez/query.fcgi?cmd=Retrieve&db=Nucleotide&list_uids=47033601&dopt=GenBank&RID=0AKKBAG8114&log$=nuclalign&blast_rank=0) 99 ........................C.............................G.....T..................................................G........ 218

**YLS8 F4===========================> YLS8 F3======================> YLS8 F1==========...**

[Sitka EF083403](http://www.ncbi.nlm.nih.gov/nucleotide/116782986) 241 TGATTTCAACACCATGTACGAGCTGTATGACCCGTCTACTGTCATGTTTTTCTTCCGCAATAAGCATATAATGATAGACTTGGGGACGGGGAACAACAATAAGATCAACTGGGCTATGAA 360

[Sitka EF083219](http://www.ncbi.nlm.nih.gov/entrez/query.fcgi?cmd=Retrieve&db=Nucleotide&list_uids=116782602&dopt=GenBank&RID=0AKKBAG8114&log$=nuclalign&blast_rank=0) 500 ........................................................................................................................ 619

[White EX351702](http://www.ncbi.nlm.nih.gov/entrez/query.fcgi?cmd=Retrieve&db=Nucleotide&list_uids=157550471&dopt=GenBank&RID=0AKKBAG8114&log$=nuclalign&blast_rank=0) 272 ..........................................................G............................................................. 391

[Taeda CF670220](http://www.ncbi.nlm.nih.gov/entrez/query.fcgi?cmd=Retrieve&db=Nucleotide&list_uids=37567613&dopt=GenBank&RID=0AKKBAG8114&log$=nuclalign&blast_rank=0) 294 .....................A........................................................T.......................A...........C..... 413

[Abies AM171073](http://www.ncbi.nlm.nih.gov/entrez/query.fcgi?cmd=Retrieve&db=Nucleotide&list_uids=90030556&dopt=GenBank&RID=0AKKBAG8114&log$=nuclalign&blast_rank=0) 224 ........................................................................................................................ 343

[Abies GT884497](http://www.ncbi.nlm.nih.gov/entrez/query.fcgi?cmd=Retrieve&db=Nucleotide&list_uids=280978711&dopt=GenBank&RID=0AKKBAG8114&log$=nuclalign&blast_rank=0) 283 ........................................................................................................................ 402

[Banksiana GW761594](http://www.ncbi.nlm.nih.gov/entrez/query.fcgi?cmd=Retrieve&db=Nucleotide&list_uids=292545837&dopt=GenBank&RID=0AKKBAG8114&log$=nuclalign&blast_rank=0) 245 .....................A........................................................T.......................A...........C..... 364

[Banksiana GW747245](http://www.ncbi.nlm.nih.gov/entrez/query.fcgi?cmd=Retrieve&db=Nucleotide&list_uids=292531123&dopt=GenBank&RID=0AKKBAG8114&log$=nuclalign&blast_rank=0) 245 .....................A.........................................A..............T.......................A................. 364

[Concorta GT267053](http://www.ncbi.nlm.nih.gov/entrez/query.fcgi?cmd=Retrieve&db=Nucleotide&list_uids=262157666&dopt=GenBank&RID=0AKKBAG8114&log$=nuclalign&blast_rank=0) 239 .....................A.........................................A..............T.......................A................. 358

[Pinaster CR393038](http://www.ncbi.nlm.nih.gov/entrez/query.fcgi?cmd=Retrieve&db=Nucleotide&list_uids=47033601&dopt=GenBank&RID=0AKKBAG8114&log$=nuclalign&blast_rank=0) 219 .........T...........A.........................................A..............T.......................A................. 338

**YLS8 F2=========================>**

**YLS8 F1...=============> <====================YLS8 R1** STP ~310bp from polyA site

[Sitka EF083403](http://www.ncbi.nlm.nih.gov/nucleotide/116782986) 361 GGACAAGCAGGAGTTCATTGATATTATTGAGACTGTGTACAGAGGGGCCAGGAAGGGCAGGGGTC**TGGTCATTGCCCCCAAGGATTATTCT**ACCAAGTACCGATAT**TAG**GTAGATAA-CA 479

[Sitka EF083219](http://www.ncbi.nlm.nih.gov/entrez/query.fcgi?cmd=Retrieve&db=Nucleotide&list_uids=116782602&dopt=GenBank&RID=0AKKBAG8114&log$=nuclalign&blast_rank=0) 620 ..........................................................................................**C**.................A........-.. 738

[White EX351702](http://www.ncbi.nlm.nih.gov/entrez/query.fcgi?cmd=Retrieve&db=Nucleotide&list_uids=157550471&dopt=GenBank&RID=0AKKBAG8114&log$=nuclalign&blast_rank=0) 392 ..........................................................................................C..........................-.. 510

[Taeda CF670220](http://www.ncbi.nlm.nih.gov/entrez/query.fcgi?cmd=Retrieve&db=Nucleotide&list_uids=37567613&dopt=GenBank&RID=0AKKBAG8114&log$=nuclalign&blast_rank=0) 414 A................................C.......................................................................C...........-.. 532

[Abies AM171073](http://www.ncbi.nlm.nih.gov/entrez/query.fcgi?cmd=Retrieve&db=Nucleotide&list_uids=90030556&dopt=GenBank&RID=0AKKBAG8114&log$=nuclalign&blast_rank=0) 344 ...........................................................................T..............C....................C.....-.. 462

[Abies GT884497](http://www.ncbi.nlm.nih.gov/entrez/query.fcgi?cmd=Retrieve&db=Nucleotide&list_uids=280978711&dopt=GenBank&RID=0AKKBAG8114&log$=nuclalign&blast_rank=0) 403 ..................................................C...T....C..............................C.................C.C......C.. 522

[Banksiana GW761594](http://www.ncbi.nlm.nih.gov/entrez/query.fcgi?cmd=Retrieve&db=Nucleotide&list_uids=292545837&dopt=GenBank&RID=0AKKBAG8114&log$=nuclalign&blast_rank=0) 365 A................................C.......................................................................C...........-.. 483

[Banksiana GW747245](http://www.ncbi.nlm.nih.gov/entrez/query.fcgi?cmd=Retrieve&db=Nucleotide&list_uids=292531123&dopt=GenBank&RID=0AKKBAG8114&log$=nuclalign&blast_rank=0) 365 ............A...........C........C.......................................................................C...........-.. 483

[Concorta GT267053](http://www.ncbi.nlm.nih.gov/entrez/query.fcgi?cmd=Retrieve&db=Nucleotide&list_uids=262157666&dopt=GenBank&RID=0AKKBAG8114&log$=nuclalign&blast_rank=0) 359 ............A...........C........C.......................................................................C...........-.. 477

[Pinaster CR393038](http://www.ncbi.nlm.nih.gov/entrez/query.fcgi?cmd=Retrieve&db=Nucleotide&list_uids=47033601&dopt=GenBank&RID=0AKKBAG8114&log$=nuclalign&blast_rank=0) 339 ............A...........C........C...................................A...................................C...........-.. 457

## ACT2

[Sitka GT121097](http://www.ncbi.nlm.nih.gov/nucest/260191095) 181 GCCTTCTCTAATTGGGATGGAAGCTGCTGGTATCCATGAGACAACATACAACTCCATCATGAAGTGTGACGTGGATATTAGAAAGGATCTATATGGAAACATTGTGCTTAGTGGTGGGTC 300

[White DR558688](http://www.ncbi.nlm.nih.gov/entrez/query.fcgi?cmd=Retrieve&db=Nucleotide&list_uids=70622895&dopt=GenBank&RID=07P7UPAA11R&log$=nuclalign&blast_rank=0) 648 ........................................................................................................................ 529

[Banksiana GW773484](http://www.ncbi.nlm.nih.gov/entrez/query.fcgi?cmd=Retrieve&db=Nucleotide&list_uids=292554968&dopt=GenBank&RID=07P7UPAA11R&log$=nuclalign&blast_rank=0) 645 .......T...................A.................G.......................T.................................................. 526

[Banksiana GW747595](http://www.ncbi.nlm.nih.gov/entrez/query.fcgi?cmd=Retrieve&db=Nucleotide&list_uids=292531760&dopt=GenBank&RID=07P7UPAA11R&log$=nuclalign&blast_rank=0) 794 .......T...................A.................G.......................T.................................................. 675

[Radiata FE521942](http://www.ncbi.nlm.nih.gov/entrez/query.fcgi?cmd=Retrieve&db=Nucleotide&list_uids=222830041&dopt=GenBank&RID=07P7UPAA11R&log$=nuclalign&blast_rank=0) 256 .......T...................A.................G.......................T.................................................. 375

[Radiata FE519638](http://www.ncbi.nlm.nih.gov/entrez/query.fcgi?cmd=Retrieve&db=Nucleotide&list_uids=222829869&dopt=GenBank&RID=07P7UPAA11R&log$=nuclalign&blast_rank=0) 678 ...A...T...................A.................G.......................T.................................................. 559

[Taeda DR022170](http://www.ncbi.nlm.nih.gov/entrez/query.fcgi?cmd=Retrieve&db=Nucleotide&list_uids=66744540&dopt=GenBank&RID=07P7UPAA11R&log$=nuclalign&blast_rank=0) 192 .......T...................A.................G.......................T.................................................. 311

[Taeda CO164516](http://www.ncbi.nlm.nih.gov/entrez/query.fcgi?cmd=Retrieve&db=Nucleotide&list_uids=48935057&dopt=GenBank&RID=07P7UPAA11R&log$=nuclalign&blast_rank=0) 625 .......T...................A.................G.......................T.................................................. 506

[Taeda CO365759](http://www.ncbi.nlm.nih.gov/entrez/query.fcgi?cmd=Retrieve&db=Nucleotide&list_uids=49447076&dopt=GenBank&RID=07P7UPAA11R&log$=nuclalign&blast_rank=0) 141 .......T.......A...........A.................G.......................T.................................................. 260

[Pinaster BX249310](http://www.ncbi.nlm.nih.gov/entrez/query.fcgi?cmd=Retrieve&db=Nucleotide&list_uids=28507588&dopt=GenBank&RID=07P7UPAA11R&log$=nuclalign&blast_rank=0) 13 .......T...................A.................T.......................T.................................................. 132

[Abies AM169069](http://www.ncbi.nlm.nih.gov/entrez/query.fcgi?cmd=Retrieve&db=Nucleotide&list_uids=89956108&dopt=GenBank&RID=07P7UPAA11R&log$=nuclalign&blast_rank=0) 140 ........................................................................................................................ 259

**Act2 F1===========================> <====...ACT2 R1**

[Sitka GT121097](http://www.ncbi.nlm.nih.gov/nucest/260191095) 301 TACTATGTTTCCTGGTATTGCTGACCGTATGAGCAAGGAAATCACTGCACTGGCTCCTAGCAGCATGAAAATCAAGGTCGTTGCACCTCCAGAGAGGAAGTACAGTGTCTGGATTGGAGG 420

[White DR558688](http://www.ncbi.nlm.nih.gov/entrez/query.fcgi?cmd=Retrieve&db=Nucleotide&list_uids=70622895&dopt=GenBank&RID=07P7UPAA11R&log$=nuclalign&blast_rank=0) 528 ........................................................................................................................ 409

[Norway AM169069](http://www.ncbi.nlm.nih.gov/entrez/query.fcgi?cmd=Retrieve&db=Nucleotide&list_uids=89956108&dopt=GenBank&RID=07P7UPAA11R&log$=nuclalign&blast_rank=0) 260 ........................................................................................................................ 379

[Banksiana GW773484](http://www.ncbi.nlm.nih.gov/entrez/query.fcgi?cmd=Retrieve&db=Nucleotide&list_uids=292554968&dopt=GenBank&RID=07P7UPAA11R&log$=nuclalign&blast_rank=0) 525 C............................................C...........C....................G......................................... 406

[Banksiana GW747595](http://www.ncbi.nlm.nih.gov/entrez/query.fcgi?cmd=Retrieve&db=Nucleotide&list_uids=292531760&dopt=GenBank&RID=07P7UPAA11R&log$=nuclalign&blast_rank=0) 674 C............................................C...........C....................G......................................... 555

[Radiata FE521942](http://www.ncbi.nlm.nih.gov/entrez/query.fcgi?cmd=Retrieve&db=Nucleotide&list_uids=222830041&dopt=GenBank&RID=07P7UPAA11R&log$=nuclalign&blast_rank=0) 376 C............................................C...........C....................G......................................... 495

[Radiata FE519638](http://www.ncbi.nlm.nih.gov/entrez/query.fcgi?cmd=Retrieve&db=Nucleotide&list_uids=222829869&dopt=GenBank&RID=07P7UPAA11R&log$=nuclalign&blast_rank=0) 558 C............................................C...........C....................G......................................... 439

[Taeda DR022170](http://www.ncbi.nlm.nih.gov/entrez/query.fcgi?cmd=Retrieve&db=Nucleotide&list_uids=66744540&dopt=GenBank&RID=07P7UPAA11R&log$=nuclalign&blast_rank=0) 312 C............................................C...........C....................G......................................... 431

[Taeda CO164516](http://www.ncbi.nlm.nih.gov/entrez/query.fcgi?cmd=Retrieve&db=Nucleotide&list_uids=48935057&dopt=GenBank&RID=07P7UPAA11R&log$=nuclalign&blast_rank=0) 505 C............................................C...........C..................A.AAAAA..................................... 386

[Taeda CO365759](http://www.ncbi.nlm.nih.gov/entrez/query.fcgi?cmd=Retrieve&db=Nucleotide&list_uids=49447076&dopt=GenBank&RID=07P7UPAA11R&log$=nuclalign&blast_rank=0) 261 C............................................C...........C....................G......................................... 380

[Pinaster BX249310](http://www.ncbi.nlm.nih.gov/entrez/query.fcgi?cmd=Retrieve&db=Nucleotide&list_uids=28507588&dopt=GenBank&RID=07P7UPAA11R&log$=nuclalign&blast_rank=0) 133 C........................................................C....................G......................................... 252

**...========================ACT2 R1 <=======================*ACT2 R2*** |~300bp from the polyA tail

[Sitka GT121097](http://www.ncbi.nlm.nih.gov/nucest/260191095) 421 TTCTATTTTGGCATCTCTCAGCACATTCCAACAGATGTGGATTGCAAAGGCCGAGTATGATGAATCTGGTCCTTCCATCGTCCACAGAAAATGTTTT**TAA**TTTCGGTGCTTATATTACTT 540

[White DR558688](http://www.ncbi.nlm.nih.gov/entrez/query.fcgi?cmd=Retrieve&db=Nucleotide&list_uids=70622895&dopt=GenBank&RID=07P7UPAA11R&log$=nuclalign&blast_rank=0) 408 ........................................................................................................................ 289

[Norway AM169069](http://www.ncbi.nlm.nih.gov/entrez/query.fcgi?cmd=Retrieve&db=Nucleotide&list_uids=89956108&dopt=GenBank&RID=07P7UPAA11R&log$=nuclalign&blast_rank=0) 380 ........................................................................................................................ 499

[Banksiana GW773484](http://www.ncbi.nlm.nih.gov/entrez/query.fcgi?cmd=Retrieve&db=Nucleotide&list_uids=292554968&dopt=GenBank&RID=07P7UPAA11R&log$=nuclalign&blast_rank=0) 405 .............................................G.....T................................................G...A........G...... 286

[Banksiana GW747595](http://www.ncbi.nlm.nih.gov/entrez/query.fcgi?cmd=Retrieve&db=Nucleotide&list_uids=292531760&dopt=GenBank&RID=07P7UPAA11R&log$=nuclalign&blast_rank=0) 554 .............................................G.....T................................................G...A........G...... 435

[Radiata FE521942](http://www.ncbi.nlm.nih.gov/entrez/query.fcgi?cmd=Retrieve&db=Nucleotide&list_uids=222830041&dopt=GenBank&RID=07P7UPAA11R&log$=nuclalign&blast_rank=0) 496 ...................................................T................................................G...A........G...... 615

[Radiata FE519638](http://www.ncbi.nlm.nih.gov/entrez/query.fcgi?cmd=Retrieve&db=Nucleotide&list_uids=222829869&dopt=GenBank&RID=07P7UPAA11R&log$=nuclalign&blast_rank=0) 438 ...................................................T................................................G...A........G...... 319

[Taeda DR022170](http://www.ncbi.nlm.nih.gov/entrez/query.fcgi?cmd=Retrieve&db=Nucleotide&list_uids=66744540&dopt=GenBank&RID=07P7UPAA11R&log$=nuclalign&blast_rank=0) 432 ............................................T......T................................................G...A........G...... 551

[Taeda CO164516](http://www.ncbi.nlm.nih.gov/entrez/query.fcgi?cmd=Retrieve&db=Nucleotide&list_uids=48935057&dopt=GenBank&RID=07P7UPAA11R&log$=nuclalign&blast_rank=0) 385 ...................................................T................................................G...A........G...... 266

[Taeda CO365759](http://www.ncbi.nlm.nih.gov/entrez/query.fcgi?cmd=Retrieve&db=Nucleotide&list_uids=49447076&dopt=GenBank&RID=07P7UPAA11R&log$=nuclalign&blast_rank=0) 381 ...................................................T................................................G...A........G...... 500

[Pinaster BX249310](http://www.ncbi.nlm.nih.gov/entrez/query.fcgi?cmd=Retrieve&db=Nucleotide&list_uids=28507588&dopt=GenBank&RID=07P7UPAA11R&log$=nuclalign&blast_rank=0) 253 ...................................................T................................................G...A........G...... 372

[Sitka GT121097](http://www.ncbi.nlm.nih.gov/nucest/260191095) 541 TAGTGCTTTGTGAAGATGGGTATTCTAGAGAATGTTTGCTTGA---GATGGTGGCAATAGTGCTAAGGTCGAGATTCAGTTTATTTTTTATGCAAGGGTTTTAGAGAATATATCTGTGTA 657

[White DR558688](http://www.ncbi.nlm.nih.gov/entrez/query.fcgi?cmd=Retrieve&db=Nucleotide&list_uids=70622895&dopt=GenBank&RID=07P7UPAA11R&log$=nuclalign&blast_rank=0) 288 ...........................................---.......................................................................... 172

[Norway AM169069](http://www.ncbi.nlm.nih.gov/entrez/query.fcgi?cmd=Retrieve&db=Nucleotide&list_uids=89956108&dopt=GenBank&RID=07P7UPAA11R&log$=nuclalign&blast_rank=0) 500 .........A.................................---................ 558

[Banksiana GW773484](http://www.ncbi.nlm.nih.gov/entrez/query.fcgi?cmd=Retrieve&db=Nucleotide&list_uids=292554968&dopt=GenBank&RID=07P7UPAA11R&log$=nuclalign&blast_rank=0) 285 ......A.C..................................GAT..............................................................G...--.A.... 168

[Banksiana GW747595](http://www.ncbi.nlm.nih.gov/entrez/query.fcgi?cmd=Retrieve&db=Nucleotide&list_uids=292531760&dopt=GenBank&RID=07P7UPAA11R&log$=nuclalign&blast_rank=0) 434 ......A.C..................................GAT..............................................................G...--.A.... 317

[Radiata FE521942](http://www.ncbi.nlm.nih.gov/entrez/query.fcgi?cmd=Retrieve&db=Nucleotide&list_uids=222830041&dopt=GenBank&RID=07P7UPAA11R&log$=nuclalign&blast_rank=0) 616 ......A.C..................................GAT.......A............C.........................................G...--...... 733

[Radiata FE519638](http://www.ncbi.nlm.nih.gov/entrez/query.fcgi?cmd=Retrieve&db=Nucleotide&list_uids=222829869&dopt=GenBank&RID=07P7UPAA11R&log$=nuclalign&blast_rank=0) 318 ......A.C..................................GAT..............................................................G...--...... 201

[Taeda DR022170](http://www.ncbi.nlm.nih.gov/entrez/query.fcgi?cmd=Retrieve&db=Nucleotide&list_uids=66744540&dopt=GenBank&RID=07P7UPAA11R&log$=nuclalign&blast_rank=0) 552 ......A.C..........T.......................GAT..............................................................G...--...... 669

[Taeda CO164516](http://www.ncbi.nlm.nih.gov/entrez/query.fcgi?cmd=Retrieve&db=Nucleotide&list_uids=48935057&dopt=GenBank&RID=07P7UPAA11R&log$=nuclalign&blast_rank=0) 265 ......A.C..................................GAT..............................................................G...--....A. 148

[Taeda CO365759](http://www.ncbi.nlm.nih.gov/entrez/query.fcgi?cmd=Retrieve&db=Nucleotide&list_uids=49447076&dopt=GenBank&RID=07P7UPAA11R&log$=nuclalign&blast_rank=0) 501 ......A.C..................................GAT..............................................................G...--...... 618

[Pinaster BX249310](http://www.ncbi.nlm.nih.gov/entrez/query.fcgi?cmd=Retrieve&db=Nucleotide&list_uids=28507588&dopt=GenBank&RID=07P7UPAA11R&log$=nuclalign&blast_rank=0) 373 ......A.C..................................GAT.......................................................................... 492

## GAPDH

[Stika EF085448](http://www.ncbi.nlm.nih.gov/nucleotide/116788089) 1040 AAAGCTAACTGGAATGGCTTTCCGTGTCCCAACACCTAATGTCTCAGTCGTGGATCTGACATGTCGCCTAGTGAAACCAGCATCTTACGATGATGTAAAAGCAGCAATAAAGGCTGCATC 1159

[Sitka EF082132](http://www.ncbi.nlm.nih.gov/entrez/query.fcgi?cmd=Retrieve&db=Nucleotide&list_uids=116779991&dopt=GenBank&RID=0ADK5NMC112&log$=nuclalign&blast_rank=0) 395 ........................................................................................................................ 514

[White GE480409](http://www.ncbi.nlm.nih.gov/entrez/query.fcgi?cmd=Retrieve&db=Nucleotide&list_uids=211602282&dopt=GenBank&RID=0ADK5NMC112&log$=nuclalign&blast_rank=0) 539 .................................C......T.T............T.........................C.T..T................................T 420

[White GE480714](http://www.ncbi.nlm.nih.gov/entrez/query.fcgi?cmd=Retrieve&db=Nucleotide&list_uids=211603059&dopt=GenBank&RID=0ADK5NMC112&log$=nuclalign&blast_rank=0) 564 .......T....................T..C..........G...C..............C...T...............................................G...... 445

[Taeda DR163579](http://www.ncbi.nlm.nih.gov/entrez/query.fcgi?cmd=Retrieve&db=Nucleotide&list_uids=67961759&dopt=GenBank&RID=0ADK5NMC112&log$=nuclalign&blast_rank=0) 307 ...........................A........C...........T..C..............G..T........................A.............G.....C..... 426

[Concorta GT270539](http://www.ncbi.nlm.nih.gov/entrez/query.fcgi?cmd=Retrieve&db=Nucleotide&list_uids=262163269&dopt=GenBank&RID=0ADK5NMC112&log$=nuclalign&blast_rank=0) 198 ...........................A........C...........T..C..............T..T........................A.............G........... 317

[Pinaster CT577388](http://www.ncbi.nlm.nih.gov/entrez/query.fcgi?cmd=Retrieve&db=Nucleotide&list_uids=90044690&dopt=GenBank&RID=0ADK5NMC112&log$=nuclalign&blast_rank=0) 620 ...........................A........C...........T....................T.C......................A.............G........... 501

[Banksiana GW754757](http://www.ncbi.nlm.nih.gov/entrez/query.fcgi?cmd=Retrieve&db=Nucleotide&list_uids=292533044&dopt=GenBank&RID=0ADK5NMC112&log$=nuclalign&blast_rank=0) 615 ...........................A........C...........T..C..............T..T........................A.............G........... 496

**GAPDH F1===========================>**

[Stika EF085448](http://www.ncbi.nlm.nih.gov/nucleotide/116788089) 1160 TGAAGGGTCACTAAAAGGCATCCTTGGATACACTGATGAAGATGTCGTCTCGAATGATTTCATAGGTGATGAAAGGTCGAGTATCTTTGATTCTAAGGCTGGTATAGCCTTGAATACTGG 1279

[Sitka EF082132](http://www.ncbi.nlm.nih.gov/entrez/query.fcgi?cmd=Retrieve&db=Nucleotide&list_uids=116779991&dopt=GenBank&RID=0ADK5NMC112&log$=nuclalign&blast_rank=0) 515 ........................................................................................................................ 634

[White GE480409](http://www.ncbi.nlm.nih.gov/entrez/query.fcgi?cmd=Retrieve&db=Nucleotide&list_uids=211602282&dopt=GenBank&RID=0ADK5NMC112&log$=nuclalign&blast_rank=0) 419 .........C.....................C..................................G............G..T.T.......T........................... 300

[White GE480714](http://www.ncbi.nlm.nih.gov/entrez/query.fcgi?cmd=Retrieve&db=Nucleotide&list_uids=211603059&dopt=GenBank&RID=0ADK5NMC112&log$=nuclalign&blast_rank=0) 444 .........T................................G.................................................T.....G..........C.......... 325

[Taeda DR163579](http://www.ncbi.nlm.nih.gov/entrez/query.fcgi?cmd=Retrieve&db=Nucleotide&list_uids=67961759&dopt=GenBank&RID=0ADK5NMC112&log$=nuclalign&blast_rank=0) 427 ................................................T...........TG....C....C..................CG.....................G.T..AC 546

[Concorta GT270539](http://www.ncbi.nlm.nih.gov/entrez/query.fcgi?cmd=Retrieve&db=Nucleotide&list_uids=262163269&dopt=GenBank&RID=0ADK5NMC112&log$=nuclalign&blast_rank=0) 318 ................................................T...........TG....C....C..................CG.....................G.T..AC 437

[Pinaster CT577388](http://www.ncbi.nlm.nih.gov/entrez/query.fcgi?cmd=Retrieve&db=Nucleotide&list_uids=90044690&dopt=GenBank&RID=0ADK5NMC112&log$=nuclalign&blast_rank=0) 500 ..............................T.................T...........TG........TC...................G.....................G.T..AC 381

[Banksiana GW754757](http://www.ncbi.nlm.nih.gov/entrez/query.fcgi?cmd=Retrieve&db=Nucleotide&list_uids=292533044&dopt=GenBank&RID=0ADK5NMC112&log$=nuclalign&blast_rank=0) 495 ................................................T...........TG....C....C..................CG.....................G.T..AC 376

**<===========================GAPDH R1** STP ~260bp from polyA tail

[Stika EF085448](http://www.ncbi.nlm.nih.gov/nucleotide/116788089) 1280 ATTTGTGAAACTTGTTTCTTGGTATGACAATGAGTGGGGATACAGCAACCGAGTGGTGGACTTGATCTCACACATGGCTTTAGTTGATTCACGCAAA**TAG**AGATGTTTGTTTTTTGGAGT 1399

[Sitka EF082132](http://www.ncbi.nlm.nih.gov/entrez/query.fcgi?cmd=Retrieve&db=Nucleotide&list_uids=116779991&dopt=GenBank&RID=0ADK5NMC112&log$=nuclalign&blast_rank=0) 635 .............A........................................................................C................................. 754

[White GE480409](http://www.ncbi.nlm.nih.gov/entrez/query.fcgi?cmd=Retrieve&db=Nucleotide&list_uids=211602282&dopt=GenBank&RID=0ADK5NMC112&log$=nuclalign&blast_rank=0) 299 .................T......................................G.......T.T..C.C..................C............................. 180

[White GE480714](http://www.ncbi.nlm.nih.gov/entrez/query.fcgi?cmd=Retrieve&db=Nucleotide&list_uids=211603059&dopt=GenBank&RID=0ADK5NMC112&log$=nuclalign&blast_rank=0) 324 ...............................................C.....................T....C........A.................................... 205

[Taeda DR163579](http://www.ncbi.nlm.nih.gov/entrez/query.fcgi?cmd=Retrieve&db=Nucleotide&list_uids=67961759&dopt=GenBank&RID=0ADK5NMC112&log$=nuclalign&blast_rank=0) 547 ...C..................................................................................C.................TC..C.A......G.. 666

[Concorta GT270539](http://www.ncbi.nlm.nih.gov/entrez/query.fcgi?cmd=Retrieve&db=Nucleotide&list_uids=262163269&dopt=GenBank&RID=0ADK5NMC112&log$=nuclalign&blast_rank=0) 438 ...C..................................................................................C.................TC..C.A......... 557

[Pinaster CT577388](http://www.ncbi.nlm.nih.gov/entrez/query.fcgi?cmd=Retrieve&db=Nucleotide&list_uids=90044690&dopt=GenBank&RID=0ADK5NMC112&log$=nuclalign&blast_rank=0) 380 C......................................C..............................................C.................TC..C.A......... 261

[Banksiana GW754757](http://www.ncbi.nlm.nih.gov/entrez/query.fcgi?cmd=Retrieve&db=Nucleotide&list_uids=292533044&dopt=GenBank&RID=0ADK5NMC112&log$=nuclalign&blast_rank=0) 375 ...C..................................................................................C.................TC..C.A......... 256

## 46630

[Sitka EF678386](http://www.ncbi.nlm.nih.gov/nucleotide/148910125) 1135 TTTGGTGCAAAGATGTTTGCACTGGGAGTAGTAGTGAAGGTGCCAGTTCCAAAGCAAACGGCAAAGACGAATTTCCAGGTGACAACAGGTCGAGCCAAGTATAATGCAGCTATTGACTGC 1254

[White GO368075](http://www.ncbi.nlm.nih.gov/entrez/query.fcgi?cmd=Retrieve&db=Nucleotide&list_uids=225920080&dopt=GenBank&RID=0AVSPMRT112&log$=nuclalign&blast_rank=0) 431 ........................................................................................................................ 550

[Taeda DR057588](http://www.ncbi.nlm.nih.gov/entrez/query.fcgi?cmd=Retrieve&db=Nucleotide&list_uids=66981155&dopt=GenBank&RID=0AVSPMRT112&log$=nuclalign&blast_rank=0) 655 ....................GT....G................................A............................................................ 536

[Taeda DR178693](http://www.ncbi.nlm.nih.gov/entrez/query.fcgi?cmd=Retrieve&db=Nucleotide&list_uids=68085136&dopt=GenBank&RID=0AVSPMRT112&log$=nuclalign&blast_rank=0) 431 .....CAT...............A.....G..G.....A..A..T..G.....A.....A..T..AG.......T....................A..A..C......T.C.....T... 312

[Banksiana GW772395](http://www.ncbi.nlm.nih.gov/entrez/query.fcgi?cmd=Retrieve&db=Nucleotide&list_uids=292551601&dopt=GenBank&RID=0AVSPMRT112&log$=nuclalign&blast_rank=0) 273 ....................GT....G................................A............................................................ 392

**46630 F2=========...**

[Sitka EF678386](http://www.ncbi.nlm.nih.gov/nucleotide/148910125) 1255 TTGGTTTGGAAGATAAGAAAATTTCCAGGGCAGACAGAATCAACAATTAGTGCAGAAGTTGAACTGATTTCTACGATGGTGGAGAAGAAGGCCTGGACTCGGCCTCCGATTCAAATGGAG 1374

[White GO368075](http://www.ncbi.nlm.nih.gov/entrez/query.fcgi?cmd=Retrieve&db=Nucleotide&list_uids=225920080&dopt=GenBank&RID=0AVSPMRT112&log$=nuclalign&blast_rank=0) 551 ...............................................G........................................................................ 670

[Taeda DR057588](http://www.ncbi.nlm.nih.gov/entrez/query.fcgi?cmd=Retrieve&db=Nucleotide&list_uids=66981155&dopt=GenBank&RID=0AVSPMRT112&log$=nuclalign&blast_rank=0) 535 ...............................................G...........G..........................A..A.................A............ 416

[Taeda DR178693](http://www.ncbi.nlm.nih.gov/entrez/query.fcgi?cmd=Retrieve&db=Nucleotide&list_uids=68085136&dopt=GenBank&RID=0AVSPMRT112&log$=nuclalign&blast_rank=0) 311 ..............C.AG...........T........GG....C..G.......................C..T..A.CA...........T.....CA....A..A.....G...... 192

[Banksiana GW772395](http://www.ncbi.nlm.nih.gov/entrez/query.fcgi?cmd=Retrieve&db=Nucleotide&list_uids=292551601&dopt=GenBank&RID=0AVSPMRT112&log$=nuclalign&blast_rank=0) 393 ...............................................G......................................A..A.................A............ 512

**46630 F2...===================> <============================46630R2**

[Sitka EF678386](http://www.ncbi.nlm.nih.gov/nucleotide/148910125) 1375 TTTCAGGTTCCCATGTTCACGGCATCAGGTCTACGGGTCCGATTTTTGAAGGTTTGGGAAAAAAGTGGTTACAATACAGTTGAGTGGGTTCGCTACATCACAAGAGCTGGTTCATATGAG 1494

[White GO368075](http://www.ncbi.nlm.nih.gov/entrez/query.fcgi?cmd=Retrieve&db=Nucleotide&list_uids=225920080&dopt=GenBank&RID=0AVSPMRT112&log$=nuclalign&blast_rank=0) 671 ...............................................................................................TG....................... 790

[Taeda DR057588](http://www.ncbi.nlm.nih.gov/entrez/query.fcgi?cmd=Retrieve&db=Nucleotide&list_uids=66981155&dopt=GenBank&RID=0AVSPMRT112&log$=nuclalign&blast_rank=0) 415 ....................A........G...........T...................................C...........C..G..T.................T...... 296

[Taeda DR178693](http://www.ncbi.nlm.nih.gov/entrez/query.fcgi?cmd=Retrieve&db=Nucleotide&list_uids=68085136&dopt=GenBank&RID=0AVSPMRT112&log$=nuclalign&blast_rank=0) 191 ..C.................A.........T....T..G..G...........C........G................................T..........G....A........ 72

[Banksiana GW772395](http://www.ncbi.nlm.nih.gov/entrez/query.fcgi?cmd=Retrieve&db=Nucleotide&list_uids=292551601&dopt=GenBank&RID=0AVSPMRT112&log$=nuclalign&blast_rank=0) 513 ....................A........G...............................................C...........C..G..T.................T...... 632

STP ~570bp from polyA site

[Sitka EF678386](http://www.ncbi.nlm.nih.gov/nucleotide/148910125) 1495 ATTCGTTGT**TAG**GAGAAATTTTTAACCATTCTGGAACA-TTTTTCTTATTTAATTGTGCT 1553

[White GO368075](http://www.ncbi.nlm.nih.gov/entrez/query.fcgi?cmd=Retrieve&db=Nucleotide&list_uids=225920080&dopt=GenBank&RID=0AVSPMRT112&log$=nuclalign&blast_rank=0) 791 ......................................-..................... 849

[Taeda DR057588](http://www.ncbi.nlm.nih.gov/entrez/query.fcgi?cmd=Retrieve&db=Nucleotide&list_uids=66981155&dopt=GenBank&RID=0AVSPMRT112&log$=nuclalign&blast_rank=0) 295 .................T........T.......GG..T..............C...... 236

[Taeda DR178693](http://www.ncbi.nlm.nih.gov/entrez/query.fcgi?cmd=Retrieve&db=Nucleotide&list_uids=68085136&dopt=GenBank&RID=0AVSPMRT112&log$=nuclalign&blast_rank=0) 71 ..C..A..C.. 61

[Banksiana GW772395](http://www.ncbi.nlm.nih.gov/entrez/query.fcgi?cmd=Retrieve&db=Nucleotide&list_uids=292551601&dopt=GenBank&RID=0AVSPMRT112&log$=nuclalign&blast_rank=0) 633 .................T........T.......GG..T..............C...... 692

## HP

[Sitka spruce GH280347](http://www.ncbi.nlm.nih.gov/nucest/218443964) 250 TTTCAAGAGCAGATCAATTCTATAGCTATTTTCCAGCTGGCACGGAGCTCCTCTTTGATTCCACAAAGCTGTACAAAACAGCTTTGGGTAATGTATTCGAAGAGGAGGAATGGGGACCAA 369

[Sitka spruce FD730532](http://www.ncbi.nlm.nih.gov/entrez/query.fcgi?cmd=Retrieve&db=Nucleotide&list_uids=166897316&dopt=GenBank&RID=ZUTXT0KP11R&log$=nuclalign&blast_rank=0) 331 .......G................................................................................................................ 450

[White spruce DR574607](http://www.ncbi.nlm.nih.gov/entrez/query.fcgi?cmd=Retrieve&db=Nucleotide&list_uids=70639324&dopt=GenBank&RID=ZUTXT0KP11R&log$=nuclalign&blast_rank=0) 465 .......G................................................................................................................ 346

[White spruce EX310281](http://www.ncbi.nlm.nih.gov/entrez/query.fcgi?cmd=Retrieve&db=Nucleotide&list_uids=157509050&dopt=GenBank&RID=ZUTXT0KP11R&log$=nuclalign&blast_rank=0) 333 .......G................................................................................................................ 452

[White spruce GO360300](http://www.ncbi.nlm.nih.gov/entrez/query.fcgi?cmd=Retrieve&db=Nucleotide&list_uids=225910619&dopt=GenBank&RID=ZUTXT0KP11R&log$=nuclalign&blast_rank=0) 448 .......G...........T......................G....T..T.T............................................T...................... 329

[P banksiana GW754660](http://www.ncbi.nlm.nih.gov/entrez/query.fcgi?cmd=Retrieve&db=Nucleotide&list_uids=292532947&dopt=GenBank&RID=ZUTXT0KP11R&log$=nuclalign&blast_rank=0) 464 .......G..........................C........T..........................A..T.............................................. 345

[P taeda DR683214](http://www.ncbi.nlm.nih.gov/entrez/query.fcgi?cmd=Retrieve&db=Nucleotide&list_uids=70771690&dopt=GenBank&RID=ZUTXT0KP11R&log$=nuclalign&blast_rank=0) 472 .......G..........................C........T..........................A..T.............................................. 353

[P concorta GT242208](http://www.ncbi.nlm.nih.gov/entrez/query.fcgi?cmd=Retrieve&db=Nucleotide&list_uids=262137908&dopt=GenBank&RID=ZUTXT0KP11R&log$=nuclalign&blast_rank=0) 204 .......G....................C.....C........T..........................A..T.............................................. 323

**HPK3================================**>

**HPF1=============================**>**HPF2===============================**>

[Sitka spruce GH280347](http://www.ncbi.nlm.nih.gov/nucest/218443964) 370 TTGAATTTTCTATTATGTTAAAGCATTTCCAGCGCCAGAACAAGGCTCCTTATGCTTATCATGCTCAATACATGGTGCATCTTCTCTCAAATGGACTACTGGATGATGGTACAGGGTCAT 489

[Sitka spruce FD730532](http://www.ncbi.nlm.nih.gov/entrez/query.fcgi?cmd=Retrieve&db=Nucleotide&list_uids=166897316&dopt=GenBank&RID=ZUTXT0KP11R&log$=nuclalign&blast_rank=0) 451 .......................................G...............................................................................G 570

[White spruce DR574607](http://www.ncbi.nlm.nih.gov/entrez/query.fcgi?cmd=Retrieve&db=Nucleotide&list_uids=70639324&dopt=GenBank&RID=ZUTXT0KP11R&log$=nuclalign&blast_rank=0) 345 .......................................G....................................C..........................................G 226

[White spruce EX310281](http://www.ncbi.nlm.nih.gov/entrez/query.fcgi?cmd=Retrieve&db=Nucleotide&list_uids=157509050&dopt=GenBank&RID=ZUTXT0KP11R&log$=nuclalign&blast_rank=0) 453 .......................................G...............................................................................G 572

[White spruce GO360300](http://www.ncbi.nlm.nih.gov/entrez/query.fcgi?cmd=Retrieve&db=Nucleotide&list_uids=225910619&dopt=GenBank&RID=ZUTXT0KP11R&log$=nuclalign&blast_rank=0) 328 .........T.............................G...................................G....T..T.T.................................G 209

[P banksiana GW754660](http://www.ncbi.nlm.nih.gov/entrez/query.fcgi?cmd=Retrieve&db=Nucleotide&list_uids=292532947&dopt=GenBank&RID=ZUTXT0KP11R&log$=nuclalign&blast_rank=0) 344 .................G....................GG..........................................................T..---..............CG 228

[P taeda DR683214](http://www.ncbi.nlm.nih.gov/entrez/query.fcgi?cmd=Retrieve&db=Nucleotide&list_uids=70771690&dopt=GenBank&RID=ZUTXT0KP11R&log$=nuclalign&blast_rank=0) 352 .................G....................GG..........................................................T..---..............CG 236

[P concorta GT242208](http://www.ncbi.nlm.nih.gov/entrez/query.fcgi?cmd=Retrieve&db=Nucleotide&list_uids=262137908&dopt=GenBank&RID=ZUTXT0KP11R&log$=nuclalign&blast_rank=0) 324 .................G....................GG..........................................................T..---..............CG 440

<**=================================HPR1** <**=========**...**HPK2**

[Sitka spruce GH280347](http://www.ncbi.nlm.nih.gov/nucest/218443964) 490 CA**TAG**-----AAGGT---ACCTAAAGACATAAATAACATTGCAGAGACAATCCACACGACAAATAATATATGCTTTTATCAACATGATGACTGAGTTTATTGAATACTTTTGTAT 596

[Sitka spruce FD730532](http://www.ncbi.nlm.nih.gov/entrez/query.fcgi?cmd=Retrieve&db=Nucleotide&list_uids=166897316&dopt=GenBank&RID=ZUTXT0KP11R&log$=nuclalign&blast_rank=0) 571 .....-----.....---................................................................................................. 677

[White spruce DR574607](http://www.ncbi.nlm.nih.gov/entrez/query.fcgi?cmd=Retrieve&db=Nucleotide&list_uids=70639324&dopt=GenBank&RID=ZUTXT0KP11R&log$=nuclalign&blast_rank=0) 225 .....AAGGT.....---................................................................................................. 114

[White spruce EX310281](http://www.ncbi.nlm.nih.gov/entrez/query.fcgi?cmd=Retrieve&db=Nucleotide&list_uids=157509050&dopt=GenBank&RID=ZUTXT0KP11R&log$=nuclalign&blast_rank=0) 573 .....-----.....---................................................................................................. 679

[White spruce GO360300](http://www.ncbi.nlm.nih.gov/entrez/query.fcgi?cmd=Retrieve&db=Nucleotide&list_uids=225910619&dopt=GenBank&RID=ZUTXT0KP11R&log$=nuclalign&blast_rank=0) 208 .....-----.....---...................................C............................................................G 102

[P banksiana GW754660](http://www.ncbi.nlm.nih.gov/entrez/query.fcgi?cmd=Retrieve&db=Nucleotide&list_uids=292532947&dopt=GenBank&RID=ZUTXT0KP11R&log$=nuclalign&blast_rank=0) 227 ....AATGGC.....TTC.....................A............G...G.........C..GG.....C.A.G.......C.....T...................C 113

[P taeda DR683214](http://www.ncbi.nlm.nih.gov/entrez/query.fcgi?cmd=Retrieve&db=Nucleotide&list_uids=70771690&dopt=GenBank&RID=ZUTXT0KP11R&log$=nuclalign&blast_rank=0) 235 ....AATGGC.....TTC..................................G...G.........C..GG.....C.A.G......CC.........................C 121

[P concorta GT242208](http://www.ncbi.nlm.nih.gov/entrez/query.fcgi?cmd=Retrieve&db=Nucleotide&list_uids=262137908&dopt=GenBank&RID=ZUTXT0KP11R&log$=nuclalign&blast_rank=0) 441 ....AATGGC.....TTC..................................G...G.........C..GG.....C.A.G.......C.........................C 555

...**=========================HPK2**

[Sitka spruce GH280347](http://www.ncbi.nlm.nih.gov/nucest/218443964) 597 GGTTACAGATTACATTGTGAAGGCATATCTTGTAGAGCATTTAGTACTTTACTGaaaaaaaTAAGAAAGCATGAAATAAACAGCAAtttttttCCaaaaaaaaaaaaaaaaa 708

[Sitka spruce FD730532](http://www.ncbi.nlm.nih.gov/entrez/query.fcgi?cmd=Retrieve&db=Nucleotide&list_uids=166897316&dopt=GenBank&RID=ZUTXT0KP11R&log$=nuclalign&blast_rank=0) 678 ..C.....................................................C....................................................... 789

[White spruce DR574607](http://www.ncbi.nlm.nih.gov/entrez/query.fcgi?cmd=Retrieve&db=Nucleotide&list_uids=70639324&dopt=GenBank&RID=ZUTXT0KP11R&log$=nuclalign&blast_rank=0) 113 ..C...............................................G.....C............T.......................................... 2

[White spruce EX310281](http://www.ncbi.nlm.nih.gov/entrez/query.fcgi?cmd=Retrieve&db=Nucleotide&list_uids=157509050&dopt=GenBank&RID=ZUTXT0KP11R&log$=nuclalign&blast_rank=0) 680 ..C.....................................................C.....................................T...... 780

[White spruce GO360300](http://www.ncbi.nlm.nih.gov/entrez/query.fcgi?cmd=Retrieve&db=Nucleotide&list_uids=225910619&dopt=GenBank&RID=ZUTXT0KP11R&log$=nuclalign&blast_rank=0) 101 ..C..............G..........T...........................C....................................TT...... 1

[P banksiana GW754660](http://www.ncbi.nlm.nih.gov/entrez/query.fcgi?cmd=Retrieve&db=Nucleotide&list_uids=292532947&dopt=GenBank&RID=ZUTXT0KP11R&log$=nuclalign&blast_rank=0) 112 A.A.....T.....A...A..T...G.A.....G...T.C........C..T....C....C...G.T...........................T................ 1

[P taeda DR683214](http://www.ncbi.nlm.nih.gov/entrez/query.fcgi?cmd=Retrieve&db=Nucleotide&list_uids=70771690&dopt=GenBank&RID=ZUTXT0KP11R&log$=nuclalign&blast_rank=0) 120 ..A.....T.....A...A..T...G.A.....G...T.C........C..T....C....C...G.T......................... 28

[P concorta GT242208](http://www.ncbi.nlm.nih.gov/entrez/query.fcgi?cmd=Retrieve&db=Nucleotide&list_uids=262137908&dopt=GenBank&RID=ZUTXT0KP11R&log$=nuclalign&blast_rank=0) 556 ..A.....T.....A...A..T...G.A.....G...T.C........C..T....C....C...G.T......................... 648

## PTSR

**PTSR F1**=========================...

**PTSR K1**==...

[Sitka spruce EF083226](http://www.ncbi.nlm.nih.gov/nucleotide/116782616) 974 CCTCACCAGGAAAGCCTTATTGCATCTTGTTCCTACGATATGACAACATGCATGTGGGATTACAGAGCTCCAGAGGATGCTCTTCTAGCTCGATA**TGATCATCATACTGAGTTTGCTGTG** 1093

[White spruce EX391446](http://www.ncbi.nlm.nih.gov/entrez/query.fcgi?cmd=Retrieve&db=Nucleotide&list_uids=157590218&dopt=GenBank&RID=ZRY7YD49114&log$=nuclalign&blast_rank=0) 488 ........................................................................................................................ 607

[White spruce EX397078](http://www.ncbi.nlm.nih.gov/entrez/query.fcgi?cmd=Retrieve&db=Nucleotide&list_uids=157595850&dopt=GenBank&RID=ZRY7YD49114&log$=nuclalign&blast_rank=0) 492 ........................................................................................................................ 611

[P contorta GT269494](http://www.ncbi.nlm.nih.gov/entrez/query.fcgi?cmd=Retrieve&db=Nucleotide&list_uids=262162242&dopt=GenBank&RID=ZRY7YD49114&log$=nuclalign&blast_rank=0) 511 ..........................A.......................T...........T......................................................... 630

[P contorta GT269494](http://www.ncbi.nlm.nih.gov/entrez/query.fcgi?cmd=Retrieve&db=Nucleotide&list_uids=262136676&dopt=GenBank&RID=ZRY7YD49114&log$=nuclalign&blast_rank=0) 395 ..........................A.......................T...........T..................T...................................... 514

[P contorta GT243054](http://www.ncbi.nlm.nih.gov/entrez/query.fcgi?cmd=Retrieve&db=Nucleotide&list_uids=262139632&dopt=GenBank&RID=ZRY7YD49114&log$=nuclalign&blast_rank=0) 373 ..........................A.......................T...........TT........................................................ 492

[P taeda DR110695](http://www.ncbi.nlm.nih.gov/entrez/query.fcgi?cmd=Retrieve&db=Nucleotide&list_uids=67679972&dopt=GenBank&RID=ZRY7YD49114&log$=nuclalign&blast_rank=0) 450 ..........................A.......................T...........T......................................................... 331

[P banksiana GW771222](http://www.ncbi.nlm.nih.gov/entrez/query.fcgi?cmd=Retrieve&db=Nucleotide&list_uids=292556907&dopt=GenBank&RID=ZRY7YD49114&log$=nuclalign&blast_rank=0) 556 ..........................A.......................T...........T......................................................... 675

[P banksiana GW752578](http://www.ncbi.nlm.nih.gov/entrez/query.fcgi?cmd=Retrieve&db=Nucleotide&list_uids=292532545&dopt=GenBank&RID=ZRY7YD49114&log$=nuclalign&blast_rank=0) 477 ..........................A.......................T...........T......................................................... 358

[P banksiana GW767230](http://www.ncbi.nlm.nih.gov/entrez/query.fcgi?cmd=Retrieve&db=Nucleotide&list_uids=292553699&dopt=GenBank&RID=ZRY7YD49114&log$=nuclalign&blast_rank=0) 527 ..........................A.......................T...........T......................................................... 408

**PTSR F1...====>**  **<=======================PTSR R2**

**PTSR K1...===============================>** **<==========================PTSR R1** ~350bp from polyA

[Sitka spruce EF083226](http://www.ncbi.nlm.nih.gov/nucleotide/116782616) 1094 **GGAAT**TGATATAAGTGTTCTTGTGGAGGGTCTG**TTGGCAAGCACTGGATGGGATGAA**ACTGTCTATGTTTGGCAGCACGGAATGGATCCTCGAGCTTCT**TGA**GA-TGGGTATACTAGCTT 1212

[White spruce EX391446](http://www.ncbi.nlm.nih.gov/entrez/query.fcgi?cmd=Retrieve&db=Nucleotide&list_uids=157590218&dopt=GenBank&RID=ZRY7YD49114&log$=nuclalign&blast_rank=0) 608 ........................................................................................................-............... 726

[White spruce EX397078](http://www.ncbi.nlm.nih.gov/entrez/query.fcgi?cmd=Retrieve&db=Nucleotide&list_uids=157595850&dopt=GenBank&RID=ZRY7YD49114&log$=nuclalign&blast_rank=0) 612 ........................................................................................................-............... 730

[P contorta GT269494](http://www.ncbi.nlm.nih.gov/entrez/query.fcgi?cmd=Retrieve&db=Nucleotide&list_uids=262162242&dopt=GenBank&RID=ZRY7YD49114&log$=nuclalign&blast_rank=0) 631 ................................A............................................T...................G......C.....G......... 750

[P contorta GT269494](http://www.ncbi.nlm.nih.gov/entrez/query.fcgi?cmd=Retrieve&db=Nucleotide&list_uids=262136676&dopt=GenBank&RID=ZRY7YD49114&log$=nuclalign&blast_rank=0) 515 ..................T.............A............................................T...................G......C.....G......... 634

[P contorta GT243054](http://www.ncbi.nlm.nih.gov/entrez/query.fcgi?cmd=Retrieve&db=Nucleotide&list_uids=262139632&dopt=GenBank&RID=ZRY7YD49114&log$=nuclalign&blast_rank=0) 493 ................................A............................................T...................G......C.....G......... 612

[P taeda DR110695](http://www.ncbi.nlm.nih.gov/entrez/query.fcgi?cmd=Retrieve&db=Nucleotide&list_uids=67679972&dopt=GenBank&RID=ZRY7YD49114&log$=nuclalign&blast_rank=0) 330 .............................................................................T...................G......C.....G......... 211

[P banksiana GW771222](http://www.ncbi.nlm.nih.gov/entrez/query.fcgi?cmd=Retrieve&db=Nucleotide&list_uids=292556907&dopt=GenBank&RID=ZRY7YD49114&log$=nuclalign&blast_rank=0) 676 .............................................................................T...................G......C.....G......... 795

[P banksiana GW752578](http://www.ncbi.nlm.nih.gov/entrez/query.fcgi?cmd=Retrieve&db=Nucleotide&list_uids=292532545&dopt=GenBank&RID=ZRY7YD49114&log$=nuclalign&blast_rank=0) 357 .....A.......................................................................T...................G......C.....G......... 238

[P banksiana GW767230](http://www.ncbi.nlm.nih.gov/entrez/query.fcgi?cmd=Retrieve&db=Nucleotide&list_uids=292553699&dopt=GenBank&RID=ZRY7YD49114&log$=nuclalign&blast_rank=0) 407 .....A.......................................................................T..............G....G......C.....G......... 288

**<=============================PTSR K2**

[Sitka spruce EF083226](http://www.ncbi.nlm.nih.gov/nucleotide/116782616) 1213 GGTGTTTCCTTCTCAGGTTACTGGTTTGGTGTTTTACTGCCTGTCCAAGGGCACAGGAAGTGGCTTGTGTCCATTTCAGATAGGCATTTACAGTTTATTTA-TATTGGACTTGTAAAGAT 1331

[White spruce EX391446](http://www.ncbi.nlm.nih.gov/entrez/query.fcgi?cmd=Retrieve&db=Nucleotide&list_uids=157590218&dopt=GenBank&RID=ZRY7YD49114&log$=nuclalign&blast_rank=0) 727 .....................................................................................................-.................. 845

[White spruce EX397078](http://www.ncbi.nlm.nih.gov/entrez/query.fcgi?cmd=Retrieve&db=Nucleotide&list_uids=157595850&dopt=GenBank&RID=ZRY7YD49114&log$=nuclalign&blast_rank=0) 731 .....................................................................................................-.................. 849

[P contorta GT269494](http://www.ncbi.nlm.nih.gov/entrez/query.fcgi?cmd=Retrieve&db=Nucleotide&list_uids=262162242&dopt=GenBank&RID=ZRY7YD49114&log$=nuclalign&blast_rank=0) 751 ..CC.....C......A.............C.......T...C...G...ATG......C..TT...CA.......T......C........T....C...T.................. 870

[P contorta GT269494](http://www.ncbi.nlm.nih.gov/entrez/query.fcgi?cmd=Retrieve&db=Nucleotide&list_uids=262136676&dopt=GenBank&RID=ZRY7YD49114&log$=nuclalign&blast_rank=0) 635 ..CC.....C......A.............C.......T...C...G...ATG......C..TT...CA.......T......C........T....C...T.................. 754

[P contorta GT243054](http://www.ncbi.nlm.nih.gov/entrez/query.fcgi?cmd=Retrieve&db=Nucleotide&list_uids=262139632&dopt=GenBank&RID=ZRY7YD49114&log$=nuclalign&blast_rank=0) 613 ..CC.....C......A.............C.......T...C...G...ATG......C..TT...CA.......T......C........T....C...T.................. 732

[P taeda DR110695](http://www.ncbi.nlm.nih.gov/entrez/query.fcgi?cmd=Retrieve&db=Nucleotide&list_uids=67679972&dopt=GenBank&RID=ZRY7YD49114&log$=nuclalign&blast_rank=0) 210 ..CC.....C......A.............C.......T...C...G...ATG..N...C..TT...CA.......T......C........T....C...T.................. 91

[P banksiana GW771222](http://www.ncbi.nlm.nih.gov/entrez/query.fcgi?cmd=Retrieve&db=Nucleotide&list_uids=292556907&dopt=GenBank&RID=ZRY7YD49114&log$=nuclalign&blast_rank=0) 796 ..CC.....C.... 809

[P banksiana GW752578](http://www.ncbi.nlm.nih.gov/entrez/query.fcgi?cmd=Retrieve&db=Nucleotide&list_uids=292532545&dopt=GenBank&RID=ZRY7YD49114&log$=nuclalign&blast_rank=0) 237 ..CC.....C......A.............C.......T...C...G...ATG......C..TT...CA.......T......C........T....C...T.................. 118

[P banksiana GW767230](http://www.ncbi.nlm.nih.gov/entrez/query.fcgi?cmd=Retrieve&db=Nucleotide&list_uids=292553699&dopt=GenBank&RID=ZRY7YD49114&log$=nuclalign&blast_rank=0) 287 ..CC.....C......A.............C.......T...C...G...ATG......C..TT...CA.......T......C........T....C...T.................. 168

## UBC1

[Sitka DR503425](http://www.ncbi.nlm.nih.gov/nucest/DR503425.1) 371 GGACTAAAGTTTTCCACCCAAATATAAATAACAATGGAAGTATCTGCCTTGACATCTTAAAGGAACAGTGGAGTCCTGCTTTGACAATCTCCAAGGTCTTGCTTTCAATTTGCTCTTTGT 490

[Sitka DR495127](http://www.ncbi.nlm.nih.gov/entrez/query.fcgi?cmd=Retrieve&db=Nucleotide&list_uids=69452679&dopt=GenBank&RID=00C2GJB911R&log$=nuclalign&blast_rank=0) 470 ........................................................................................................................ 351

[White EX427103](http://www.ncbi.nlm.nih.gov/entrez/query.fcgi?cmd=Retrieve&db=Nucleotide&list_uids=157626021&dopt=GenBank&RID=00C2GJB911R&log$=nuclalign&blast_rank=0) 352 ........................................................................................................................ 471

[White EX409754](http://www.ncbi.nlm.nih.gov/entrez/query.fcgi?cmd=Retrieve&db=Nucleotide&list_uids=157608530&dopt=GenBank&RID=00C2GJB911R&log$=nuclalign&blast_rank=0) 362 ........................................................................................................................ 481

[Norway AY639585](http://www.ncbi.nlm.nih.gov/entrez/query.fcgi?cmd=Retrieve&db=Nucleotide&list_uids=54288725&dopt=GenBank&RID=00C2GJB911R&log$=nuclalign&blast_rank=0) 98 ........................................................................................................................ 217

[Contorta GT241619](http://www.ncbi.nlm.nih.gov/entrez/query.fcgi?cmd=Retrieve&db=Nucleotide&list_uids=262130122&dopt=GenBank&RID=00C2GJB911R&log$=nuclalign&blast_rank=0) 365 ..........................................................G......................................T.....C................ 484

[Banksiana GW768240](http://www.ncbi.nlm.nih.gov/entrez/query.fcgi?cmd=Retrieve&db=Nucleotide&list_uids=292548944&dopt=GenBank&RID=00C2GJB911R&log$=nuclalign&blast_rank=0) 366 ..........................................................G......................................T.....C................ 485

[Taeda DR384469](http://www.ncbi.nlm.nih.gov/entrez/query.fcgi?cmd=Retrieve&db=Nucleotide&list_uids=68252067&dopt=GenBank&RID=00C2GJB911R&log$=nuclalign&blast_rank=0) 337 ..........................................................G......................................T.....C................ 456

[Pinaster CT583378](http://www.ncbi.nlm.nih.gov/entrez/query.fcgi?cmd=Retrieve&db=Nucleotide&list_uids=90041651&dopt=GenBank&RID=00C2GJB911R&log$=nuclalign&blast_rank=0) 336 .................................................C........G......................................T.....C.....A.......... 455

[Radiata FE520923](http://www.ncbi.nlm.nih.gov/entrez/query.fcgi?cmd=Retrieve&db=Nucleotide&list_uids=222829551&dopt=GenBank&RID=00C2GJB911R&log$=nuclalign&blast_rank=0) 280 ..........................................................G......................................T.....C................ 399

[Douglas CN640308](http://www.ncbi.nlm.nih.gov/entrez/query.fcgi?cmd=Retrieve&db=Nucleotide&list_uids=47151385&dopt=GenBank&RID=00C2GJB911R&log$=nuclalign&blast_rank=0) 241 ....A..............G..C...................................G....................A..A..............A...................... 360

**UBC1F1======> <===========================UBC1R2 <===========================UBC1R1**

[Sitka DR503425](http://www.ncbi.nlm.nih.gov/nucest/DR503425.1) 491 TGACGGATCCAAACCCAGATGATCCTCTTGTACCAGAGATTGCTCATATGTACAAGACTGACAGGGGCAAATATGAGTCCACCGCAAGGAGTTGGACTCAGAAGTATGCAATGGGT**TAG**C 610

[Sitka DR495127](http://www.ncbi.nlm.nih.gov/entrez/query.fcgi?cmd=Retrieve&db=Nucleotide&list_uids=69452679&dopt=GenBank&RID=00C2GJB911R&log$=nuclalign&blast_rank=0) 350 ......................................................................................................................A. 231

[White EX427103](http://www.ncbi.nlm.nih.gov/entrez/query.fcgi?cmd=Retrieve&db=Nucleotide&list_uids=157626021&dopt=GenBank&RID=00C2GJB911R&log$=nuclalign&blast_rank=0) 472 ........................................................................................................................ 591

[White EX409754](http://www.ncbi.nlm.nih.gov/entrez/query.fcgi?cmd=Retrieve&db=Nucleotide&list_uids=157608530&dopt=GenBank&RID=00C2GJB911R&log$=nuclalign&blast_rank=0) 482 ................................................C............................................................G.......... 601

[Norway AY639585](http://www.ncbi.nlm.nih.gov/entrez/query.fcgi?cmd=Retrieve&db=Nucleotide&list_uids=54288725&dopt=GenBank&RID=00C2GJB911R&log$=nuclalign&blast_rank=0) 218 ........................................................................................................................ 337

[Contorta GT241619](http://www.ncbi.nlm.nih.gov/entrez/query.fcgi?cmd=Retrieve&db=Nucleotide&list_uids=262130122&dopt=GenBank&RID=00C2GJB911R&log$=nuclalign&blast_rank=0) 485 .............................................................T....................T...C................A..............A. 604

[Banksiana GW768240](http://www.ncbi.nlm.nih.gov/entrez/query.fcgi?cmd=Retrieve&db=Nucleotide&list_uids=292548944&dopt=GenBank&RID=00C2GJB911R&log$=nuclalign&blast_rank=0) 486 ................T............................................T....................T...C................A..............A. 605

[Taeda DR384469](http://www.ncbi.nlm.nih.gov/entrez/query.fcgi?cmd=Retrieve&db=Nucleotide&list_uids=68252067&dopt=GenBank&RID=00C2GJB911R&log$=nuclalign&blast_rank=0) 457 .............................................................T....................T...C................A..............A. 576

[Pinaster CT583378](http://www.ncbi.nlm.nih.gov/entrez/query.fcgi?cmd=Retrieve&db=Nucleotide&list_uids=90041651&dopt=GenBank&RID=00C2GJB911R&log$=nuclalign&blast_rank=0) 456 .............................................................T....................T...C................A..............A. 575

[Radiata FE520923](http://www.ncbi.nlm.nih.gov/entrez/query.fcgi?cmd=Retrieve&db=Nucleotide&list_uids=222829551&dopt=GenBank&RID=00C2GJB911R&log$=nuclalign&blast_rank=0) 400 .............................................................T....................T...C................A..............A. 519

[Douglas CN640308](http://www.ncbi.nlm.nih.gov/entrez/query.fcgi?cmd=Retrieve&db=Nucleotide&list_uids=47151385&dopt=GenBank&RID=00C2GJB911R&log$=nuclalign&blast_rank=0) 361 ..........................................................A.......C...............T..................................... 480

[Sitka DR503425](http://www.ncbi.nlm.nih.gov/nucest/DR503425.1) 611 TTTTAGAAACTATATAGCAGTGATGGAACTTTAGCCCTAAGTTGGAATTTCTTCGAAGTCAATTACTTGTTTGCTTGTAAGAAATGTTTTCTTAAGATAAATGCTTTCTCTAAACTTGa 729

[Sitka DR495127](http://www.ncbi.nlm.nih.gov/entrez/query.fcgi?cmd=Retrieve&db=Nucleotide&list_uids=69452679&dopt=GenBank&RID=00C2GJB911R&log$=nuclalign&blast_rank=0) 230 ....................................................................................................................... 112

[White EX427103](http://www.ncbi.nlm.nih.gov/entrez/query.fcgi?cmd=Retrieve&db=Nucleotide&list_uids=157626021&dopt=GenBank&RID=00C2GJB911R&log$=nuclalign&blast_rank=0) 592 ....................................................................................................................... 710

[White EX409754](http://www.ncbi.nlm.nih.gov/entrez/query.fcgi?cmd=Retrieve&db=Nucleotide&list_uids=157608530&dopt=GenBank&RID=00C2GJB911R&log$=nuclalign&blast_rank=0) 602 ....................................................................................................................... 720

[Norway AY639585](http://www.ncbi.nlm.nih.gov/entrez/query.fcgi?cmd=Retrieve&db=Nucleotide&list_uids=54288725&dopt=GenBank&RID=00C2GJB911R&log$=nuclalign&blast_rank=0) 338 ....................................................................................................................... 456

[Contorta GT241619](http://www.ncbi.nlm.nih.gov/entrez/query.fcgi?cmd=Retrieve&db=Nucleotide&list_uids=262130122&dopt=GenBank&RID=00C2GJB911R&log$=nuclalign&blast_rank=0) 605 -....A..........T................T..............C..............G....................................G.................. 722

[Banksiana GW768240](http://www.ncbi.nlm.nih.gov/entrez/query.fcgi?cmd=Retrieve&db=Nucleotide&list_uids=292548944&dopt=GenBank&RID=00C2GJB911R&log$=nuclalign&blast_rank=0) 606 -.C..A..........T................T............G.C..............G....................................G.................. 723

[Taeda DR384469](http://www.ncbi.nlm.nih.gov/entrez/query.fcgi?cmd=Retrieve&db=Nucleotide&list_uids=68252067&dopt=GenBank&RID=00C2GJB911R&log$=nuclalign&blast_rank=0) 577 -....A..........T................T..............C..............G....................................G.................. 694

[Pinaster CT583378](http://www.ncbi.nlm.nih.gov/entrez/query.fcgi?cmd=Retrieve&db=Nucleotide&list_uids=90041651&dopt=GenBank&RID=00C2GJB911R&log$=nuclalign&blast_rank=0) 576 -....A..........T.....................-G.......................G....................................G.................. 692

[Radiata FE520923](http://www.ncbi.nlm.nih.gov/entrez/query.fcgi?cmd=Retrieve&db=Nucleotide&list_uids=222829551&dopt=GenBank&RID=00C2GJB911R&log$=nuclalign&blast_rank=0) 520 -....A..........T................T..............C..............G....................................G.................. 637

[Douglas CN640308](http://www.ncbi.nlm.nih.gov/entrez/query.fcgi?cmd=Retrieve&db=Nucleotide&list_uids=47151385&dopt=GenBank&RID=00C2GJB911R&log$=nuclalign&blast_rank=0) 481 ...A.A..........T....A.........G........T.......A.....A........G............................A.......G..........G....... 599
